# Indoloquinoline alkaloid neocryptolepine derivative inhibits *Botrytis cinerea* by targeting thiamine thiazole synthase

**DOI:** 10.1126/sciadv.adq5329

**Published:** 2025-03-12

**Authors:** Wen-Bin Zhao, Jun-Xia An, Ya-Rui Jin, Chen-Xin Jing, Shao-Yong Zhang, Hong-Jie Liang, Tian-Li Dai, Xiong-Fei Luo, Bao-Qi Zhang, Zhi-Jun Zhang, Ying-Qian Liu

**Affiliations:** ^1^ School of Pharmacy, Lanzhou University, Lanzhou, Gansu 730000, China.; ^2^ Key Laboratory of Vector Biology and Pathogen Control of Zhejiang Province, College of Life Science, Huzhou University, Huzhou, Zhejiang 313000, China.

## Abstract

The emergence and rapid spread of multidrug-resistant *Botrytis cinerea* strains pose a great challenge to the quality and safety of agricultural products and the efficient use of pesticides. Previously unidentified fungicides and targets are urgently needed to combat *B. cinerea*–associated infections as alternative therapeutic options. In this study, the promising compound Z24 demonstrated efficacy against all tested plant pathogenic fungi. Thiamine thiazole synthase (Bcthi4) was identified as a target protein of Z24 by drug affinity responsive target stability (DARTS), cellular thermal shift assay (CETSA), and surface plasmon resonance (SPR) assays. Molecular docking and enzyme activity experiments have demonstrated that Z24 can affect the function of Bcthi4. Last, mechanistic studies show that Z24 inhibits thiamine biosynthesis by binding to Bcthi4 and induces up-regulation of alternative splicing [alternative 5′ splice site (A5SS)] of the *Bcthi4* gene. In conclusion, by targeting Bcthi4, Z24 has the potential to be developed as a previously unidentified anti–*B. cinerea* candidate.

## INTRODUCTION

*Botrytis cinerea* is a necrotrophic plant pathogenic fungus with a broad host range that causes large economic losses to crops (*1*). Because about 85% of fungicides now on the market target single enzymes, this is particularly alarming. The emergence of resistance to azoles, succinate dehydrogenase inhibitors (SDHIs), and quinone outside inhibitors (strobilurins) poses a serious threat to agricultural security (*2*–*4*). Therefore, the identification of potential fungicides with previously unknown targets and low toxicity is urgently needed to combat *B. cinerea* infections.

On earth, plants make up most of biomass. Plant-derived compounds have been widely used in the treatment of various infections (*5*, *6*). Neocryptolepine, an indoloquinoline alkaloid with a wide range of pharmacological activities, was isolated from the traditional African herb *Cryptolepis sanguinolenta*. Neocryptolepine and its derivatives exhibit excellent antifungal activity against plant pathogenic fungi, while compound Z24 exhibits the most effective inhibitory potency against *B. cinerea*, as initially identified in our prior investigation (*7*). However, there no studies have been done on its antifungal properties and potential target, especially against *B. cinerea*, to date.

Thiamine thiazole synthase (Thi4) is an intracellular membrane–bound protein and serves as the potential target. It is a core protein involved in the biological and metabolic processes of *Fusarium solani*. Furthermore, the Thi4 protein exists in the thiamine thiazole biosynthesis pathway, which is unique to pathogens and deficient in humans (*8*). Thiamine biosynthesis in fungi has been extensively studied in *Aspergillus* species, and inhibiting thiamine biosynthesis by targeting thiamine uptake has been proposed as an effective strategy for antifungal development (*9*). Thiamine, an important cofactor required for the growth of fungi, is produced by the Thi6p-dependent coupling of thiazole and pyrimidine (*10*). The pyrimidine moiety is synthesized from 5-aminoimidazole ribotide by 4-amino-2-methyl-5-phosphomethylpyrimidine phosphate synthase (THIC) in prokaryotes, plants, and green algae, whereas in fungi, it is synthesized from pyridoxal-5-phosphate and histidine by thiamine biosynthesis protein 5/N-myristoyltransferase 1 (THI5/NMT1) (*11*). 4-Methyl-5-(2-phosphooxyethyl)thiazole is produced in eubacteria via ThiG from iminoglycine, pyruvate, glyceraldehyde-3-phosphate, and cysteine. In archaea, fungi, plants, and green algae, it is produced via THI1/THI4 using nicotinamide adenine dinucleotide (oxidized form), glycine, and a sulfur atom from a cysteine residue in the active site (*12*). The biosynthesis of the adenylated carboxythiazole (ADT) precursor of thiamin is chemically complex and energetically expensive (*13*). Plants, fungi, and some prokaryotes make ADT via the thiazole synthase THI4, a single-turnover suicide enzyme (*14*, *15*), which is also true for THI5/NMT1 (*16*). Thiamine pyrophosphate (TPP) is a cofactor required for the carboxylation and decarboxylation of various metabolic intermediates in carbohydrate and amino acid metabolism. It acts as a cofactor for key enzymes such as pyruvate dehydrogenase, transketolase, and pyruvate decarboxylase and is central to the activity of several enzymes, particularly those involved in the metabolism of glucose (*13*, *17*). The TPP riboswitch is one of the most common and abundant riboswitches in bacteria (*18*). It is also the main riboswitch found in eukaryotes, such as fungi (*19*), algae, and plants (*20*, *21*). The active form of thiamine, TPP, is produced when thiamine is pyrophosphorylated by the enzyme thiamine pyrophosphokinase (*22*). Thiamine enters cells and is phosphorylated to generate TPP, and the resulting coenzyme serves as a ligand for riboswitch-mediated control of RNA splicing in fungi (*23*). The filamentous fungus *Neurospora crassa* has three riboswitches, two of which are located in introns within the 5′ untranslated region of the thiamin synthesis genes *THI4* (*NCU06110*) and *THI5* (also known as *NMT1* or *NCU09345*) (*24*, *25*). Eukaryotic riboswitches are often situated within introns, where they function by regulating splicing. TPP riboswitches regulate the expression of thiamin synthesis genes in algae and marine phytoplankton (*26*), plants (*27*, *28*), filamentous fungi (*24*, *25*), and probably oomycetes (*29*). TPP riboswitches have been experimentally validated in a few species of fungi where they are involved in splicing and regulate the expression of TPP biosynthesis (*24*, *30*) and transporter (*25*) genes. However, the role of *Bcthi4* in *B. cinerea* remains unclear to date.

Here, we found that Z24 is a potent fungicide candidate targeting Bcthi4 and exhibited excellent antifungal activity against *B. cinerea*. Our study first elucidated the mechanism of the neocryptolepine derivative Z24 and identified a promising lead for the development of anti–*B. cinerea* fungicides.

## RESULTS

### The candidate compound Z24 was evaluated for its activity in vitro

**Fig. 1.**
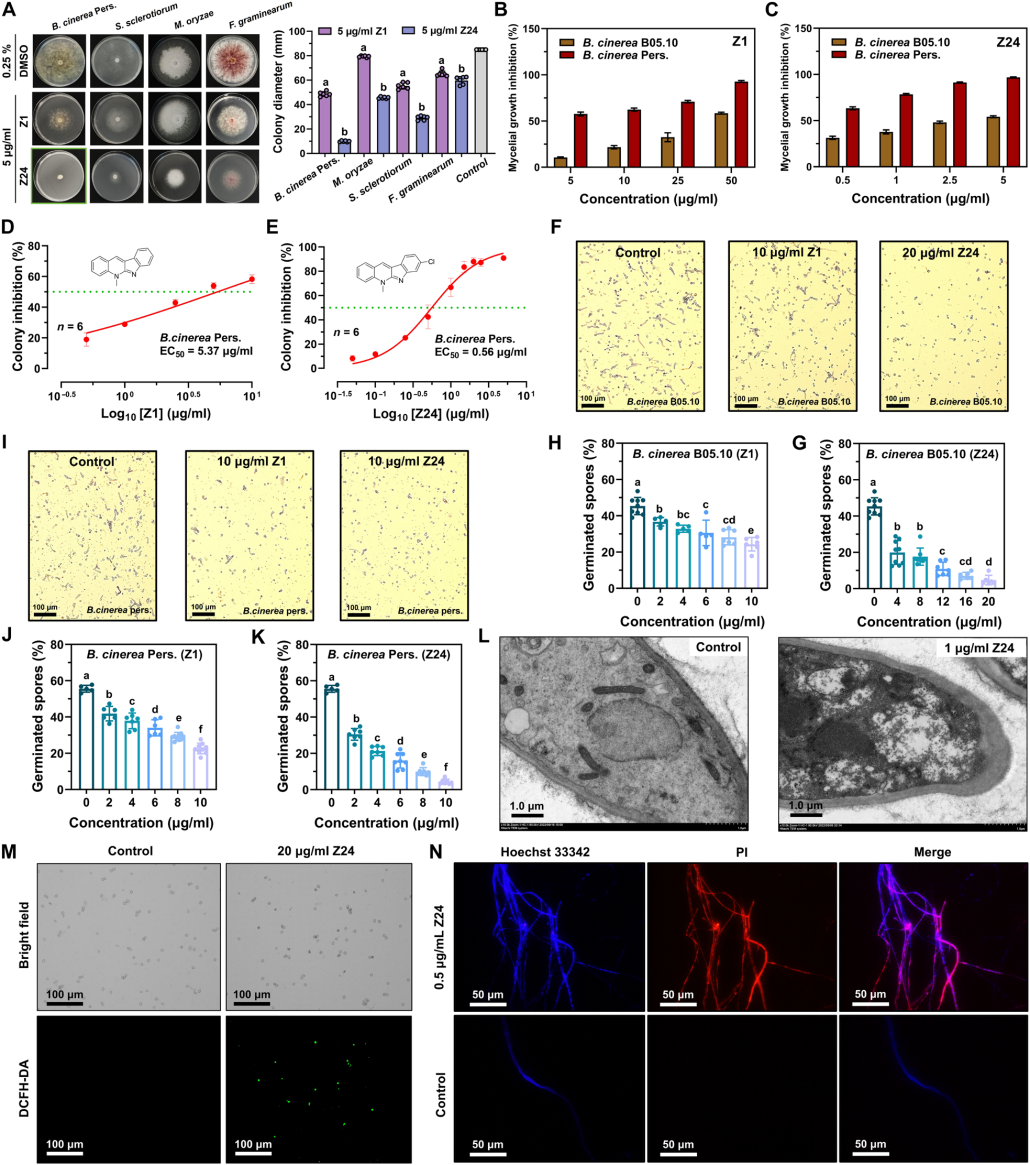
In vitro antifungal activity of Z24 against *B. cinerea*. (**A**) Colony formation (*n* = 6) of phytopathogenic fungi after 3 to 7 days of growth on agar plates supplemented with Z1 and Z24 (5 μg/ml). (**B**) Compound Z1 moderately suppressed mycelial growth of *B. cinerea* Pers. and *B. cinerea* B05.10 at concentrations ranging from 5 to 50 μg/ml. (**C**) Compound Z24 effectively suppressed mycelial growth of *B. cinerea* Pers. and *B. cinerea* B05.10 at concentrations of 0.5 to 5 μg/ml. (**D** and **E**) Colony formation (*n* = 6) of *B. cinerea* Pers. after 4 days of growth on agar plates, supplemented with increasing concentrations of compound Z1 and Z24. The green dotted line indicates EC_50_ concentration. (**F**) Representative images of the germination of *B. cinerea* B05.10 spores after treatment with Z1 and Z24. Scale bars, 100 μm. (**G** and **H**) Effects of (G) Z24 (*n* = 6 to 8) and (H) Z1 (*n* = 5 to 7) on the germination of *B. cinerea* B05.10 spores. (**I**) Representative images of the germination of *B. cinerea* Pers. spores after treatment with Z1 and Z24. Scale bars, 100 μm. (**J** and **K**) Effects of (J) Z1 (*n* = 6 to 10) and (K) Z24 (*n* = 7 to 8) on the germination of *B. cinerea* Pers. spores. (**L**) Transmission electron microscopy (TEM) images reveal the ultrastructure of *B. cinerea* Pers. after treatment with Z24 (1 μg/ml). Scale bars, 1.0 μm. (**M**) Intracellular ROS levels [2′,7′-dichlorodihydrofluorescein diacetate (DCFH-DA)] in *B. cinerea* Pers. spores after treatment with Z24 (20 μg/ml) were measured using DCFH-DA. Scale bars, 100 μm. (**N**) Nuclear dye propidium iodide (PI)/Hoechst 33342 was used to stain the mycelial cells treated with Z24 (0.5 μg/ml). Scale bars, 50 μm.

Neocryptolepine (Z1) and derivative Z24 were evaluated for their antifungal activity against six different plant pathogenic fungi, including *B. cinerea* Pers., *Sclerotinia sclerotiorum*, *FusaHum graminearum*, *Fusarium oxysporum*, *Rhizoctonia solani*, and *Phytophthora capsici*, taking pyrimethanil, boscalid, thiophanate-methyl, carbendazim, and azoxystrobin as positive controls, and their half maximal effective concentration (EC_50_) values against the tested plant pathogenic fungi are listed in Table 1. As indicated by the EC_50_ values, the findings demonstrated a notable enhancement in the activity of the modified derivative Z24. Specifically, Z24 showed notably lower EC_50_ values, ranging from 0.52 to 4.93 μg/ml. In comparison, the positive control drugs showed EC_50_ values ranging from 0.65 to 13.19 μg/ml. As a result, compound Z24 was chosen as a representative for further investigation due to its superior antifungal potency. As illustrated in Fig. 1A, Z24 exhibited superior inhibitory efficacy against *S. sclerotiorum*, *B. cinerea* Pers., *F. graminearum*, and *F. oxysporum* at a concentration of 5 μg/ml. The antifungal activity of Z24 against *B. cinerea* Pers. and *B. cinerea* B05.10 was over 10-fold greater than that of Z1, as seen in Fig. 1 (B and C). For *B. cinerea* Pers., the EC_50_ values of compounds Z1 and Z24 were 5.37 and 0.56 μg/ml, respectively (Fig. 1, D and E). As shown in Table 1, compound Z24 was more effective than pyrimethanil (EC_50_ of 4.45 μg/ml), a commercially available fungicide, against *B. cinerea* Pers. Additionally, compound Z24 exhibited noteworthy inhibition of *B. cinerea* spore germination (Fig. 1 F and I). The germination rates of *B. cinerea* B05.10 spores were 36.69, 32.86, 30.56, 28.14, and 24.28%, following Z1 treatment, as shown in Fig. 1H. However, after Z24 treatment, the germination rate dropped to 19.93, 17.63, 10.87, 7.11, and 4.74% (Fig. 1G). Meanwhile, *B. cinerea* Pers. spores showed germination rates of 41.82, 37.89, 34.06, 29.20, and 22.63%, following Z1 treatment (Fig. 1J). When Z24 treatment was applied, however, the germination rates decreased to 30.48, 21.29, 16.09, 9.95, and 3.54% (Fig. 1K). To assess the anti–*B. cinerea* mechanism of Z24, we preliminarily investigated the structural integrity and oxidative state of *B. cinerea*. Examination of the Z24-treated mycelium cell ultrastructure by transmission electron microscopy (TEM) revealed thicker mycelium cell walls, organelle degradation, reduced cytoplasmic vacuoles, disorganized cytoplasm, and disrupted mitochondrial structures (Fig. 1L). The intracellular reactive oxygen species (ROS) was measured using a fluorescent probe, 2′,7′-dichlorodihydrofluorescein diacetate (DCFH-DA). Observation under a fluorescence microscope clearly confirmed that Z24 treatment exhibited a strong ability to generate intracellular ROS in *B. cinerea* compared to the control group (Fig. 1M). To assess the toxicity of Z24 (0.5 μg/ml) on *B. cinerea* cells in potato dextrose agar (PDA) medium, we used the nuclear dye PI/Hoechst 33342 cell stain to stain the treated cells. Adding the membrane-impermeable “propidium iodide (PI) dye” and membrane-permeable “Hoechst 33342 dye” caused dying or dead cells to turn bright red or bright blue, while live cells were fluorescent baby blue (Fig. 1N).

**Table 1.** Fungicidal activity of Z1/Z24.

Fungus	EC_50_ (μg/ml)		
	Z1	Z24	Positive control
*Botrytis cinerea* Pers.	5.37	0.56	4.45/Pyrimethanil
*Sclerotinia sclerotiorum*	17.65	2.80	1.94/Boscalid
*Rhizoctonia solani*	9.00	4.93	6.76/Thiophanate methyl
*FusaHum graminearum*	16.31	1.92	0.65/Carbendazim
*Fusarium oxysporum*	17.63	2.82	12.53/Azoxystrobin
*Phytophthora capsici*	37.15	0.52	13.19/Azoxystrobin

### The direct targets of Z24 against *B. cinerea* were identified and validated

**Fig. 2.**
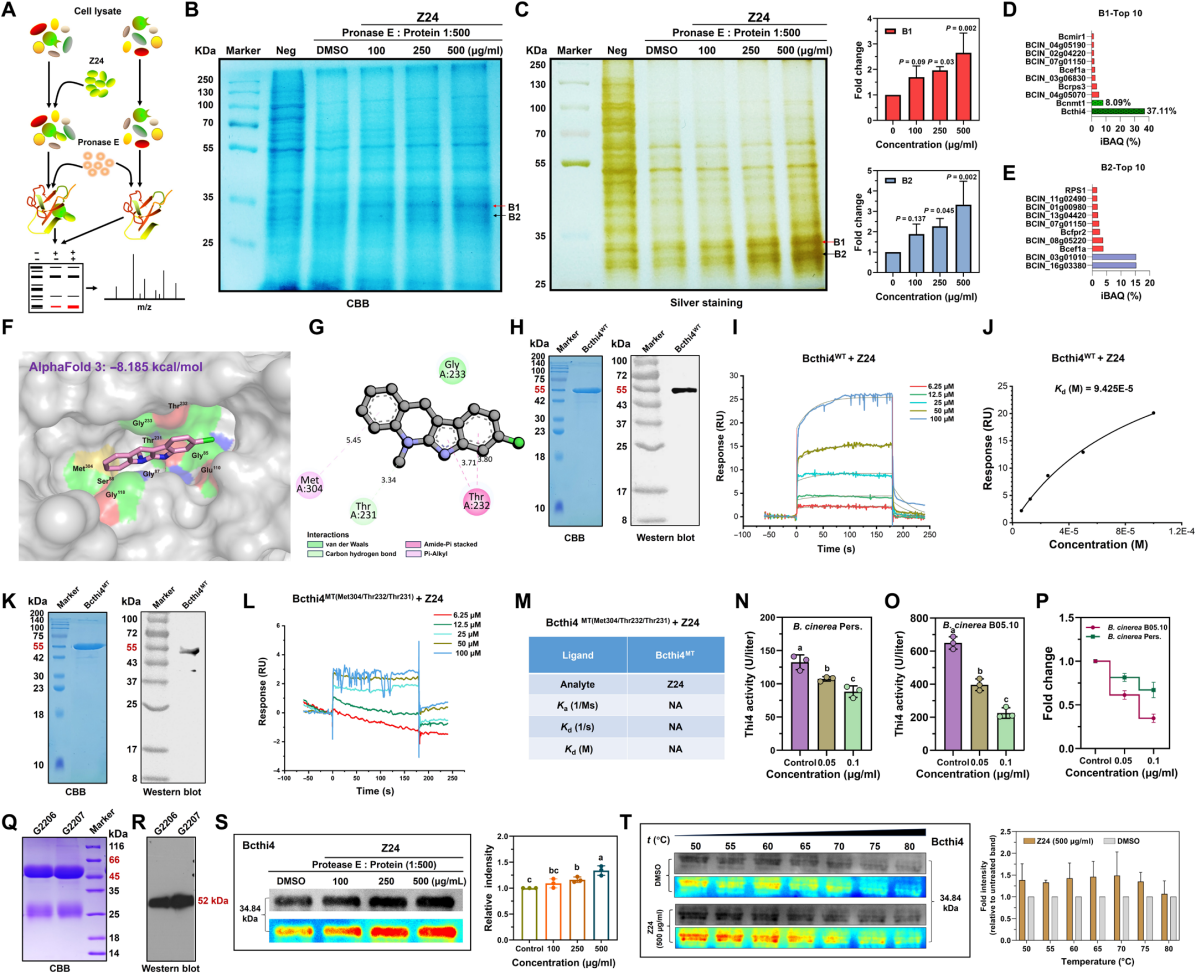
Z24 targets the Bcthi4 protein, which contributes to inhibit the growth of *B. cinerea* cells in vitro and in vivo. (**A**) DARTS identifies a molecular target of Z24. (**B**) *B. cinerea* Pers. cell lysates were incubated with Z24 in vitro, followed by protease digestion and Coomassie bright blue (CBB) stain. (**C**) *B. cinerea* Pers. cell lysates were incubated with Z24 in vitro, followed by protease digestion and silver staining (*n* = 3 exp.). The red and black arrows indicate the locations of the different bands, B1 and B2. (**D** and **E**) Information on the top 10 proteins with the highest relative abundance in the B1 and B2 band. iBAQ, intensity-based absolute quantification. (**F** and **G**) Predicted binding mode of Z24 with Bcthi4. (**H**) Expression and purification of the Bcthi4^WT^ protein in a prokaryotic expression system. (**I**) Surface plasmon resonance (SPR) sensorgrams obtained from Bcthi4^WT^-coated chips at different concentrations of Z24. (**J**) Dissociation constant (*K*_d_) value of Z24 binding to the recombinant Bcthi4^WT^ protein. (**K**) Expression and purification of the Bcthi4^MT(Met304/Thr232/Thr231)^ mutant protein in a prokaryotic expression system. (**L**) Detection of the binding of Bcthi4^MT(Met304/Thr232/Thr231)^ to Z24 by SPR analysis. (**M**) *K*_d_ value of Z24 binding to the recombinant mutated Bcthi4^MT(Met304/Thr232/Thr231)^ protein. “NA” indicates that no binding activity was detected. *K*_*a*_, acid constant. (**N** and **O**) Bcthi4 activity in *B. cinerea* Pers. and *B. cinerea* B05.10 after treatment with Z24 at 0.05 and 0.1 μg/ml, respectively. (**P**) Fold change in Bcthi4 activity in *B. cinerea* pers. and *B. cinerea* B05. (**Q** and **R**) The Bcthi4 rabbit polyclonal antibodies, G2206 and G2207, were successfully prepared. (**S**) Z24-enriched Bcthi4 protein was verified by DARTS (*n* = 3 exp.). (**T**) Thermal stability of Bcthi4 protein with or without Z24 treatment (*n* = 3 exp.).

In previous proteomics-based studies, we conducted a preliminary investigation into the mechanism of action of neocryptolepine on *R. solani* and found that it has a substantial effect on the thiamine metabolism process of *R. solani* (*31*). Therefore, neocryptolepine was selected as the lead molecule for the subsequent optimization phase, and the candidate compound Z24 was synthesized. The efficacy of Z24 against a range of plant pathogenic fungi suggests that it may have a mechanism of action distinct from that of the first-line fungicide used to treat *B. cinerea* infection. Consequently, identifying the direct targets of Z24 and further elucidating its unique mechanism of action is of substantial importance. To investigate the molecular mechanisms by which Z24 exerts antifungal bioactivity, drug affinity responsive target stability (DARTS) technology and mass spectrometry analysis were performed (Fig. 2A). Coomassie brilliant blue (Fig. 2B) and silver staining (Fig. 2C) revealed the presence of distinct protein bands (B1 and B2) in the 25- to 35-kDa range. Additionally, DARTS experiments showed that compound Z1 produced the same protein bands in the same location as compound Z24 (fig. S1). Protease treatment resulted in decreased protein content in the dimethylsulfoxide (DMSO) group and increased protein content in the Z24 group as concentration increased. A total of 367 proteins were identified as potential targets of Z24 (tables S1 and S2). These results suggest that Z24 may target multiple proteins in *B. cinerea*. Among the candidate proteins, thiazole synthase (fig. S2), which is involved in the thiamine biosynthesis pathway and has the highest abundance, was selected for further investigation. The intensity-based absolute quantification of the Bcthi4 protein was 37.11%, while that of the Bcnmt1 protein was 8.08% (Fig. 2D). Thiamine thiazole synthase, also known as Bcthi4, is the key enzyme in the thiamine biosynthesis pathway responsible for synthesizing the thiazole component. Bcnmt1 is involved in pyrimidine production, and the thiazole and pyrimidine components are coupled to form thiamine (*12*, *16*). Additionally, high-abundance proteins BCIN_16g03380 and BCIN_03g01010 were identified, suggesting that Z24 may exert antifungal activity in *B. cinerea* through a range of possible targets (Fig. 2E).

Next, we explored the possible binding mode between Z24 and Bcthi4. Because of a lack of Bcthi4 crystal structure, we used molecular docking to predict the binding site of Z24. The three-dimensional structure of Bcthi4 predicted by AlphaFold 3 (https://golgi.sandbox.google.com/) was chosen as the receptor structure. The docking result showed that the binding energy between Z24 and the Bcthi4 protein was −8.185 kcal/mol, showing a strong binding activity (Fig. 2F). The expression and purification of the Bcthi4^WT^ protein was showed in Fig. 2H and fig. S4. As indicated by surface plasmon resonance (SPR) analysis, Z24 can dose-dependently bind to immobilized Bcthi4^WT^ protein (Fig. 2I) with a high affinity [dissociation constant (*K*_d_) of 9.425 × 10^−5^] (Fig. 2J). The phenyl group forms hydrophobic interactions with the residues Met^304^ and Thr^232^. The carbon-hydrogen bond formed by residue Thr^231^ is located between these two residues and has the shortest interatomic distance, which may influence the molecular binding and interactions (Fig. 2G). To verify the predicted binding mode, we generated the mutants of Bcthi4: Met^304^, Thr^232^, and Thr^231^ (Fig. 2K and fig. S5), and retested the binding ability of Z24 by SPR. The results showed that the mutant completely abolished the binding of Z24 to Bcthi4 (Fig. 2, L and M). Bcthi4 plays an essential role in the thiamine biosynthesis pathway as a thiazole synthase (*12*). Therefore, we further investigated whether the combination of Bcthi4 and Z24 inhibits Bcthi4 activity. Compared with the control group, Z24 significantly inhibited the activity of Bcthi4 in *B. cinerea* Pers. (Fig. 2N) and *B. cinerea* B05.10 (Fig. 2O) strains when the concentrations reached 0.05 and 0.1 μg/ml. As depicted in Fig. 2P, the inhibitory effect of Z24 on Bcthi4 in *B. cinerea* B05.10 was stronger than that on Bcthi4 in *B. cinerea* Pers. To further confirm the binding of Z24 to Bcthi4, we successfully produced a rabbit polyclonal antibody against the Bcthi4 protein (Fig. 2, Q and R), which exhibited high sensitivity and specificity (fig. S3). Subsequently, the binding of Z24 to Bcthi4 was further investigated using DARTS assay and cellular thermal shift assay (CETSA). Consistent with the above results, Z24 significantly increased Bcthi4 accumulation at concentrations ranging from 250 to 500 μg/ml (compared to the DMSO solvent control) (Fig. 2S). Notably, with the addition of Z24, the stability of Bcthi4 was considerably improved, suggesting the possible formation of a Z24/Bcthi4 complex. Moreover, as shown in Fig. 2T, Z24 significantly increased Bcthi4 accumulation at temperatures ranging from 50° to 80°C (compared to the DMSO solvent control), indicating a direct interaction with Bcthi4 via thermal stability. Together, these results suggest that Bcthi4 has the potential to be an attractive antifungal target, and screening for Bcthi4 inhibitors could be an effective strategy for developing previously unidentified fungicides.

### Transcriptomic analysis of *B. cinerea* under Z24 treatment

**Fig. 3.**
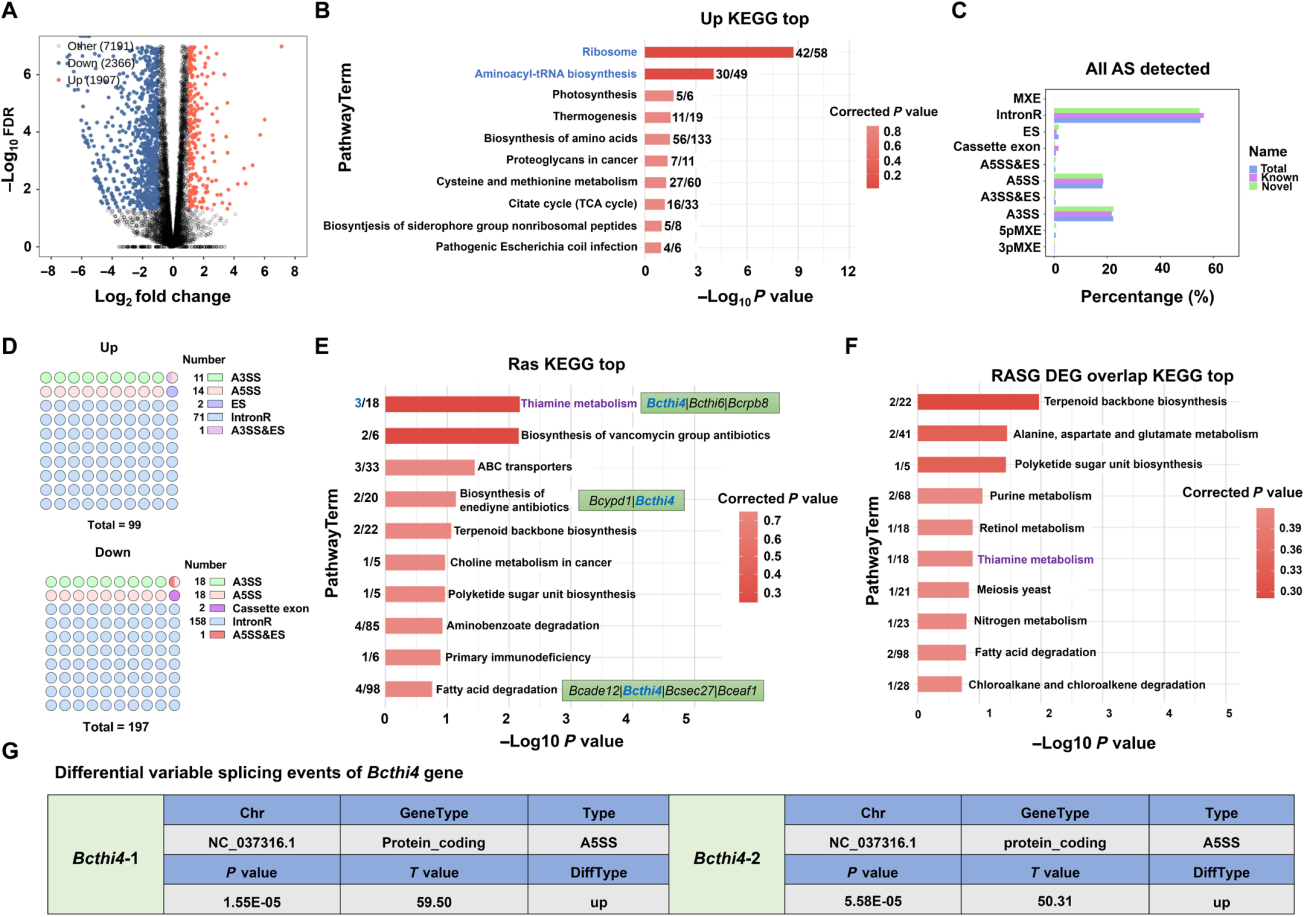
DEGs and RASG analysis. (**A**) Volcano plot analysis of differential gene expression, including 1907 up-regulated genes and 2366 down-regulated genes. FDR, false discovery rate. (**B**) Kyoto Encyclopedia of Genes and Genomes (KEGG) enrichment analysis of up-regulated differentially expressed genes (DEGs). (**C**) Overview of all detected AS events. (**D**) Types and numbers of AS events detected in up-regulated and down-regulated AS genes. (**E**) KEGG enrichment analysis of variable AS genes (RASGs). (**F**) KEGG enrichment analysis of the overlap between RASGs and DEGs. (**G**) Differential variable splicing events of the *Bcthi4* gene.

To investigate the mechanism of action of Z24 in *B. cinerea* at the genetic level, we performed transcriptomic sequencing analysis. As shown in Fig. 3A, 273 differentially expressed genes (DEGs) were identified in *B. cinerea* after treatment with Z24, with 1907 up-regulated and 2366 down-regulated. The top 10 enriched Kyoto Encyclopedia of Genes and Genomes (KEGG) database pathways were up-regulated, including ribosome (42 DEGs), aminoacyl-tRNA biosynthesis (30 DEGs), amino acid biosynthesis (56 DEGs), cysteine and methionine metabolism (27 DEGs), and citric acid cycle (16 DEGs). The DEGs were predominantly associated with ribosome function, aminoacyl-tRNA synthesis, and amino acid biosynthesis (Fig. 3B). Thiamine is an essential coenzyme in cell metabolism, playing a crucial role in various biochemical processes, including sugar metabolism and energy production. When thiamine synthesis is inhibited, cells may enhance protein synthesize by up-regulating ribosomal gene expression to compensate for the metabolic stress caused by thiamine deficiency. Moreover, in fungi, a significant increase in the expression of aminoacyl-tRNA synthetase genes may occur as a biological response to thiamine deficiency, aimed at improving protein synthesis efficiency and thus supporting cell function and growth. In fungi, the TPP riboswitch regulates gene expression through alternative splicing (AS) of the pre-mRNA (*32*). THI4 riboswitches, found in gene introns, may contribute to AS, as demonstrated in *N. crassa* (*24*). Meanwhile, the role of AS in eukaryotic gene regulation is becoming increasingly clear (*33*, *34*). AS is a crucial posttranscriptional regulatory mechanism that enhances transcriptome diversity and protein complexity. However, few studies have focused on AS in fungi. Further analysis of AS data revealed a total of 70,420 splicing sites in the RNA sequencing (RNA-seq) data, with 47,107 being previously unknown splicing sites and 23,313 being known splicing sites. A total of 4290 variable splicing events were identified, with 889 known (annotated) and 3401 unannotated variable splicing events, which account for 79.28% of all observed AS events. Common types of splicing events included alternative 3′ splice site (A3SS), A5SS, and intron recognition (IntronR), as shown in Fig. 3C. The types and quantities of up-regulated and down-regulated AS events are presented in Fig. 3D. For up-regulated events, these include A3SS (11), A3SS and exon skipping (A5SS&ES) (1), A5SS (14), ES (2), and IntronR (71), while, for down-regulated events, the types and numbers are A3SS (18), A5SS (18), A5SS&ES (1), IntronR (158), and cassette exon (2). KEGG pathway enrichment analysis of differentially variable splicing genes revealed that the thiamine metabolic pathway showed the most notable enrichment. Three differentially variable splicing genes were enriched in this pathway, including the target gene *Bcthi4*, as well as *Bcthi6* and *Bcrpb8* (Fig. 3E). As shown in Fig. 3F, the thiamine metabolic pathway was also notably enriched in overlap analysis of differentially variable splicing genes and DEGs. Notably, a significantly up-regulated A5SS splicing event was detected in the *Bcthi4* riboswitch of *B. cinerea*, suggesting that Z24 treatment induced selective splicing of the *Bcthi4* precursor mRNA in *B. cinerea* and increased its splicing (Fig. 3G). This indicates that, similar to plants, fungi may use AS to increase proteome complexity and respond to transcriptional changes induced by stress, thereby reducing the metabolic cost of processing all AS transcripts (*35*).

### High *Bcthi4* expression is correlated with *B. cinerea* survival under Z24 stress

**Fig. 4.**
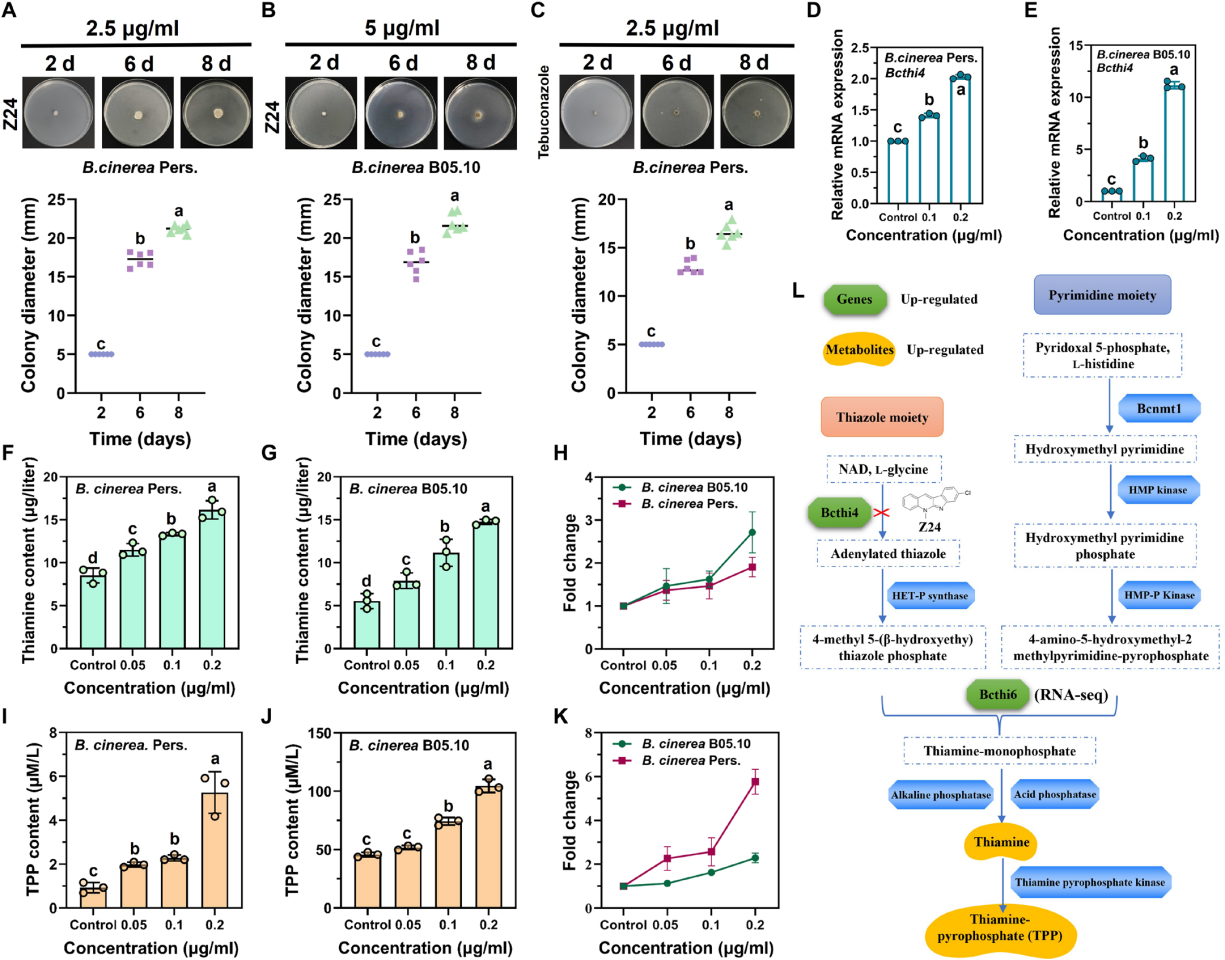
Available thiamine promotes the growth of *B. cinerea* under Z24 stress by up-regulating genes and metabolites. (**A** and **B**) Colony diameter measurement of *B. cinerea* Pers. and *B. cinerea* B05.10 following treatment with Z24 (2.5 and 5 μg/ml). (**C**) Colony diameter of *B. cinerea* Pers. after treatment with tebuconazole (2.5 μg/ml). d, days. (**D** and **E**) Reverse transcription quantitative polymerase chain reaction (RT-qPCR) analysis of *Bcthi4* gene expression in *B. cinerea* Pers. and *B. cinerea* B05.10 after Z24 treatment. (**F** to **H**) Measurement of thiamine content in (F) *B. cinerea* Pers. and (G) *B. cinerea* B05.10, as well as the (H) fold change after Z24 treatment. (**I** to **K**) Measurement of TPP content in (I) *B. cinerea* Pers. and (J) *B. cinerea* B05.10, along with the (K) fold change after Z24 treatment. (**L**) Z24 specifically inhibits the growth of *B. cinerea* by targeting Bcthi4 in the thiamine biosynthesis pathway. HET-P, 4-methyl-5-(2-phosphooxyethyl)thiazole; NAD, nitric acid dihydrate.

When exposed to external stress, plant pathogenic fungi exhibit a certain degree of tolerance and can withstand damage by activating their defense mechanisms. These processes may involve modifications to metabolic pathways, the synthesis of protective chemicals, and other adaptive responses. For example, the transcription of drug target genes (FgCYP51s) was significantly induced in the wild-type *Fusarium graminearum* after treatment with the azole compound tebuconazole, but not with iprodione or fludioxonil, which act via different mechanisms (*36*). Therefore, we further investigated the effects of Z24 on the genes and metabolism of *B. cinerea*. Phenotypic characterization revealed that Z24 and the tebuconazole treatment groups showed increased mycelial growth on PDA plates on days 6 and 8, respectively (Fig. 4, A to C). We hypothesized that, during this process, the target genes and related metabolites of *B. cinerea* affected by Z24 would also change notably. Expectedly, genes involved in thiamine metabolism are tightly regulated by thiamine levels (*37*). A study showed that the green alga *Chlamydomonas reinhardtii* uses TPP riboswitches to down-regulate the expression of *THIC* and *THI4* when exposed to exogenous thiamine (*38*).

To investigate whether thiamine biosynthesis is inhibited by compound Z24, we examined the transcription of the *Bcthi4* gene, which encodes a key enzyme in thiamine biosynthesis. Reverse transcription quantitative polymerase chain reaction (RT-qPCR) assays showed that the expression of *Bcthi4* was significantly up-regulated in *B. cinerea* Pers. (Fig. 4D) and *B. cinerea* B05.10 (Fig. 4E) under Z24 stress. Notably, as a typical suicide enzyme, the transcriptional level of *Bcthi4* was markedly up-regulated following Z24 treatment, likely as a compensatory response to thiamine deficiency in the cytoplasm. These findings indicate that Z24 inhibits the thiamine biosynthesis pathway in *B. cinerea*. The utilization of energy during fungal growth and metabolism, as well as the synthesis of various substances required for cell growth, is highly efficient, especially for thiamine biosynthesis, which is an energy-intensive pathway. This may also explain the presence of feedback regulatory mechanisms in species with complete biosynthesis pathways (*38*). To further investigate the effect of Z24 on the thiamine pathway mediated by Bcthi4, we compared thiamine and TPP content related to this pathway in *B. cinerea* Pers. and *B. cinerea* B05.10 strains. As shown in Fig. 4F, thiamine content in *B. cinerea* Pers. mycelial cells increased by 1.4, 1.5, and 1.9 times in a concentration-dependent manner under Z24 stress (Fig. 4H). In *B. cinerea* B05.10 mycelial cells, the thiamine content increased by 1.4, 1.6, and 2.7 times (Fig. 4H) in a concentration-dependent manner (Fig. 4G). Thiazole synthase is a key enzyme in the thiamine biosynthesis pathway, and changes in its expression level may affect thiamine synthesis. These results suggest that, under conditions of limited thiamine synthesis, fungi may up-regulate the expression of enzymes such as thiazole synthase to increase the production of thiamine precursors, thereby partially compensating for the thiamine deficiency. TPP, the active form of thiamine, was also quantified. The experimental results revealed that the TPP content in *B. cinerea* Pers. (Fig. 4I) and *B. cinerea* B05.10 (Fig. 4J) mycelial cells significantly increased with increasing Z24 concentrations (Fig. 4K). A previous study showed that the expression of the *Bcthi6* gene is negatively regulated by intracellular TPP, suggesting that fungi can regulate thiamine biosynthesis through a feedback mechanism (*39*). According to the transcriptome data, the expression of the *Bcthi6* gene in *B. cinerea* was also significantly up-regulated after Z24 treatment, further indicating that the thiamine biosynthesis process was inhibited.

Collectively, the thiamine biosynthetic pathway is tightly regulated to ensure that TPP production meets cellular demands. This is typically achieved by controlling the expression of one or more enzyme genes. For instance, exogenous thiamine notably inhibits the transcription of thiamine-related genes in bacteria and fungi (*40*). In contrast, under thiamine deficiency, the expression of the thiamine synthase gene is markedly up-regulated. Together, the data indicate that compound Z24 specifically inhibits the thiamine biosynthesis pathway (Fig. 4L).

### Pathway validation of Z24-induced disturbance in thiamine metabolism

**Fig. 5.**
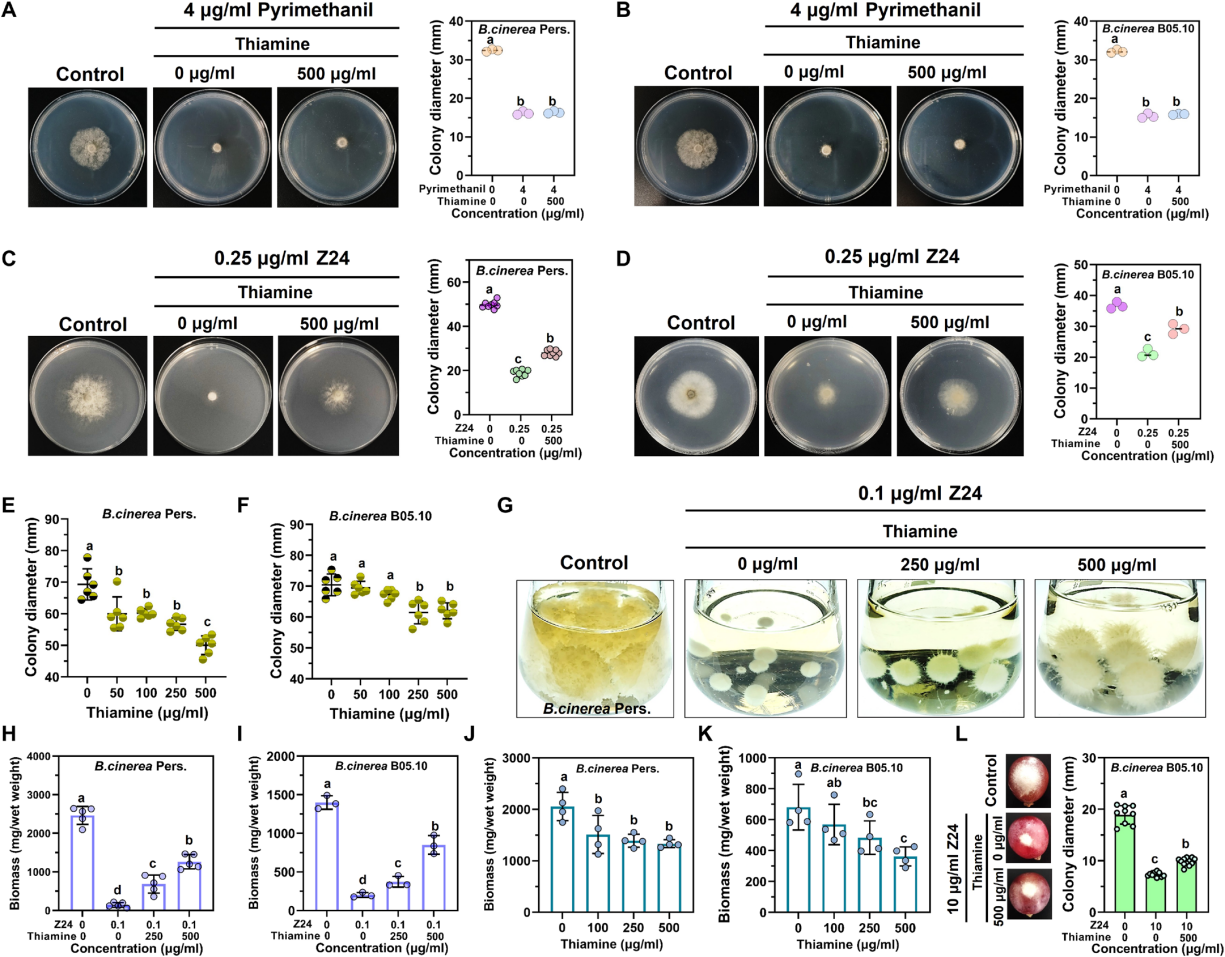
Metabolic pathway blockade identifies fungicide Z24. (**A** and **B**) The effect of exogenous thiamine (500 μg/ml) on the growth of *B. cinerea* Pers. and *B. cinerea* B05.10 after treatment with pyrimethanil (4 μg/ml) on PDA plates. (**C** and **D**) The growth inhibitory activity of *B. cinerea* Pers. and *B. cinerea* B05.10 in the presence of thiamine (500 μg/ml) after treatment with Z24 (0.25 μg/ml) on PDA plates. (**E** and **F**) The inhibitory activity of thiamine (at 50, 100, 250, 500 μg/ml) against *B. cinerea* Pers. and *B. cinerea* B05.10 on PDA plates. (**G** and **H**) The effect of exogenous thiamine (at 250 and 500 μg/ml) on the growth of *B. cinerea* Pers. after treatment with Z24 in liquid PDA medium. (**I**) The growth inhibitory activity of *B. cinerea* B05.10 in the presence of thiamine (250 and 500 μg/ml) after treatment with Z24 (0.1 μg/ml) in liquid PDA medium. (**J** and **K**) Inhibitory activity of thiamine (at 100, 250, and 500 μg/ml) against *B. cinerea* Pers. and *B. cinerea* B05.10 in liquid PDA medium. (**L**) The growth inhibitory activity of *B. cinerea* B05.10 in the presence of thiamine (500 μg/ml) after treatment with Z24 (10 μg/ml) on grapes.

To further verify the mechanism of action of Z24, exogenous thiamine (250 and 500 μg/ml) was supplemented to support *B. cinerea* growth and assess the inhibitory effects of Z24 and pyrimethanil, both alone and in combination. Both *B. cinerea* Pers. and *B. cinerea* B05.10 strains exhibited severe growth defects after pyrimethanil treatment. Further validation with exogenous thiamine supplementation (500 μg/ml) showed that both *B. cinerea* Pers. and *B. cinerea* B05.10 strains displayed similar inhibitory activity, regardless of the presence or absence of thiamine (Fig. 5, A and B). Pyrimethanil is known to inhibit the production of infection-causing enzymes, rather than disrupting the thiamine metabolic pathway, to prevent fungal infections and kill fungi. In contrast, *B. cinerea* Pers. and *B. cinerea* B05.10 strains exhibited significantly reduced growth inhibition when grown in the presence of thiamine after Z24 treatment (Fig. 5, C and D). We also investigated whether thiamine could enhance *B. cinerea* growth in PDA medium. Thiamine did not significantly enhance fungal growth, and high concentrations of thiamine (500 μg/ml) actually inhibited *B. cinerea* growth (Fig. 5, E and F). Similarly, both *B. cinerea* Pers. and *B. cinerea* B05.10 strains showed significantly reduced concentration-dependent growth inhibition when grown in the presence of thiamine after treatment with Z24 (0.1 μg/ml) in liquid PDA medium (Fig. 5, G to I). Meanwhile, thiamine also exhibited significant inhibitory activity against *B. cinerea* in liquid PDA medium (Fig. 5, J and K). Last, *B. cinerea* showed significantly reduced growth inhibition when cultivated with thiamine (500 μg/ml) after treatment with Z24 (10 μg/ml) on grapes (Fig. 5L). Together, these results strongly suggest that thiamine is crucial for *B. cinerea* growth, and Z24 likely interferes with the *B. cinerea* thiamine biosynthesis pathway.

### Metabolite analysis of thiamine metabolism disruption by Z24

**Fig. 6.**
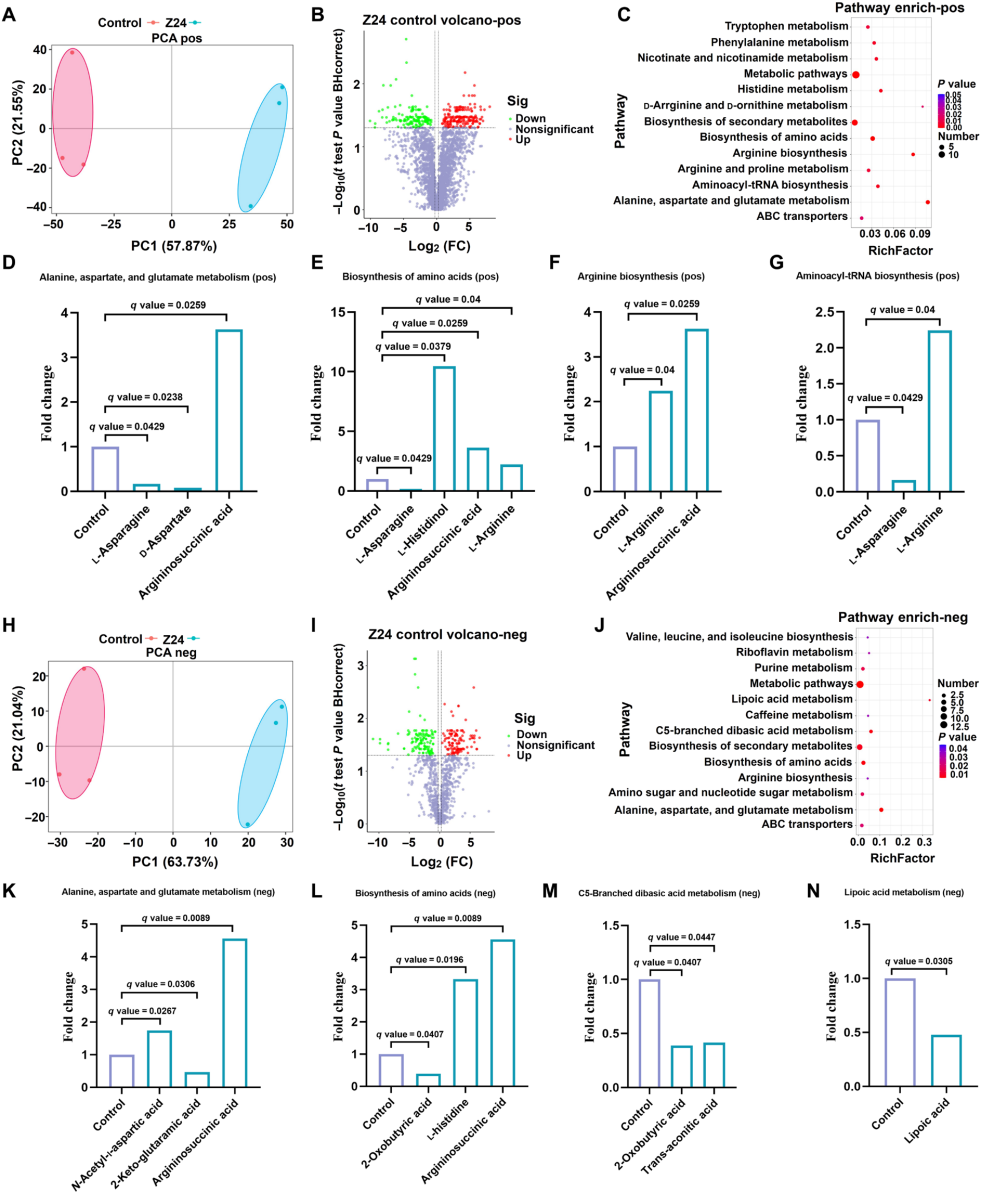
The relative content of up-regulated and down-regulated metabolites shows a substantial correlation with amino acid metabolism in *B. cinerea.* (**A**) Principal components analysis (PCA) score plots in electrospray ionization (ESI)^+^ mode. (**B**) Volcano plot analysis of differential metabolomics in ESI^+^ mode. FC, fold change. (**C**) Pathway enrichment analysis of differential metabolomics in ESI^+^ mode. (**D** to **G**) The fold change of metabolites in (D) alanine, aspartate, and glutamate metabolism; (E) biosynthesis of amino acids; (F) arginine biosynthesis; and (G) aminoacyl-tRNA biosynthesis in ESI^+^ mode. (**H**) PCA score plots in ESI^−^ mode. (**I**) Volcano plot analysis of differential metabolites in ESI^−^ mode. (**J**) Pathway enrichment analysis of differential metabolites in ESI^−^ mode. (**K** to **N**) The fold change of metabolites in (K) alanine, aspartate and glutamate metabolism; (L) biosynthesis of amino acids; (M) C5-branched dibasic acid metabolism; and (N) lipoic acid metabolism in ESI^−^ mode. The *q* value is obtained by correcting the *P* value using the FDR. BHcorrect, Benjamini-Hochberg correction.

Metabolite analysis was conducted to gain a comprehensive understanding of the antifungal mechanism of Z24 and to verify its impact on the thiamine metabolism pathway. TPP, the active form of thiamine, is an essential cofactor in glycolysis, the tricarboxylic acid cycle, the pentose phosphate pathway, and the synthesis of branched-chain amino acids. It plays a critical role in the carboxylation and decarboxylation of various metabolic intermediates (*41*, *42*). To analyze the metabolites of *B. cinerea* after treatment with Z24, we performed liquid chromatography–tandem mass spectrometry. As illustrated in fig. S6A, metabolites identified in the positive ion model and annotated to biological processes include amino acids, carbohydrates, coenzymes, vitamins, and others. Figure S6B shows the primarily enriched pathways, including transmembrane transport, translation, amino acid metabolism, coenzyme and vitamin metabolism, carbohydrate metabolism, lipid metabolism, and others. Principal components analysis (PCA) revealed a clear distinction between the control and Z24 treatment groups, with the contribution rates of PC1 and PC2 being 57.87 and 21.55%, respectively (Fig. 6A). As a result, 331 differential metabolites were identified in positive ion mode, including 184 up-regulated and 147 down-regulated metabolites (Fig. 6B). KEGG pathway analysis revealed that Z24 primarily affected amino acid biosynthesis, including alanine, aspartate, and glutamate metabolism; tryptophan metabolism; phenylalanine metabolism; niacin and niacinamide metabolism; histidine metabolism; arginine and proline metabolism; aminoacyl-tRNA biosynthesis; and adenosine 5′-triphosphate (ATP)–binding cassette (ABC) transporter in *B. cinerea* (Fig. 6C). The abundance of l-histidinol, argininosucinic acid, and l-arginine was significantly up-regulated, while d-aspartate and l-asparagine were significantly down-regulated in the amino acid metabolism and aminoacyl-tRNA biosynthesis pathways (Fig. 6, D to G).

Carbohydrates and amino acids, which are also involved in biological processes, were classified as metabolites in the negative ion mode, as shown in fig. S7A. The enrichment pathways included lipid metabolism, coenzyme and vitamin metabolism, carbohydrates metabolism, and amino acids metabolism (fig. S7B). PCA revealed that PC1 contributed 63.73% and PC2 contributed 21.04%, as shown in Fig. 6H. A total of 247 different metabolites were identified in the Z24 group in negative ion mode, with 119 up-regulated and 128 down-regulated, compared to those in the control group (Fig. 6I). KEGG pathways analysis revealed that Z24 primarily affected amino acid biosynthesis; alanine, aspartate, glutamate metabolism; and ABC transporter in *B. cinerea* (Fig. 6J). The abundance of l-histidinol, argininosucinic acid, and *N*-acetyl-l-aspartic acid was significantly up-regulated, while 2-oxobutyric acid, trans-aconitic acid, lipoic acid, and 2-keto-glutaramic acid were significantly down-regulated in amino acid metabolism, C5-branched dibasic acid metabolism, and lipoic acid metabolism (Fig. 6, K to N). TPP-dependent enzymes, such as branched-chain α-ketoacid dehydrogenase (BCKDC) and acetolactate synthase (AHAS), play a crucial role in promoting cell growth and preventing metabolic stress (*43*). AHAS and BCKDC are responsible for the synthesis and degradation of branched-chain amino acids, respectively (*44*). These findings support the notion that Z24 notably affects TPP-mediated microbial metabolism activities and play a key role in the metabolic profile of *B. cinerea*, primarily interfering with amino acid metabolism in fungi.

### Representative compound Z24 demonstrates favorable safety

**Fig. 7.**
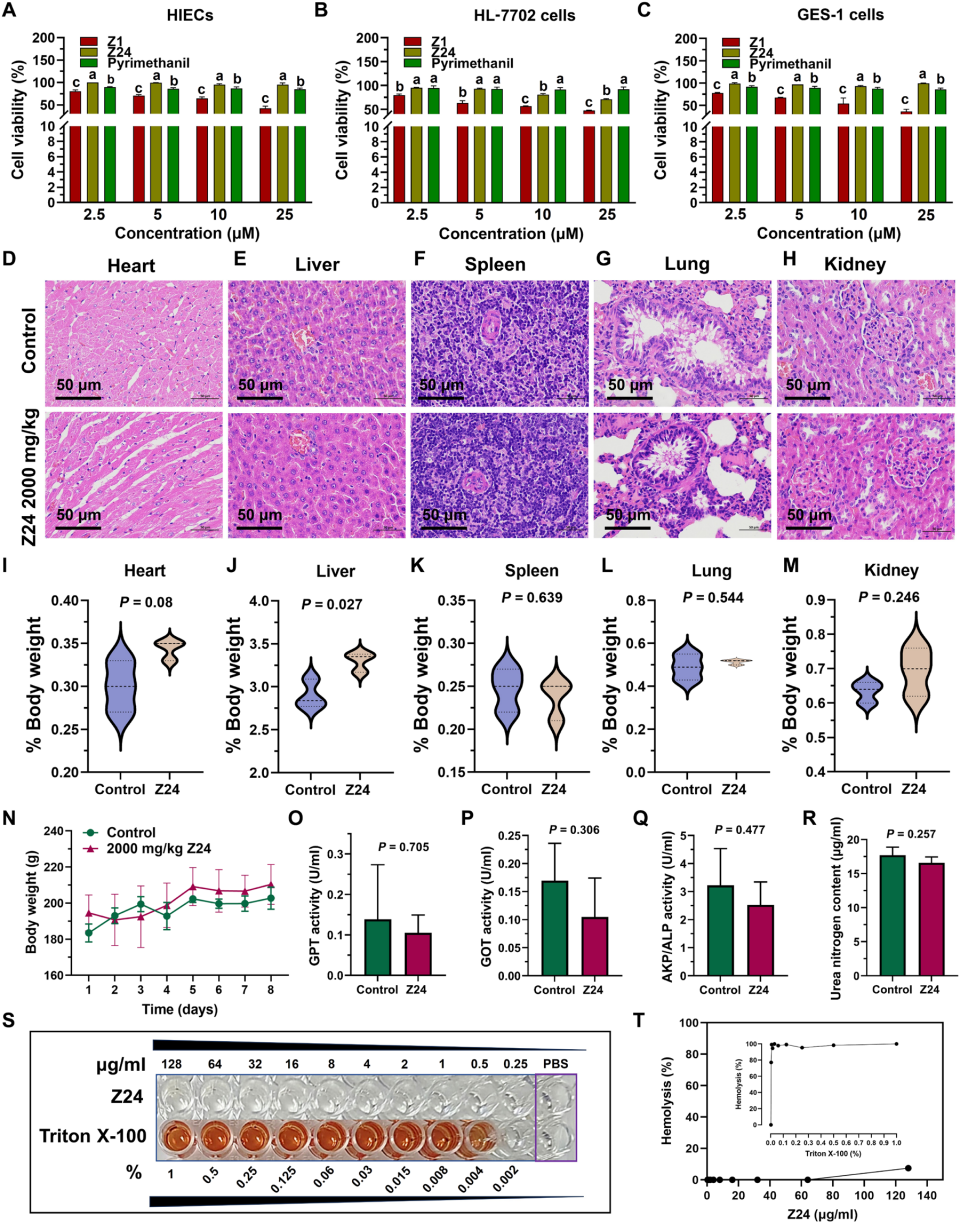
In vitro and in vivo toxicity evaluation of compound Z24. (**A** to **C**) Inhibitory activity of Z24 on (A) HIECs, (B) HL-7702 cells, and (C) GES-1 cells. HIECs, HL-7702 cells, and GES-1 cells were treated with Z24, Z1, and pyrimethanil for 48 hours, and cell viability was assessed using the 3-(4,5-dimethylthiazol-2-yl)-2,5-diphenyltetrazolium bromide (MTT) assay. (**D** to **H**) Effects of Z24 (2000 mg/kg) on pathological changes in the (D) heart, (E) liver, (F) spleen, (G) lung, and (H) kidney tissues of Wistar rats. (**I** to **M**) Relative body weights of the (I) heart, (J) liver, (K) spleen, (L) lung, and (M) kidney in the control and Z24 (2000 mg/kg) groups. (**N**) Body weight changes in Wistar rats after Z24 administration. (**O** to **R**) Changes in blood biochemical markers: (O) GPT, (P) GOT, (Q) AKP/ALP, and (R) urea nitrogen in rats from the control and Z24 groups. (**S** and **T**) Representative images and quantitative statistical data for the Z24 and Triton X-100 hemolysis rates (*n* = 3).

The effects of compounds Z1, Z24, and pyrimethanil on cell viability were assessed using the 3-(4,5-dimethylthiazol-2-yl)-2,5-diphenyltetrazolium bromide (MTT) assay in normal human intestinal epithelial cells (HIECs), normal human hepatocytes (HL-7702), and gastric epithelial cells (GES-1). Cell viability was measured after exposure to different concentrations of these compounds. As shown in Fig. 7 (A to C), compound Z24 exhibited lower cytotoxicity, with cell viability ranging from 71 to 95%, compared to Z1 (36.5 to 80.5%) and pyrimethanil (84.75 to 94%). Compound Z24 demonstrated the lowest cytotoxicity, making it a promising candidate for further research.

Compound Z24 was also evaluated for acute oral toxicity in Wistar rats following the Organization for Economic Co-operation and Development (OECD) Guideline 423. The medium lethal dose of Z24 was found to be greater than 2000 mg/kg after 14 days of close observation, indicating that Z24 has an acceptable safety profile when administered orally. Histological examination of tissue sections from the heart, liver, spleen, lungs, and kidneys revealed no signs of inflammation, and the organ architecture appeared normal. Gross necropsy of the female rat revealed no lesions or abnormalities in the organs. Microscopical analysis of cardiac sections showed no changes in the cardiomyocyte architecture. There were no indications of cardiac myopathy, myofiber degeneration, necrosis, vacuolation, or mononuclear cell infiltration, leading to the conclusion that Z24 therapy is safe for the rat heart (Fig. 7D). Z24 also appears to be safe for normal liver function, as histological examination of rat hepatic sections treated with Z24 revealed no signs of periportal hepatocellular vacuolation, lipid accumulation, localized inflammation, focal tension, hepato-diaphragmatic nodules, or inflammatory cell infiltration (Fig. 7E). Additionally, the presence of immune cells in the lymphoid tissue and blood vessels within the connective tissue trabeculae extending from the hilus into the lymph parenchyma indicated that Z24 had no effect on the rat spleen (Fig. 7F). The absence of invasive lymphocytes, plasma cells, and histocytes in the subpleural regions of rat lung sections treated with Z24 indicates the absence of inflammation (Fig. 7G). Rats treated with Z24 displayed delicate and thin glomerular capillary loops, with a normal number of mesangial cells and endothelium, suggesting normal glomerular filtration (Fig. 7H). The heart, kidney, spleen, and lung remained relatively unchanged, with the exception of the liver (Fig. 7, I to M). Furthermore, no significant body weight loss was observed after the various treatments, as shown in Fig. 7N.

Subsequently, the biochemical indicators of the liver and kidney functions were assessed using blood samples taken from the rats mentioned above. As shown in Fig. 7 (O to R), compound Z24 did not induce any significant changes in glutamic pyruvic transaminase (GPT), glutamic oxalacetic transaminase (GOT), alkaline phosphatase (AKP/ALP), or urea nitrogen levels, suggesting that Z24 has no noticeable negative impact on liver or kidney function. Figure 7 (S and T) presents the results from the in vitro hemolysis tests. No visible hemolysis was observed after co-incubating compound Z24 with sheep erythrocyte suspensions. Z24 exhibited no hemolysis at a concentration of 64 μg/ml (114 times the EC_50_), indicating that the compound Z24 has negligible hemolytic activity on human red blood cells.

### Compound Z24 exhibits promising in vivo efficacy against *B. cinerea*

**Fig. 8.**
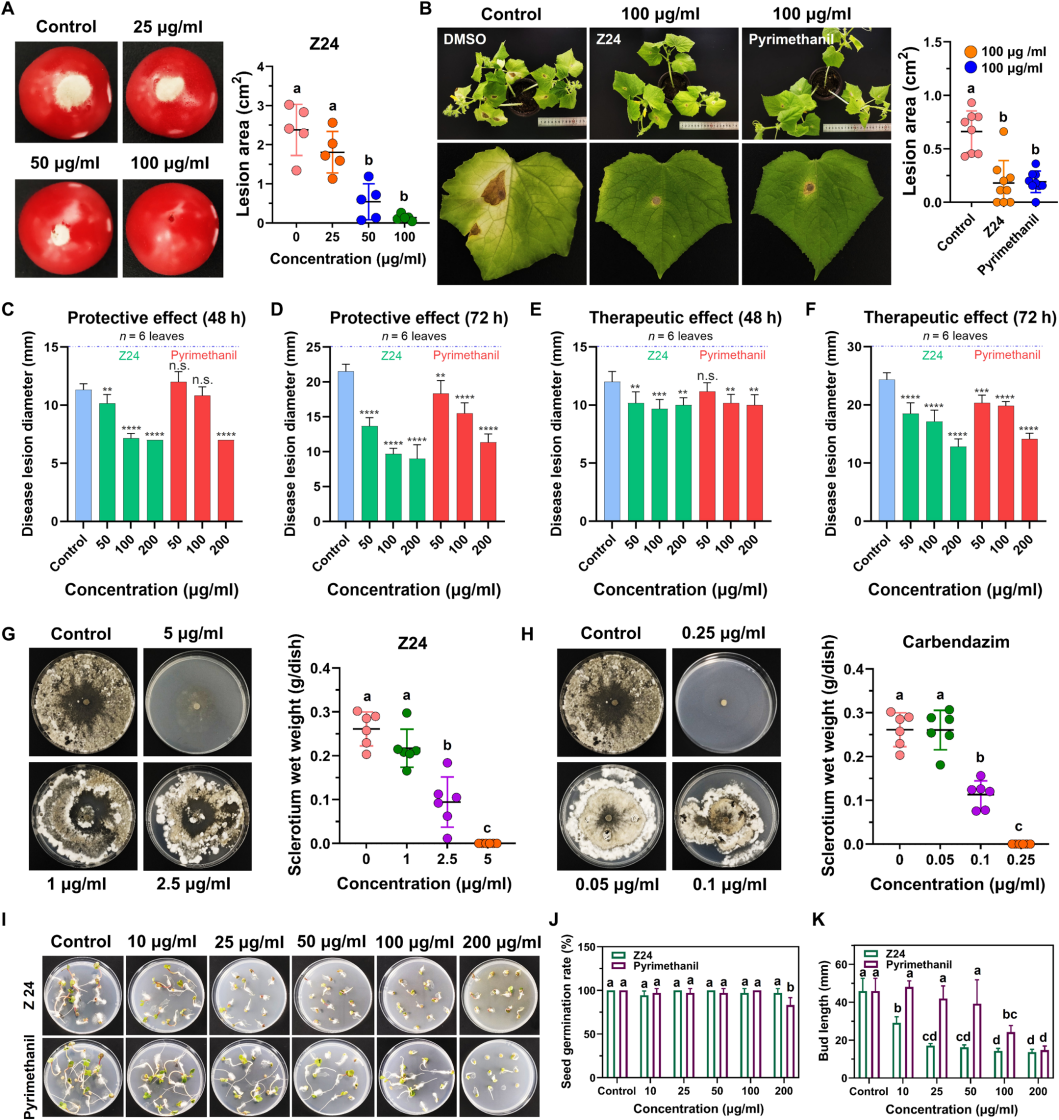
Phytotoxicity and plant protection or therapy by Z24 in tomato and cucumber. (**A**) Gray mold disease symptoms in tomatoes after treatment with various concentrations of Z24 (*n* = 5). (**B**) Gray mold disease symptoms on cucumber leaves; data are presented as means ± SD, *n* = 7 to 10 biologically independent samples. (**C** and **D**) Protective effects of Z24 and pyrimethanil on cucumber leaves after infection with *B. cinerea* Pers. plugs. (**E** and **F**) Therapeutic effects of Z24 and pyrimethanil on cucumber leaves after infection with *B. cinerea* Pers. plugs. h, hours. (**G** and **H**) Inhibitory activity of Z24 and carbendazim (a positive control against *S. sclerotiorum* strain) on sclerotia formation (*n* = 6). (**I**) Phytotoxicity of Z24 on radish seed (*n* = 12). (**J**) Effect of Z24 on the germination rate of radish seeds. (**K**) Effect of Z24 on the bud length of radish seeds. Note that control contains 0.25% DMSO. **P < 0.001, ***P = 0.0001, and ****P < 0.0001. n.s., not significant.

Compound Z24 was evaluated for its in vivo antifungal activity using tomato and cucumber pot models. Several opportunistic pathogenic fungi produce a large number of airborne spores. Therefore, we sprayed various concentrations of Z24 onto tomatoes, incubated the tomatoes for 72 hours after inoculating them with *B. cinerea* Pers. spores, and quantified the disease symptoms. In the control group, *B. cinerea* Pers. spores formed colonies on the tomatoes after 72 hours, causing gray mold, a characteristic symptom of tomato gray mold disease. Symptom development was inhibited when tomatoes were pretreated with Z24, and the protective effect was concentration dependent (Fig. 8A). Z24 almost completely suppressed infection by *B. cinerea* Pers. spores and provided full protection against gray mold disease at a concentration of 100 μg/ml. Therefore, we sprayed an equal concentration (100 μg/ml) of Z24 and pyrimethanil onto cucumber leaves, incubated the treated cucumber leaves for 7 days after applying *B. cinerea* Pers. spores, and quantified the disease symptoms. In the control experiments, *B. cinerea* Pers. spores formed colonies on cucumber leaves after 7 days, causing gray mold on the leaves. Symptom development was inhibited when cucumber leaves were pretreated with Z24 and pyrimethanil. In contrast, Z24 inhibited symptom development in cucumber leaves at 100 μg/ml as effectively as pyrimethanil (Fig. 8B).

Furthermore, the mycelium formed by *B. cinerea* spore germination contacts the plant epidermis, penetrates the cuticle, and damages the plant tissues by releasing numerous degrading enzymes and toxins. We further evaluated the protective and therapeutic effects of Z24 on mycelia-infected cucumber leaves. Infection with *B. cinerea* Pers. plugs led to the development of gray mold lesions after 48 and 72 hours of incubation. Symptom development was significantly inhibited when cucumber leaves were pretreated with Z24 (50, 100, and 200 μg/ml) and pyrimethanil, and this protective effect was concentration dependent. In contrast, Z24 exhibited a more effective protective effect than the positive control, pyrimethanil (Fig. 8, C and D). As shown in Fig. 8 (E and F), Z24 also demonstrated a more potent therapeutic effect than pyrimethanil. Additionally, Z24 completely inhibited sclerotia formation in the tested *S. sclerotiorum* strain at a dose of 5 μg/ml (Fig. 8G), whereas carbendazim, a positive control, effectively inhibited sclerotium formation at 0.25 μg/ml (Fig. 8H). Subsequently, *Raphanus raphanistrum* seeds were grown in Murashige and Skoog agar medium treated with Z24 (0 to 200 μg/ml) and pyrimethanil to assess their phytotoxicity on seed germination and bud tube growth (Fig. 8I). As shown in Fig. 8J, Z24 had no effect on seed germination at concentrations of 0 to 200 μg/ml. However, at concentrations of 10 to 200 μg/ml, it significantly delayed *R. raphanistrum* tube growth (Fig. 8K). Pyrimethanil did not affect seed germination at concentrations of 0 to 100 μg/ml (Fig. 8J) but significantly slowed *R. raphanistrum* tube growth at 100 to 200 μg/ml (Fig. 8K). Collectively, compound Z24, with its safety and efficiency, exhibits antifungal activity in crops and is a promising fungicide for agriculture use.

## DISCUSSION

Globally, the prevalence of *B. cinerea* infection remains notable. Given the increasing prevalence of drug-resistant *B. cinerea* strains, it is crucial to develop previously unidentified therapeutic approaches that overcome the limitations of existing treatments. Using antifungal agents with previously unknown mechanisms of action against *B. cinerea* is essential to prevent cross-resistance with current fungicides.

In this study, a chemical modification was successfully developed to synthesize previously unidentified antifungal agents, and a candidate Z24, specifically targeting *B. cinerea*, was synthesized (*7*). Because of its efficacy against plant pathogenic fungi such as *B. cinerea*, *S. sclerotiorum*, *F. graminearum*, *R. solani*, *P. capsica*, and *F. oxysporum*, along with its favorable safety profile and unique mechanism of action observed in this study, Z24 may offer a potential alternative to current pesticide to address the growing issue of pesticide resistance. According to the China Pesticide Information Network, fungicides such as dicarboximides, anilinopyrimidines, phenylpyrroles, methyl-benzimidazole carbamates, quinone outside inhibitors, and SDHIs have been registered to combat gray mold. However, the environmental pressure caused by the widespread use of fungicides has driven the emergence of resistance to all major fungicides (*45*), and resistance to several fungicides has been frequently observed in *B. cinerea* across different nations (*46*, *47*). Meanwhile, broad-spectrum agricultural fungicides, such as difenoconazole, propiconazole, and tebuconazole, not only are chemically similar to first-line medical triazoles (itraconazole, posaconazole, and voriconazole) but also are also being used in increasing quantities worldwide (*48*). These findings serve as a “smoking gun,” linking the use of agricultural fungicides to clinical resistance (*49*). Mechanistically, antifungal resistance is typically caused by modifications that either directly or indirectly affect the drug-target interaction. Therefore, it is urgent to identify key pathogenic or growth regulatory factors in *B. cinerea* as previously unknown therapeutic targets and to develop previously unidentified fungicides for its management.

DARTS is a proteomics approach that has been widely adopted and applied in various biological research domains due to its unique characteristics. It facilitates the identification of potential treatment targets by assessing the active proteome in plant pathogenic fungi-relevant tissues or cells. These validated targets could be critical for understanding the key participants in disease pathways and developing targeted therapies. Several verifications were conducted to ensure the reliability of Z24-based target exploration. Additionally, molecular docking predicted the identical interactions between Z24 and the specific binding sites of Bcthi4, further confirming the validity of the target exploration results. Additionally, Z24 dose-dependently inhibited the activity of the thiazole synthase Bcthi4 in two strains of *B. cinerea*. As a result, Z24 could be considered a suitable tool for DARTS-based direct target fishing. The verification results strongly supported the mechanisms proposed in this study, as well as those observed in morphological analysis and transcriptome investigation (including variable splicing).

Bcthi4, the validated target of compound Z24, and Bcnmt1, an indispensable synthetase involved in thiazole and pyrimidine synthesis in thiamine biosynthesis pathway, are essential for fungal viability, making them promising targets for antifungal therapy. The relative abundance of thiazole synthase Bcthi4 in the identified list was 37.11%, while pyrimidine synthase Bcnmt1 accounted for 8.08%. Bcthi4 and Bcnmt1 catalyze the two metabolic branches of thiazole and pyrimidine synthesis in the fungal TPP biosynthesis pathway, which are regulated by ribose switches (*10*, *50*). Now, limited studies have been conducted on inhibitors targeting the *B. cinerea* Bcthi4 and Bcnmt1 systems. Compound Z24 was predicted and found to act on both systems, potentially inhibiting the entire TPP biosynthesis pathway. As a result, fungi would experience a TPP deficit because of the suppression of Bcthi4. To confirm that Z24 induces a TPP deficit in *B. cinerea* by targeting Bcthi4 and Bcnmt1, thiamine and TPP levels were measured in both the untreated control group and the Z24-treated group. As shown in Fig. 4 (F to K), a significant increase in thiamine and TPP levels was observed in the Z24-treated group during Z24-induced stress growth, further confirming the effect of Z24 on thiazole synthase and pyrimidine synthase in the TPP biosynthesis. As a result, TPP deficiency disrupts other TPP-related metabolic processes. The absence of TPP, particularly in synthase-related proteins (Bcthi4 and Bcnmt1), could impair the coenzyme function of *B. cinerea* in various metabolic pathways. This effect was observed in *B. cinerea* after treatment with Z24.

Thiamine is an essential cofactor in all organisms, from microorganisms to mammals, because its active form, TPP, is required by several enzymes involved in key cellular metabolic pathways, including amino acid and central carbon metabolism, branched-chain amino acid metabolism, and lipid metabolism (*12*, *17*, *51*–*54*). It also plays a critical role in numerous cellular processes in the cytoplasm, mitochondria, and peroxidase (*55*). Thiamine enters cells, is phosphorylated to form TPP, and the resulting coenzyme acts as a ligand for riboswitch-mediated regulation of RNA splicing in fungi (*23*). Numerous studies have shown that TPP directly bind to aptamers, inducing conformational changes that interfere with the expression of thiamine biosynthesis genes (*56*–*59*). In eukaryotes, only TPP riboswitches have been found (*60*, *61*), which regulate gene expression through AS (*24*, *25*). However, riboswitches have not been found in mammals (*62*). A previous study on *Aspergillus oryzae* thiA mRNA, which carries a TPP aptamer, found that thiamine (vitamin B1) supplementation in medium inhibits gene expression and that deletion of riboswitch aptamer regions disrupts thiamine responsiveness (*30*). In another study, the roles of three *N. crassa* TPP aptamers were investigated. *NMT1* and *CyPBP37* (a homolog of *THI4*, hereafter referred to as *THI4*) are two of these genes recognized as involved in thiamine metabolism. The third gene, *NCU01977.1*, encodes a protein with no known function (*63*–*65*). The findings show that thiamine induces AS of *NMT1* and *THI4* precursor mRNAs in *N. crassa*, as well as increasing precursor mRNA splicing of *NCU01977.1*. Thiamine has no impact on the splicing of RNA lacking the TPP riboswitch (*24*). Although the *THI4* riboswitch mechanism has not been fully studied, it has been shown that an excess of TPP in the medium results in the synthesis of longer, nonfunctional mRNA (*24*). Both *Bcthi4* and *Bcnmt1*, which are involved in thiazole and pyrimidine synthesis in thiamine biosynthesis, exhibited up-regulated AS events and A5SS patterns. According to these findings, compound Z24 induced AS of *Bcthi4* and *Bcnmt1* precursor mRNAs, enhancing their splicing. Further investigation revealed that the *Bcthi4* gene produced two distinct AS transcripts. Under Z24 stress, the transcription level of the *Bcthi4* gene in *B. cinerea* increased in a concentration-dependent manner, ranging from 1.4 to 11.2 times. These results demonstrated that AS plays an increasingly important role in the regulation of eukaryotic thiamine biosynthesis genes (*33*, *34*). When extracellular thiamine levels are low, the ribosome binding site becomes available, TPP is not bound, and translation continues, leading to increased transcription of downstream genes. Compound Z24 regulates thiamine metabolism in *B. cinerea* by altering the binding of TPP to the *Bcthi4* riboswitch. In response to intracellular thiamine deficiency, *B. cinerea* up-regulates the expression of thiamine synthesis genes through a feedback mechanism. The high metabolic cost of thiamine production may explain the presence of a feedback control mechanism in organisms with a complete biosynthesis pathway. The TPP riboswitch is the primary riboswitch in fungi, often involved in the feedback regulation of metabolite production genes. After treatment with Z24, the expression of *Bcthi4* in *B. cinerea* cells increased rapidly, leading to elevated intracellular thiamine levels and improved cell survival. In summary, Z24 inhibits thiamine biosynthesis by targeting Bcthi4, altering the binding of TPP to the *Bcthi4* riboswitch, which disrupts thiamine metabolism in *B. cinerea*.

To sum up, neocryptolepine and its derivatives were designed and synthesized in our previous study (*7*). A novel derivative, Z24, was successfully synthesized and demonstrated high efficacy against all tested plant pathogenic fungi, with an EC_50_ range of 0.52 to 4.93 μg/ml, while exhibiting a favorable safety profile both in vitro and in vivo. Compound Z24 also showed more potent therapeutic and protect effects than pyrimethanil in vivo. Using DARTS technique, Bcthi4, involved in thiamine biosynthesis, was identified as a direct target of Z24, and its binding activity was further confirmed by DARTS, CETSA, and SPR. By inhibiting Bcthi4, Z24 disrupts the thiamine biosynthesis pathway in *B. cinerea*, thereby protecting plant cells from fungal infection. Given its previously unknown mechanism of action, the risk of cross-resistance between Z24 and existing fungicides is relatively low. To the best of our knowledge, compound Z24 is the first molecule discovered to inhibit the catalytic activity of Bcthi4 in fungi, representing a potential previously unidentified class of fungicides. In conclusion, compound Z24, with its safety profile and previously unknown mechanism of action, demonstrates anti–*B. cinerea* activity through targeting Bcthi4, making it a promising chemical entity for fungicide development.

## MATERIALS AND METHODS

### Chemicals, reagents, fungal strains, cells, and experimental animals

Compounds Z1 and Z24 were synthesized in our previous study (*7*). Pyrimethanil (purity ≥ 98%; CAS no. 53112-28-0), carbendazim (purity ≥ 98%; CAS no. 10605-21-7), and tebuconazole (purity ≥ 99%; CAS no. 107534-96-3) were purchased from Bide Pharmatech Ltd., Shanghai, China. Triton X-100 (20%; CAS no. 9002-93-1) and thiamine (purity ≥ 99%; CAS no. 59-43-8) were obtained from Yuanye Biotechnology Co. Ltd., Shanghai, China. *F. graminearum* Sehw, *B. cinerea* Pers., *R. solani* Kuhn, *S. sclerotiorum* (Lib.) de Bary, and *F. oxysporum* f. sp. vasinfectum (Atk.) Snyder & Hansen were provided by the Institute of Plant Protection, Gansu Academy of Agricultural Science. The standard strain *B. cinerea* B05.10 and *P. capsici* Leonian were provided by Nanjing Agricultural University and Sichuan Agricultural University, respectively.

HIECs, HL-7702 cells, and GES-1 cells were stored in our laboratory. All specific pathogen–free animals were purchased from the Veterinary Institute, Chinese Academy of Agricultural Sciences (Lanzhou, China). In vivo acute toxicity studies were conducted under the approval and supervision of the Institutional Animal Care and Use Committee of Lanzhou University [approval no. SCXK (甘) 2023-0003].

### Antifungal susceptibility tests in vitro

The fungitoxicity of Z24 against *B. cinerea* Pers. and *B. cinerea* B05.10 were assessed by measuring spore germination and mycelial growth. The effect of Z24 on *B. cinerea* spore germination was determined using previously reported methods (*66*). Z24 was dissolved in DMSO to prepare a stock solution of 100 μg/ml. This stock solution was then added to liquid PDA medium containing conidial suspension (1.5 × 10^6^ spores/ml) to obtain final concentrations of 0, 2, 4, 6, 8, 10, 12, 16, and 20 μg/ml. Spores were incubated at 26°C for 10 hours to assess the spore germination rate. Three replicates were performed for each experiment.

The inhibitory activity of Z24 against phytopathogenic fungi was determined using previously described methods (*67*). Briefly, PDA medium containing various concentrations of Z24 was prepared and poured into sterilized petri dishes for testing. As a blank control, 0.25% (v/v) DMSO was added to the PDA medium. A 5-mm fungal plug was inoculated at the center of each plate, which was then incubated in the dark at 26° ± 1°C (28°C for molds). Three replicates were performed for each parallel experiment. The rate of mycelial growth inhibition was calculated using the following formula

$$\text{Mycelial growth inhibition ratio}=\left[\frac{\left(\mathrm{dc}-\mathrm{dt}\right)}{\left(\mathrm{dc}-5\;\text{mm}\right)}\right]\times 100\%$$

where *dc* and *dt* represent the mean diameters in the control and treatment groups, respectively.

### Anti–*B. cinerea* efficacy in vivo

Protective effect evaluation (*B. cinerea* Pers. spores): Compound Z24 was tested for its protective effect on inoculated tomatoes using previously reported methods (*33*). The fruits were first surface-sterilized for 5 min with 2% (v/v) sodium hypochlorite, then washed with 75% ethanol, and rinsed with sterile distilled water. Each fruit was punched (3 mm deep by 3 mm wide) at the equatorial section and inoculated with 10 μl of *B. cinerea* Pers. conidial suspension (1.5 × 10^6^ spores/ml) after 24 hours of treatment with Z24 (0, 25, 50, and 100 μg/ml), respectively. The fruits were cultured at 26° ± 1°C for 3 days. Five replicates were performed for each treatment group. For the cucumber leaves, they were washed twice with sterile distilled water and dried naturally. Then, Z24 (100 μg/ml) and pyrimethanil (20 ml per pot) were evenly sprayed onto the surface of each cucumber leaf, and sterile water containing the same amount of DMSO was used as a blank control. After 24 hours of spraying Z24, 10 μl of *B. cinerea* Pers. conidial suspension (1.5 × 10^7^ spores/ml) was inoculated. The pots were incubated at 26° ± 1°C for 7 days. For each parallel experiment, eight to nine biological replicates were performed. Pyrimethanil was used as a positive control. The lesion area was determined using ImageJ software.

Protective effect evaluation (*B. cinerea* Pers. mycelia): To assess Z24’s ability to protect cucumber leaves from *B. cinerea* Pers. mycelium, each pot of cucumber leaves was sprayed with 20 ml of various Z24 concentrations, mixed with 0.1% (v/v) Tween 80. After 24 hours of spraying, freshly cultured *B. cinerea* Pers. plugs were inoculated and cultured for 48 and 72 hours under 12-hour light, 26° ± 1°C, 90% relative humidity. The diameters of the lesions on cucumber leaves were measured in millimeters.

Therapeutic effect evaluation (*B. cinerea* Pers. mycelia): After 24 hours of inoculation with *B. cinerea* Pers. plugs, each pot was sprayed with 20 ml of Z24 solution, and other experimental conditions were the same as described above.

### Sclerotia formation assay

For the sclerotia formation inhibition assay (*68*), PDA plates treated with different concentrations of Z24 were inoculated with 5-mm *S. sclerotiorum* plugs. The plates were then covered with tin foil and incubated at 26° ± 1°C for 15 days. Last, the wet weight of formed sclerotium was measured.

### Transmission electron microscopy

TEM observation was performed following the method described in our previous study (*66*). *B. cinerea* mycelia treated with Z24 (1 μg/ml) were incubated at 25° ± 1°C for 4 days. The mycelia were then fixed with 2.5% glutaraldehyde at 4°C for 4 hours, rinsed three times with 0.01 M phosphate-buffered saline (PBS) (pH 7.2), and dehydrated through a graded ethanol series (25, 50, 70, 95, and 100% three times), followed by dehydration in acetone. The dehydrated mycelia were subsequently embedded in Epon 812 and polymerized in Spurr’s resin for 48 hours at 60°C. Last, the samples were stained with 2.5% lead citrate and 2% uranyl acetate solutions before being examined under a TEM (JEM-1010 TEM; NEC, Japan).

### Fluorescence staining

Intracellular ROS level was determined using the fluorescent probe DCFH-DA (Solarbio, Beijing, China) according to the manufacturer’s instructions. *B. cinerea* Pers. spores were treated with Z24 (20 μg/ml) for 24 hours, collected by centrifugation at 4°C, and washed three times with 0.01 M PBS buffer (pH 7.2). After centrifuge at 6000 rpm for 30 min at 4°C, the supernatant was discarded. The spores were then incubated in a 10 μM solution of DCFH-DA at 37°C for 30 min in the dark. The dye solution was removed, and the spores were resuspended in PBS buffer. The spore suspension was photographed and examined under a fluorescence microscope (Zeiss Axioskop 40, Germany). For Hoechst 33342 and PI staining, *B. cinerea* Pers. mycelium treated with Z24 (0.5 μg/ml) was incubated with 5 μl of Hoechst 33342 (1 mg/ml) and 5 μl of PI (5 mg/ml) for 15 min at 37°C. After thoroughly removing the stains, the mycelium was observed and imaged under a confocal laser microscope.

### DARTS (SDS-PAGE analysis)

Total protein was extracted using a Fungal Protein Extraction Kit (Kanglang, Shanghai, China), and the protein was quantified to 5 mg/ml. Cell lysates were aliquoted into equivalent volumes containing 500 μg of protein and incubated for 60 min at 25°C with or without Z24. Pronase E from *Streptomyces griseus* (Solarbio, Beijing, China) was added to all samples at a pronase E:substrate mass ratio of 1:500 and incubated at 37°C for 30 min. To identify differential protein bands, SDS–polyacrylamide gel electrophoresis (PAGE) was performed, followed by Coomassie bright blue and silver staining.

### Mass spectrometry analysis

Gel bands were excised and prepared for mass spectrometry analysis using trypsin digestion, as described in Supplementary Text. Mass spectrometry was performed using Thermo’s Q Exactive Plus liquid chromatography–mass spectrometry system. The mass spectrometry data generated by the Q Exactive Plus were analyzed using MaxQuant (V1.6.2.10) and the MaxLFQ database search algorithm. The Proteome Reference Database of standard strain *B. cinerea* B05.10 was used for the retrieval.

### Molecular modeling analysis

The 3D structure of Bcthi4 was predicted using the AphaFold3 server (https://golgi.sandbox.google.com/) (*69*). The SDF format of the two-dimensional (2D) structure of Z24 (ligand) was obtained from the PubChem database (https://pubchem.ncbi.nlm.nih.gov/). The 3D structure of the receptor was processed by dehydrogenating and dewetting using AutoDock tools. Molecular docking analysis was performed using AutoDock vina, and docking interactions between the receptor and Z24 were visualized using PyMOL software.

### Rabbit polyclonal antibody preparation

Primers were designed on the basis of the *B. cinerea* B05.10 *Bcthi4* gene sequence (Gene ID: 5430120) from GenBank. The recombinant plasmid *Bcthi4*-pET-B2M was constructed by amplifying the *Bcthi4* gene via PCR and inserting it into the pET-B2M vector. The *Bcthi4*-pET-B2M plasmid was then transferred into *Escherichia coli* BL21 (DE3) cells, and high-purity recombinant Bcthi4 protein was obtained after isopropyl-β-d-thiogalactopyranoside (IPTG)–induced culture. Following animal immunization, indirect enzyme-linked immunosorbent assay (ELISA) and Western blotting assays confirmed that the rabbit polyclonal antibody against the *Bcthi4* gene exhibited high sensitivity and specificity, making it suitable for further Western blot applications. The specific binding of Z24 to Bcthi4 was subsequently detected by Western blot analysis.

### DARTS assay and CETSA (Western blot)

For the DARTS assay, 100 μl of lysates (5 mg/ml) from *B. cinerea* strain were incubated with DMSO or Z24 (100, 250, and 500 μg/ml). Pronase E was added to all samples at a pronase E:substrate mass ratio of 1:500, and the mixture was incubated at 37°C for 30 min. The reaction was terminated by adding 5 μl of protease inhibitor and incubating on ice for 5 min. For the CETSA, 100 μl of lysates (5 mg/ml) from *B. cinerea* strain were incubated with DMSO or Z24 (500 μg/ml) at temperatures ranging from 50° to 80°C for 5 min. The samples were then centrifuged at 10,000*g* for 15 min at 4°C to separate the supernatant and pellet. The supernatant (32 μl) was mixed with 8 μl of 5× loading buffer and heated in a metal bath for 10 min. The samples were then separated on a 12% SDS-PAGE gel for immunoblotting analysis of Bcthi4. After exposure, the grayscale values of the Western blot bands were analyzed using ImageJ software.

### Expression and purification of the Bcthi4 protein

The pET-SUMO plasmids expressing His-tagged Bcthi4 were transformed into *E. coli* BL21 Star (DE3) cells. Protein expression was induced with 0.5 mM IPTG, and the culture was incubated at 18°C for 18 hours with shaking at 200 rpm. After incubation, the bacterial cells were lysed by sonication, and the collected supernatant was loaded onto a Ni–nitrilotriacetic acid Superflow affinity column. The His-tagged Bcthi4 fusion protein was then eluted with 300 mM imidazole. The eluted protein was analyzed by SDS-PAGE. The primers used for protein expression are listed in table S3.

### SPR analysis

Binding between Z24 and Bcthi4 was assessed using SPR with a Biacore T200 instrument (Cytiva, USA). Z24 was prepared at concentrations of 0, 6.25, 12.5, 25, 50, and 100 μM in running buffer (0.05% polysorbate 20 and 5% DMSO). Bcthi4 was immobilized as the ligand on a CM5 sensor chip (Cytiva, USA), and Z24 was used as the analyte.

### Thiamin thiazole synthase activity

A 5-mm plug from the edge of 3-day-old colonies (*B. cinerea* Pers. and *B. cinerea* B05.10) was inoculated into liquid PDA medium supplemented with Z24 (0.05 and 0.10 μg/ml) and then incubated at 26° ± 1°C and 170 rpm for 3 days under darkness/light conditions. As a blank control, 0.25% (v/v) DMSO was used. The activity of Bcthi4 was measured using a thiazole synthase (Thi4) kit (Shanghai XinYu Bio-Technology Co. Ltd.) according to the manufacturer’s protocol.

### RNA-seq and AS analysis

Total RNA from *B. cinerea* was extracted using the TRIzol method, following the manufacturer’s protocol. The RNA samples were then sent to Wuhan Ruixing Biotechnology Co. Ltd. (Wuhan, China) for RNA-seq analysis. Differential expression was defined as a fold change ≥ 2 or ≤ 0.5 and a *P* value (probability value) < 0.05. HISAT2 software was used to evaluate the splice junctions of each sample. AS events were considered differential if the *P* value ≤ 0.05 compared to the DMSO group. The signaling pathways were analyzed using the KEGG (https://www.kegg.jp/).

### Reverse transcription quantitative polymerase chain reaction

Total RNA was extracted with a Fungal RNA Kit (Feiyang, Guangzhou, China). RNA reverse transcription was performed following the instructions of the FastKing gDNA Dispelling RT SupperMix Reverse Transcription Kit (Tiangen, Beijing, China), with TransScript One-Step gDNA Removal. RT-qPCR was then performed according to the instructions of the SuperReal PreMix Plus (SYBR Green) Fluorescence Quantitation Kit (Tiangen, Beijing, China) in an Eco 48 real-time fluorescent quantitative PCR system. Glyceraldehyde-3-phosphate dehydrogenase was used as the internal control. Primer sequences specific to each gene are listed in table S4. Relative transcription abundance was calculated using the 2^−ΔΔ*CT*^ method.

### Thiamine and TPP content assay

A 5-mm plug from the edge of 3-day-old colonies (*B. cinerea* Pers. and *B. cinerea* B05.10) was inoculated into liquid PDA medium supplemented with Z24 (0, 0.05, 0.10, and 0.20 μg/ml) and incubated at 26° ± 1°C with shaking at 170 rpm. A 0.25% (v/v) DMSO solution was used as a blank control. The same amount of mycelium was harvested for metabolite content determination. The contents of thiamine and TPP were measured using a VB1 ELISA kit (Shanghai Enzyme Linked Biotechnology Co. Ltd.) and a Fungi TPP ELISA kit (Shanghai Enzyme Linked Biotechnology Co. Ltd.) following the manufacturer’s protocol.

### Metabolome analysis

Metabolomics analysis was performed by Shenzhen BGI Co. Ltd. with three biological replicates for each treatment group. Briefly, a 5-mm plug from the edge of 3-day-old colonies of *B. cinerea* Pers. was inoculated into liquid PDA medium treated with Z24 (0 and 0.1 μg/ml) and incubated at 26 ± 1°C with shaking at 170 rpm for 3 days in darkness/light. A 0.25% (v/v) DMSO solution was used as a blank control. Waters 2D Ultra Performance Liquid Chromatography (UPLC; Waters, USA) in tandem with a Q Exactive high-resolution mass spectrometer (Thermo Fisher Scientific, USA) was used for the separation and detection of metabolites. Multivariate statistical analysis [PCA and Partial Least Squares Discriminant Analysis (PLS-DA)] and univariate analysis (fold change/Student’s *t* test) were combined to screen for differential metabolites between groups.

### In vitro cytotoxicity assay (MTT assay)

The cytotoxicity of Z1 and Z24 to HIECs, HL-7702 cells, and GES-1 cells was evaluated in vitro. HIECs, HL-7702 cells, and GES-1 cells were cultured at 37°C in RPMI medium (HyClone, USA) and Dulbecco’s modified Eagle’s medium high-glucose medium (HyClone, USA) containing 1% penicillin/streptomycin and 10% fetal bovine serum with 5% CO_2_. The cell inhibition rate was determined colorimetrically using MTT. In brief, 100 μl of exponentially growing HIECs, HL-7702 cells, and GES-1 cells were plated at a density of 6000 cells per well in a 96-well plate and cultured for 24 hours in an incubator at 37°C with 5% CO_2_. The cells were then treated with various concentrations of Z1 and Z24 (2.5, 5, 10, and 25 μM) in complete medium for 48 hours, with pyrimethanil as a positive control. After treatment, each well received 10 μl of MTT solution (5 mg/ml) and was incubated in the dark for 4 hours. A microplate reader was used to measure the absorbance at 490 nm. Four replicate wells were used for each condition.

### In vivo acute toxicity test

The acute oral toxicity test was conducted using six nulliparous, nonpregnant female rats in compliance with OECD Guideline 423 (*70*). Given that females are more sensitive than males in toxicity tests, the use of female rats was chosen to provide a more conservative estimate of the toxic effects (*71*). Z24, DMSO, and more than 95% pure corn oil were used to prepare the Z24 suspension. First, Z24 suspension was administered to three randomly selected female rats (*n* = 3) at a dose of 2000 mg/kg. The animals were monitored for 2 weeks, and any instances of death were recorded. After the observation period, blood and tissues from the heart, liver, spleen, lung, and kidney were collected following a fasting period. These samples were then used for histological assessment (hematoxylin and eosin staining). The liver and kidney functions were evaluated by measuring GOT, GPT, ALP, and urea nitrogen levels.

### Hemolysis assay

The hemolysis assay was performed following the National Cancer Institute procedure (*72*). Briefly, erythrocytes were isolated from sheep blood by centrifugation at 1500 rpm for 15 min. The collected erythrocytes were diluted in a 0.9% sodium chloride solution to achieve a final concentration of 4%. The compound Z24 to be tested was added to the erythrocyte suspension, with PBS and Triton X-100 used as the negative and positive controls, respectively. The mixture was incubated at 37°C for 1 hour at 60 rpm in a rotatory shaker, followed by centrifugation at 1000*g* for 3 min. Then, 100 μl of the supernatant was transferred to a 96-well plate, and a multifunctional microplate reader was used to measure the absorbance at 450 nm

$$\text{Hemolysis ratio}\;\left(\%\right)=\left[\frac{\left(\mathrm{As}-\text{An}\right)}{\left(\mathrm{Ap}-\text{An}\right)}\right]\times 100\%$$

where As, Ap, and An represent the absorbance of the compound Z24, Triton X-100, and PBS, respectively.

### Phytotoxicity assay

The phytotoxicity of Z24 was assessed using an in vitro seed germination experiment on Murashige and Skoog agar plates, as previously described (*66*). *R. raphanistrum* seeds were sterilized by immersing them in a 1% sodium hypochlorite solution for 20 min after soaking overnight in sterile distilled water. After three rinses with sterilized water, the seeds were placed on Murashige and Skoog agar plates with Z24 concentrations ranging from 10 to 200 μg/ml. Rhizome length and seed germination rate were assessed after 3 days of incubation at 26° ± 1°C.

### Statistical analysis

SPSS 26.0 was used to statistically analyze the experimental data, which are presented as means ± SD. Data were further analyzed using GraphPad Prism 9.5 software (San Diego, CA, USA). One-way analysis of variance (ANOVA) followed by Duncan’s multi range test was performed to compare the statistical differences between groups. Significant differences were considered when *P* < 0.05.
