# Supplementary material for: Indoloquinoline alkaloid neocryptolepine derivative inhibits Botrytis cinerea by targeting thiamine thiazole synthase
Source: Sci Adv. 2025 Mar 12;11(11):eadq5329. doi: 10.1126/sciadv.adq5329 (PMC11900860; doi:10.1126/sciadv.adq5329)
Supplement: Supplementary file 1 — Tables S1 to S4 Figs. S1 to S8 Supplementary Text [file sciadv.adq5329_sm.pdf]

Science Advances
AAAS

Supplementary Materials for

Indoloquinoline alkaloid neocryptolepine derivative inhibits *Botrytis cinerea* by targeting thiamine thiazole synthase

Wen-Bin Zhao *et al.*

Corresponding author: Ying-Qian Liu, yqliu@lzu.edu.cn, liuyqlab@163.com;
Zhi-Jun Zhang, zhangzhijun198803@163.com

Sci. Adv. **11**, eadq5329 (2025)
DOI: 10.1126/sciadv.adq5329

This PDF file includes:

Tables S1 to S4
Figs. S1 to S8
Supplementary Text

Table S1. The identified potential targets of Z24 in the B1 band.

Accession	Gene	Description	Mw (kDa)	Peptides B1	Sequence coverage B1 [%]	Intensity B1	log2 (B1)	iBAQ B1	iBAQ B1[%]
A0A384JNK6	***Bcthi4***	Thiamine thiazole synthase OS=*Botryotinia fuckeliana* (strain B05.10) OX=332648 GN=Bcthi4 PE=3 SV=1	34.969	10	20.4	3399300000	31.6626	199960000	**37.108**
A0A384JFE4	***Bcnmt1***	Bcnmt1 OS=*Botryotinia fuckeliana* (strain B05.10) OX=332648 GN=Bcnmt1 PE=4 SV=1	38.422	13	27.2	914850000	29.769	43564000	**8.085**
A0A384JFG3	*BCIN_04g05070*	40S ribosomal protein S6 OS=*Botryotinia fuckeliana* (strain B05.10) OX=332648 GN=BCIN_04g05070 PE=3 SV=1	27.201	6	19.2	333790000	28.3144	27816000	5.162
A0A384JVB5	*Bcrps3*	Bcrps3 OS=*Botryotinia fuckeliana* (strain B05.10) OX=332648 GN=Bcrps3 PE=3 SV=1	28.512	9	30	389580000	28.5373	19479000	3.615
A0A384JCX7	*BCIN_03g06830*	Uricase OS=*Botryotinia fuckeliana* (strain B05.10) OX=332648 GN=BCIN_03g06830 PE=3 SV=1	33.584	7	17.5	264050000	27.9762	13897000	2.579
A0A384JTH0	*Bcef1a*	Elongation factor 1-alpha OS=*Botryotinia fuckeliana* (strain B05.10) OX=332648 GN=Bcef1a PE=3 SV=1	49.983	10	16.5	316200000	28.2363	12161000	2.257
A0A384JM89	*BCIN_07g01150*	Ubiquitin-like domain-containing protein OS=*Botryotinia fuckeliana* (strain B05.10) OX=332648 GN=BCIN_07g01150 PE=4 SV=1	14.566	4	21.9	86930000	26.3734	9658900	1.792
A0A384J9H1	*BCIN_02g04220*	Ribos_L4_asso_C domain-containing protein OS=*Botryotinia fuckeliana* (strain B05.10) OX=332648 GN=BCIN_02g04220 PE=4 SV=1	40.04	12	28.4	213010000	27.6663	8520400	1.581
A0A384JFX3	*BCIN_04g05190*	40S ribosomal protein S8 OS=*Botryotinia fuckeliana* (strain B05.10) OX=332648 GN=BCIN_04g05190 PE=3 SV=1	23.17	3	11.8	127100000	26.9214	8473500	1.573
A0A384JB76	*Bcmir1*	Bcmir1 OS=*Botryotinia fuckeliana* (strain B05.10) OX=332648 GN=Bcmir1 PE=3 SV=1	33.612	6	14.6	152220000	27.1816	8011800	1.487
A0A384J4J0	*BCIN_01g02200*	S4 RNA-binding domain-containing protein OS=*Botryotinia fuckeliana* (strain B05.10) OX=332648 GN=BCIN_01g02200 PE=3 SV=1	22.279	8	28.3	107100000	26.6744	7650100	1.42
A0A384JBI6	*BCIN_03g01010*	Uncharacterized protein OS=*Botryotinia fuckeliana* (strain B05.10) OX=332648 GN=BCIN_03g01010 PE=3 SV=1	33.957	8	20.4	144640000	27.1079	7612600	1.413
A0A384JCX1	*BCIN_03g06600*	Uncharacterized protein OS=*Botryotinia fuckeliana* (strain B05.10) OX=332648 GN=BCIN_03g06600 PE=3 SV=1	68.669	10	13.8	235320000	27.81	6192800	1.149
A0A384JEF1	*BCIN_04g00540*	Ribosomal protein L19 OS=*Botryotinia fuckeliana* (strain B05.10) OX=332648 GN=BCIN_04g00540 PE=3	22.464	5	17.5	73497000	26.1312	6124700	1.137

		SV=1							
A0A384JD98	*BCIN_03g06530*	Ribosomal_L7Ae domain-containing protein OS=*Botryotinia fuckeliana* (strain B05.10) OX=332648 GN=BCIN_03g06530 PE=4 SV=1	28.897	6	20.4	94154000	26.4885	5884600	1.092
A0A384K2W1	*BCIN_14g01740*	PABS domain-containing protein OS=*Botryotinia fuckeliana* (strain B05.10) OX=332648 GN=BCIN_14g01740 PE=3 SV=1	33.126	5	13.1	92619000	26.4648	5788700	1.074
A0A384J4F1	*BCIN_01g01800*	40S ribosomal protein S4 OS=*Botryotinia fuckeliana* (strain B05.10) OX=332648 GN=BCIN_01g01800 PE=3 SV=1	29.332	9	27.9	130730000	26.962	5684100	1.055
A0A384K2W2	*Bcrpl3*	Bcrpl3 OS=*Botryotinia fuckeliana* (strain B05.10) OX=332648 GN=Bcrpl3 PE=3 SV=1	44.251	7	12.2	126180000	26.9109	5257700	0.976
A0A384J7N8	*BCIN_01g10430*	AB hydrolase-1 domain-containing protein OS=*Botryotinia fuckeliana* (strain B05.10) OX=332648 GN=BCIN_01g10430 PE=4 SV=1	40.546	3	6.7	97669000	26.5414	4883500	0.906
A0A384JPY4	*Bcser2*	Bcser2 OS=*Botryotinia fuckeliana* (strain B05.10) OX=332648 GN=Bcser2 PE=3 SV=1	55.267	4	6.1	103190000	26.6207	4690300	0.87
A0A384J5V2	*Bcenol1*	Bcenol1 OS=*Botryotinia fuckeliana* (strain B05.10) OX=332648 GN=Bcenol1 PE=3 SV=1	47.154	4	9.6	115510000	26.7834	4442700	0.824
A0A384JXF3	*BCIN_11g02490*	Uncharacterized protein OS=*Botryotinia fuckeliana* (strain B05.10) OX=332648 GN=BCIN_11g02490 PE=4 SV=1	60.028	10	17	145370000	27.1152	4153400	0.771
A0A384JD42	*BCIN_03g07630*	Uncharacterized protein OS=*Botryotinia fuckeliana* (strain B05.10) OX=332648 GN=BCIN_03g07630 PE=4 SV=1	28.62	4	11.2	57869000	25.7863	4133500	0.767
A0A384K1P6	*BCIN_13g05810*	Aldedh domain-containing protein OS=*Botryotinia fuckeliana* (strain B05.10) OX=332648 GN=BCIN_13g05810 PE=3 SV=1	53.6	5	9.7	137350000	27.0333	4039600	0.75
A0A384JBQ4	*BCIN_03g02530*	Ribosomal_L2_C domain-containing protein OS=*Botryotinia fuckeliana* (strain B05.10) OX=332648 GN=BCIN_03g02530 PE=4 SV=1	27.405	4	13.4	51577000	25.6202	3684100	0.684
A0A384JZC3	*BCIN_12g04380*	Uncharacterized protein OS=*Botryotinia fuckeliana* (strain B05.10) OX=332648 GN=BCIN_12g04380 PE=4 SV=1	118.5	10	8.5	179130000	27.4164	2936500	0.545
A0A384JCX3	*RPS1*	40S ribosomal protein S1 OS=*Botryotinia fuckeliana* (strain B05.10) OX=332648 GN=RPS1 PE=3 SV=1	29.246	6	17.2	49760000	25.5685	2927100	0.543
A0A384JU32	*BCIN_09g06480*	Uncharacterized protein OS=*Botryotinia fuckeliana* (strain B05.10) OX=332648 GN=BCIN_09g06480 PE=4 SV=1	17.478	4	17.4	21876000	24.3828	2734500	0.507
A0A384JLV2	*BCIN_07g01540*	Tr-type G domain-containing protein OS=*Botryotinia*	93.459	7	8.2	147580000	27.1369	2635300	0.489

		fuckeliana (strain B05.10) OX=332648 GN=BCIN_07g01540 PE=4 SV=1							
A0A384J7A0	*Bcccp1*	Peroxidase OS=*Botryotinia fuckeliana* (strain B05.10) OX=332648 GN=Bcccp1 PE=3 SV=1	40.749	4	11	56165000	25.7432	2553000	0.474
A0A384K6I9	*Bcpre5*	Proteasome subunit alpha type OS=*Botryotinia fuckeliana* (strain B05.10) OX=332648 GN=Bcpre5 PE=3 SV=1	28.809	3	10.9	41964000	25.3226	2468500	0.458
A0A384K4Y8	*Bcgph1*	Alpha-1,4 glucan phosphorylase OS=*Botryotinia fuckeliana* (strain B05.10) OX=332648 GN=Bcgph1 PE=3 SV=1	99.74	7	7.4	136650000	27.0259	2356100	0.437
A0A384J4J9	*BCIN_01g02210*	Uncharacterized protein OS=*Botryotinia fuckeliana* (strain B05.10) OX=332648 GN=BCIN_01g02210 PE=4 SV=1	18.296	3	14.4	22999000	24.4551	2090800	0.388
A0A384JD09	*BCIN_03g05330*	SOR_SNZ domain-containing protein OS=*Botryotinia fuckeliana* (strain B05.10) OX=332648 GN=BCIN_03g05330 PE=3 SV=1	33.189	4	12.8	32341000	24.9469	2021300	0.375
A0A384J852	*BCIN_02g00470*	Uncharacterized protein OS=*Botryotinia fuckeliana* (strain B05.10) OX=332648 GN=BCIN_02g00470 PE=4 SV=1	31.545	5	15.6	31502000	24.9089	1968800	0.365
A0A384J453	*BCIN_01g00980*	S5 DRBM domain-containing protein OS=*Botryotinia fuckeliana* (strain B05.10) OX=332648 GN=BCIN_01g00980 PE=3 SV=1	28.37	6	14.1	33421000	24.9943	1965900	0.365
A0A384J440	*Bcfpr2*	Peptidylprolyl isomerase OS=*Botryotinia fuckeliana* (strain B05.10) OX=332648 GN=Bcfpr2 PE=4 SV=1	21.147	2	9.5	30752000	24.8742	1922000	0.357
A0A384JMC8	*BCIN_07g02610*	6-phosphogluconate dehydrogenase, decarboxylating OS=*Botryotinia fuckeliana* (strain B05.10) OX=332648 GN=BCIN_07g02610 PE=3 SV=1	54.71	4	8.5	53411000	25.6706	1841800	0.342
A0A384K1G6	*BCIN_13g04730*	Uncharacterized protein OS=*Botryotinia fuckeliana* (strain B05.10) OX=332648 GN=BCIN_13g04730 PE=4 SV=1	11.769	1	10.2	8780300	23.0658	1756100	0.326
A0A384JGC8	*Bcmet6*	Bcmet6 OS=*Botryotinia fuckeliana* (strain B05.10) OX=332648 GN=Bcmet6 PE=3 SV=1	86.237	8	9.4	75561000	26.1711	1717300	0.319
A0A384JBJ9	*BCIN_03g02740*	Arginine biosynthesis bifunctional protein ArgJ, mitochondrial OS=*Botryotinia fuckeliana* (strain B05.10) OX=332648 GN=BCIN_03g02740 PE=3 SV=1	50.324	2	4.6	49511000	25.5612	1707300	0.317
A0A384JJ19	*BCIN_06g00380*	Ribosomal_S17_N domain-containing protein OS=*Botryotinia fuckeliana* (strain B05.10) OX=332648 GN=BCIN_06g00380 PE=3 SV=1	18.395	3	16.1	14453000	23.7849	1605900	0.298
A0A384K3Z3	*Bcatp1*	ATP synthase subunit alpha OS=*Botryotinia fuckeliana*	59.85	7	10.8	52546000	25.6471	1501300	0.279

		(strain B05.10) OX=332648 GN=Bcatp1 PE=3 SV=1							
A0A384JWQ9	*Bcfba1*	Fructose-bisphosphate aldolase OS=*Botryotinia fuckeliana* (strain B05.10) OX=332648 GN=Bcfba1 PE=3 SV=1	39.241	1	2.2	26898000	24.681	1494400	0.277
A0A384JAG9	*Bcshm2*	Serine hydroxymethyltransferase OS=*Botryotinia fuckeliana* (strain B05.10) OX=332648 GN=Bcshm2 PE=3 SV=1	52.696	5	9.6	43303000	25.368	1493200	0.277
A0A384JA13	*BCIN_02g06790*	Histone H2B OS=*Botryotinia fuckeliana* (strain B05.10) OX=332648 GN=BCIN_02g06790 PE=3 SV=1	14.941	2	12.9	19299000	24.202	1484500	0.275
A0A384JBF3	*Bccat5*	Catalase OS=*Botryotinia fuckeliana* (strain B05.10) OX=332648 GN=Bccat5 PE=3 SV=1	57.482	3	4.7	49966000	25.5744	1469600	0.273
A0A384JU36	*BCIN_10g01260*	Uncharacterized protein OS=*Botryotinia fuckeliana* (strain B05.10) OX=332648 GN=BCIN_10g01260 PE=3 SV=1	23.031	3	12.4	23205000	24.4679	1365000	0.253
A0A384K3A6	*Bcrpl5*	Bcrpl5 OS=*Botryotinia fuckeliana* (strain B05.10) OX=332648 GN=Bcrpl5 PE=3 SV=1	34.022	3	5.4	20077000	24.259	1338400	0.248
A0A384K7X4	*Bcfdh1*	Formate dehydrogenase OS=*Botryotinia fuckeliana* (strain B05.10) OX=332648 GN=Bcfdh1 PE=3 SV=1	40.851	2	4.8	25909000	24.6269	1233700	0.229
A0A384JE84	*BCIN_04g01180*	Ribosomal_L18e/L15P domain-containing protein OS=*Botryotinia fuckeliana* (strain B05.10) OX=332648 GN=BCIN_04g01180 PE=4 SV=1	20.735	3	15.3	15457000	23.8818	1189000	0.221
A0A384J4W3	*Bcipp1*	Bcipp1 OS=*Botryotinia fuckeliana* (strain B05.10) OX=332648 GN=Bcipp1 PE=4 SV=1	32.741	3	9.3	19717000	24.2329	1159800	0.215
A0A384J759	*BCIN_01g09350*	60S ribosomal protein L36 OS=*Botryotinia fuckeliana* (strain B05.10) OX=332648 GN=BCIN_01g09350 PE=3 SV=1	11.771	2	17.9	5741000	22.4529	1148200	0.213
A0A384K1A0	*BCIN_13g04420*	Histone H4 OS=*Botryotinia fuckeliana* (strain B05.10) OX=332648 GN=BCIN_13g04420 PE=3 SV=1	11.356	1	9.7	6864900	22.7108	1144100	0.212
A0A384K7A2	*BCIN_16g04050*	Glutamate dehydrogenase OS=*Botryotinia fuckeliana* (strain B05.10) OX=332648 GN=BCIN_16g04050 PE=3 SV=1	49.045	2	4.4	33121000	24.9812	1142100	0.212
A0A384K2H3	*BCIN_14g01980*	Transketolase OS=*Botryotinia fuckeliana* (strain B05.10) OX=332648 GN=BCIN_14g01980 PE=3 SV=1	74.965	11	12	38112000	25.1837	1088900	0.202
A0A384JQ22	*BCIN_08g03530*	Uncharacterized protein OS=*Botryotinia fuckeliana* (strain B05.10) OX=332648 GN=BCIN_08g03530 PE=4 SV=1	13.097	2	14.9	9784600	23.2221	1087200	0.202
A0A384JTW8	*BCIN_10g00300*	HATPase_c domain-containing protein OS=*Botryotinia fuckeliana* (strain B05.10) OX=332648 GN=BCIN_10g00300 PE=3 SV=1	79.536	5	7.1	41971000	25.3229	999310	0.185

A0A384JXH5	*Bcsah1*	Adenosylhomocysteinase OS=*Botryotinia fuckeliana* (strain B05.10) OX=332648 GN=Bcsah1 PE=3 SV=1	48.849	6	11.6	23972000	24.5148	998830	0.185
A0A384JBP8	*BCIN_03g03150*	Uncharacterized protein OS=*Botryotinia fuckeliana* (strain B05.10) OX=332648 GN=BCIN_03g03150 PE=4 SV=1	34.108	1	3.1	21204000	24.3378	963820	0.179
A0A384JYI5	*Bcidi1*	Bcidi1 OS=*Botryotinia fuckeliana* (strain B05.10) OX=332648 GN=Bcidi1 PE=4 SV=1	29.258	3	9.4	16078000	23.9386	945780	0.176
A0A384JSX9	*Bcglk*	Phosphotransferase OS=*Botryotinia fuckeliana* (strain B05.10) OX=332648 GN=Bcglk PE=3 SV=1	60.598	3	5.5	35529000	25.0825	934980	0.174
A0A384J6L3	*Bcpre10*	Proteasome subunit alpha type OS=*Botryotinia fuckeliana* (strain B05.10) OX=332648 GN=Bcpre10 PE=3 SV=1	31.986	2	7	15841000	23.9172	931820	0.173
A0A384K6F2	*BcactA*	BcactA OS=*Botryotinia fuckeliana* (strain B05.10) OX=332648 GN=BcactA PE=3 SV=1	41.639	2	5.9	20804000	24.3104	904510	0.168
A0A384JV25	*BCIN_10g04610*	40S ribosomal protein S7 OS=*Botryotinia fuckeliana* (strain B05.10) OX=332648 GN=BCIN_10g04610 PE=3 SV=1	22.526	1	4	9709900	23.211	882720	0.164
A0A384K1U9	*BCIN_13g04410*	Histone H3 OS=*Botryotinia fuckeliana* (strain B05.10) OX=332648 GN=BCIN_13g04410 PE=3 SV=1	15.319	1	5.1	5255800	22.3255	875960	0.163
A0A384JE47	*Bclys9*	Bclys9 OS=*Botryotinia fuckeliana* (strain B05.10) OX=332648 GN=Bclys9 PE=4 SV=1	49.322	1	2.5	28264000	24.7525	807550	0.15
A0A384JM47	*BCIN_07g02370*	DJ-1_PfpI domain-containing protein OS=*Botryotinia fuckeliana* (strain B05.10) OX=332648 GN=BCIN_07g02370 PE=4 SV=1	31.615	2	7.4	11571000	23.464	771370	0.143
A0A384J9Y2	*BCIN_02g06580*	Uncharacterized protein OS=*Botryotinia fuckeliana* (strain B05.10) OX=332648 GN=BCIN_02g06580 PE=3 SV=1	62.692	4	7.5	23672000	24.4967	763600	0.142
A0A384K4A5	*BCIN_15g02120*	Glyceraldehyde-3-phosphate dehydrogenase OS=*Botryotinia fuckeliana* (strain B05.10) OX=332648 GN=BCIN_15g02120 PE=3 SV=1	36.599	4	12.4	12919000	23.623	759940	0.141
A0A384JTU5	*Bcrpl10*	Bcrpl10 OS=*Botryotinia fuckeliana* (strain B05.10) OX=332648 GN=Bcrpl10 PE=4 SV=1	25.372	1	4.1	11025000	23.3943	735020	0.136
A0A384K179	*BCIN_13g03600*	KOW domain-containing protein OS=*Botryotinia fuckeliana* (strain B05.10) OX=332648 GN=BCIN_13g03600 PE=3 SV=1	15.533	3	14	5780600	22.4628	722570	0.134
A0A384JC13	*Bcado1*	Bcado1 OS=*Botryotinia fuckeliana* (strain B05.10) OX=332648 GN=Bcado1 PE=4 SV=1	37.68	2	5.5	12029000	23.52	707590	0.131
A0A384JWV9	*BCIN_11g03280*	Uncharacterized protein OS=*Botryotinia fuckeliana* (strain B05.10) OX=332648 GN=BCIN_11g03280 PE=3	14.522	2	13.4	5659900	22.4323	707480	0.131

		SV=1							
A0A384J7J8	*BCIN_01g10140*	Uncharacterized protein OS=*Botryotinia fuckeliana* (strain B05.10) OX=332648 GN=BCIN_01g10140 PE=4 SV=1	29.434	2	8.4	9943500	23.2453	662900	0.123
A0A384JJL3	*Bcrpn5*	Bcrpn5 OS=*Botryotinia fuckeliana* (strain B05.10) OX=332648 GN=Bcrpn5 PE=4 SV=1	56.608	2	3	21917000	24.3855	644620	0.12
A0A384JZT0	*BCIN_12g06180*	Cyanide hydratase OS=*Botryotinia fuckeliana* (strain B05.10) OX=332648 GN=BCIN_12g06180 PE=2 SV=1	41.445	5	13.5	13404000	23.6762	638290	0.118
A0A384J5U4	*Bcfmp41*	Bcfmp41 OS=*Botryotinia fuckeliana* (strain B05.10) OX=332648 GN=Bcfmp41 PE=4 SV=1	26.579	2	7.5	13238000	23.6582	601710	0.112
A0A384JTD7	*BCIN_09g04320*	Uncharacterized protein OS=*Botryotinia fuckeliana* (strain B05.10) OX=332648 GN=BCIN_09g04320 PE=3 SV=1	42.839	1	2	6829300	22.7033	569110	0.106
A0A384JNQ0	*Bcgdi1*	Rab GDP dissociation inhibitor OS=*Botryotinia fuckeliana* (strain B05.10) OX=332648 GN=Bcgdi1 PE=3 SV=1	51.913	3	5.7	15228000	23.8602	543870	0.101
A0A384JY85	*BCIN_12g00910*	Uncharacterized protein OS=*Botryotinia fuckeliana* (strain B05.10) OX=332648 GN=BCIN_12g00910 PE=4 SV=1	17.091	1	5.1	2693000	21.3608	538600	0.1
A0A384JSF0	*BCIN_09g03550*	KOW domain-containing protein OS=*Botryotinia fuckeliana* (strain B05.10) OX=332648 GN=BCIN_09g03550 PE=4 SV=1	15.936	2	12.4	5880600	22.4875	534600	0.099
A0A384J776	*BCIN_01g09950*	Pyruvate carboxylase OS=*Botryotinia fuckeliana* (strain B05.10) OX=332648 GN=BCIN_01g09950 PE=4 SV=1	131.08	8	6.7	37246000	25.1506	532080	0.099
A0A384JI26	*BCIN_05g06130*	Uncharacterized protein OS=*Botryotinia fuckeliana* (strain B05.10) OX=332648 GN=BCIN_05g06130 PE=4 SV=1	14.04	2	12.8	5029300	22.2619	502930	0.093
A0A384J4N4	*BCIN_01g02580*	14_3_3 domain-containing protein OS=*Botryotinia fuckeliana* (strain B05.10) OX=332648 GN=BCIN_01g02580 PE=3 SV=1	29.623	4	12.8	8956000	23.0944	471370	0.087
A0A384K2T7	*Bcgbl1*	Bcgbl1 OS=*Botryotinia fuckeliana* (strain B05.10) OX=332648 GN=Bcgbl1 PE=4 SV=1	34.931	2	3.2	9423000	23.1678	471150	0.087
A0A384JBE1	*BCIN_03g01540*	Uncharacterized protein OS=*Botryotinia fuckeliana* (strain B05.10) OX=332648 GN=BCIN_03g01540 PE=4 SV=1	66.881	1	1.3	9892900	23.238	471090	0.087
A0A384K0D3	*Bckar2*	Bckar2 OS=*Botryotinia fuckeliana* (strain B05.10) OX=332648 GN=Bckar2 PE=3 SV=1	73.12	3	5.2	18293000	24.1248	469060	0.087
A0A384J7A7	*Bcpsd*	Bcpsd OS=*Botryotinia fuckeliana* (strain B05.10) OX=332648 GN=Bcpsd PE=4 SV=1	46.447	6	8.8	12104000	23.529	465530	0.086
A0A384JNL7	*Bchsp60*	Bchsp60 OS=*Botryotinia*	61.162	6	10.1	17864000	24.0906	458060	0.085

		fuckeliana (strain B05.10) OX=332648 GN=Bchsp60 PE=3 SV=1							
A0A384JEU9	*Bcprx1*	Bcprx1 OS=*Botryotinia fuckeliana* (strain B05.10) OX=332648 GN=Bcprx1 PE=4 SV=1	36.216	2	7.2	10389000	23.3086	451680	0.084
A0A384JBE3	*BCIN_03g01820*	Ribosomal protein OS=*Botryotinia fuckeliana* (strain B05.10) OX=332648 GN=BCIN_03g01820 PE=3 SV=1	24.178	1	6.5	5850800	22.4802	450060	0.084
A0A384JJU9	*BCIN_06g02380*	RmlD_sub_bind domain-containing protein OS=*Botryotinia fuckeliana* (strain B05.10) OX=332648 GN=BCIN_06g02380 PE=4 SV=1	32.992	3	8.9	7626400	22.8626	448610	0.083
A0A384J564	*Bcugp1*	UTP--glucose-1-phosphate uridylyltransferase OS=*Botryotinia fuckeliana* (strain B05.10) OX=332648 GN=Bcugp1 PE=3 SV=1	58.609	4	6.7	15110000	23.849	444410	0.082
A0A384JBU2	*BCIN_03g03360*	Transaldolase OS=*Botryotinia fuckeliana* (strain B05.10) OX=332648 GN=BCIN_03g03360 PE=3 SV=1	35.402	5	17.3	9665600	23.2044	439350	0.082
A0A384JV88	*Bcuga1*	Bcuga1 OS=*Botryotinia fuckeliana* (strain B05.10) OX=332648 GN=Bcuga1 PE=3 SV=1	54.692	3	6.7	11211000	23.4184	431180	0.08
A0A384K383	*BCIN_14g02680*	40S ribosomal protein S24 OS=*Botryotinia fuckeliana* (strain B05.10) OX=332648 GN=BCIN_14g02680 PE=3 SV=1	15.439	1	6.7	3848800	21.876	427650	0.079
A0A384JH24	*BCIN_05g00940*	Uncharacterized protein OS=*Botryotinia fuckeliana* (strain B05.10) OX=332648 GN=BCIN_05g00940 PE=4 SV=1	59.3	2	3	14705000	23.8098	408460	0.076
A0A384K2N5	*Bcsfa1*	S-(hydroxymethyl)glutathione dehydrogenase OS=*Botryotinia fuckeliana* (strain B05.10) OX=332648 GN=Bcsfa1 PE=3 SV=1	40.595	1	2.4	8573600	23.0315	408270	0.076
A0A384J7Q3	*Bczwf1*	Glucose-6-phosphate 1-dehydrogenase OS=*Botryotinia fuckeliana* (strain B05.10) OX=332648 GN=Bczwf1 PE=3 SV=1	58.669	3	6.1	16605000	23.9851	405000	0.075
A0A384K3C6	*BCIN_14g04790*	Ribosomal protein L15 OS=*Botryotinia fuckeliana* (strain B05.10) OX=332648 GN=BCIN_14g04790 PE=3 SV=1	23.944	2	11.8	4806200	22.1965	400520	0.074
A0A384K4A0	*Bcatp2*	ATP synthase subunit beta OS=*Botryotinia fuckeliana* (strain B05.10) OX=332648 GN=Bcatp2 PE=3 SV=1	54.546	5	10	11731000	23.4838	391030	0.073
A0A384JKW4	*Bcrps13*	Bcrps13 OS=*Botryotinia fuckeliana* (strain B05.10) OX=332648 GN=Bcrps13 PE=3 SV=1	16.814	2	15.2	4290800	22.0328	390070	0.072
A0A384JDA3	*BCIN_03g06810*	MIF4G domain-containing protein OS=*Botryotinia fuckeliana* (strain B05.10) OX=332648 GN=BCIN_03g06810 PE=4 SV=1	150.82	2	1.3	31123000	24.8915	389030	0.072
A0A384K177	*Bcmeu1*	S-methyl-5-thioadenosine phosphorylase OS=*Botryotinia fuckeliana*	33.777	1	3.2	5300600	22.3377	378620	0.07

		(strain B05.10) OX=332648 GN=Bcmeu1 PE=3 SV=1							
A0A384J4Q0	*Bccct2*	Bccct2 OS=*Botryotinia fuckeliana* (strain B05.10) OX=332648 GN=Bccct2 PE=3 SV=1	56.693	2	3.2	13144000	23.6479	375560	0.07
A0A384J5X2	*BCIN_01g05720*	Uncharacterized protein OS=*Botryotinia fuckeliana* (strain B05.10) OX=332648 GN=BCIN_01g05720 PE=4 SV=1	82.549	1	1	7509800	22.8403	375490	0.07
A0A384J6Z0	*Bcpic7*	Pyruvate kinase OS=*Botryotinia fuckeliana* (strain B05.10) OX=332648 GN=Bcpic7 PE=3 SV=1	57.905	4	6.5	13507000	23.6872	375180	0.07
A0A384K7U0	*BCIN_16g03380*	Uncharacterized protein OS=*Botryotinia fuckeliana* (strain B05.10) OX=332648 GN=BCIN_16g03380 PE=4 SV=1	28.355	4	12.8	7086700	22.7567	372980	0.069
A0A384JXL0	*BCIN_11g05900*	60S ribosomal protein L6 OS=*Botryotinia fuckeliana* (strain B05.10) OX=332648 GN=BCIN_11g05900 PE=3 SV=1	22.137	2	8	5420000	22.3699	361330	0.067
A0A384JBH8	*BCIN_03g02320*	Eukaryotic translation initiation factor 3 subunit L OS=*Botryotinia fuckeliana* (strain B05.10) OX=332648 GN=BCIN_03g02320 PE=3 SV=1	54.853	3	5.5	8433100	23.0076	351380	0.065
A0A384JQ57	*Bcpme1*	Pectinesterase OS=*Botryotinia fuckeliana* (strain B05.10) OX=332648 GN=Bcpme1 PE=4 SV=1	37.236	1	3.5	3103300	21.5654	344810	0.064
A0A384JT42	*BCIN_09g05010*	Uncharacterized protein OS=*Botryotinia fuckeliana* (strain B05.10) OX=332648 GN=BCIN_09g05010 PE=4 SV=1	53.192	1	1.4	9406200	23.1652	335940	0.062
A0A384K4P1	*Bcaim24*	Altered inheritance of mitochondria protein 24, mitochondrial OS=*Botryotinia fuckeliana* (strain B05.10) OX=332648 GN=Bcaim24 PE=3 SV=1	41.249	3	7.5	9708700	23.2108	334780	0.062
A0A384J6S6	*Bclgd1*	Bclgd1 OS=*Botryotinia fuckeliana* (strain B05.10) OX=332648 GN=Bclgd1 PE=4 SV=1	46.906	2	4.7	8082700	22.9464	323310	0.06
A0A384JSI3	*BCIN_09g02990*	Uncharacterized protein OS=*Botryotinia fuckeliana* (strain B05.10) OX=332648 GN=BCIN_09g02990 PE=4 SV=1	41.41	1	2.3	6452100	22.6213	322610	0.06
A0A384J8S2	*BCIN_02g02750*	Citrate synthase OS=*Botryotinia fuckeliana* (strain B05.10) OX=332648 GN=BCIN_02g02750 PE=3 SV=1	52.059	2	4	8595800	23.0352	318360	0.059
A0A384J667	*Bcpol30*	Proliferating cell nuclear antigen OS=*Botryotinia fuckeliana* (strain B05.10) OX=332648 GN=Bcpol30 PE=3 SV=1	28.38	2	6.6	5045800	22.2667	315360	0.059
A0A384J8L1	*BCIN_02g02100*	Uncharacterized protein OS=*Botryotinia fuckeliana* (strain B05.10) OX=332648 GN=BCIN_02g02100 PE=3 SV=1	56.632	1	2.8	8712100	23.0546	311150	0.058
A0A384JZN2	*Bclat1*	Acetyltransferase component of pyruvate dehydrogenase complex OS=*Botryotinia fuckeliana* (strain B05.10) OX=332648 GN=Bclat1	48.358	3	8	7762000	22.888	310480	0.058

		PE=3 SV=1							
A0A384JGS5	*BCIN_05g01600*	60S ribosomal protein L20 OS=*Botryotinia fuckeliana* (strain B05.10) OX=332648 GN=BCIN_05g01600 PE=3 SV=1	20.741	1	6.3	3657300	21.8023	304780	0.057
A0A384J888	*Bcqcr2*	Bcqcr2 OS=*Botryotinia fuckeliana* (strain B05.10) OX=332648 GN=Bcqcr2 PE=4 SV=1	48.148	2	4.3	8120400	22.9531	300760	0.056
A0A384JWP0	*BCIN_11g03320*	HMG box domain-containing protein OS=*Botryotinia fuckeliana* (strain B05.10) OX=332648 GN=BCIN_11g03320 PE=4 SV=1	37.408	2	5.7	6600100	22.6541	300000	0.056
A0A384JEJ7	*Bcoxp1*	Bcoxp1 OS=*Botryotinia fuckeliana* (strain B05.10) OX=332648 GN=Bcoxp1 PE=4 SV=1	149.48	5	3.3	23927000	24.5121	295390	0.055
A0A384JGL5	*BCIN_05g01550*	Aldo_ket_red domain-containing protein OS=*Botryotinia fuckeliana* (strain B05.10) OX=332648 GN=BCIN_05g01550 PE=4 SV=1	36.325	1	2.5	6277300	22.5817	285330	0.053
A0A384K7Y8	*Bctom40*	Bctom40 OS=*Botryotinia fuckeliana* (strain B05.10) OX=332648 GN=Bctom40 PE=4 SV=1	38.441	1	2.5	4225500	22.0107	281700	0.052
A0A384J3X8	*Bcfas2*	Bcfas2 OS=*Botryotinia fuckeliana* (strain B05.10) OX=332648 GN=Bcfas2 PE=3 SV=1	204.22	10	4.7	31605000	24.9136	279690	0.052
A0A384JWC1	*Bcuga2*	Bcuga2 OS=*Botryotinia fuckeliana* (strain B05.10) OX=332648 GN=Bcuga2 PE=4 SV=1	53.742	2	1.8	7171500	22.7738	275830	0.051
A0A384JYI9	*BCIN_11g05920*	Uncharacterized protein OS=*Botryotinia fuckeliana* (strain B05.10) OX=332648 GN=BCIN_11g05920 PE=4 SV=1	21.684	1	4.7	3549500	21.7592	273040	0.051
A0A384JFY2	*BCIN_04g06450*	Aldo_ket_red domain-containing protein OS=*Botryotinia fuckeliana* (strain B05.10) OX=332648 GN=BCIN_04g06450 PE=4 SV=1	38.127	2	5	4889300	22.2212	271630	0.05
A0A384J4R3	*Bcngr1*	Bcngr1 OS=*Botryotinia fuckeliana* (strain B05.10) OX=332648 GN=Bcngr1 PE=4 SV=1	44.221	1	2.2	4293200	22.0336	268330	0.05
A0A384JHT7	*BCIN_05g04910*	PH_6 domain-containing protein OS=*Botryotinia fuckeliana* (strain B05.10) OX=332648 GN=BCIN_05g04910 PE=4 SV=1	61.366	1	1.4	6144300	22.5508	267140	0.05
A0A384J909	*Bcyhm2*	Bcyhm2 OS=*Botryotinia fuckeliana* (strain B05.10) OX=332648 GN=Bcyhm2 PE=3 SV=1	33.74	2	5.7	4879900	22.2184	256840	0.048
A0A384JSI7	*Bcpdh1*	Bcpdh1 OS=*Botryotinia fuckeliana* (strain B05.10) OX=332648 GN=Bcpdh1 PE=4 SV=1	61.172	1	1.6	9271300	23.1443	250580	0.047
A0A384J8J7	*BCIN_02g03480*	Abhydrolase_3 domain-containing protein OS=*Botryotinia fuckeliana* (strain B05.10) OX=332648 GN=BCIN_02g03480 PE=4 SV=1	35.051	1	3.1	3440500	21.7142	245750	0.046
A0A384JGR8	*BCIN_05g0*	TPT domain-containing	43.767	1	1.5	2400800	21.1951	240080	0.045

	0570	protein OS=*Botryotinia fuckeliana* (strain B05.10) OX=332648 GN=BCIN_05g00570 PE=4 SV=1							
A0A384JD22	*BCIN_03g06990*	Uncharacterized protein OS=*Botryotinia fuckeliana* (strain B05.10) OX=332648 GN=BCIN_03g06990 PE=3 SV=1	15.9	2	13.3	2444900	21.2213	222260	0.041
A0A384JTX1	*Bcgsh2*	Glutathione synthetase OS=*Botryotinia fuckeliana* (strain B05.10) OX=332648 GN=Bcgsh2 PE=3 SV=1	56.442	2	3.9	6821100	22.7016	220040	0.041
A0A384JVY6	*BCIN_10g04300*	Uncharacterized protein OS=*Botryotinia fuckeliana* (strain B05.10) OX=332648 GN=BCIN_10g04300 PE=3 SV=1	17.89	2	11.5	2813400	21.4239	216410	0.04
A0A384JEA0	*BCIN_04g01450*	Uncharacterized protein OS=*Botryotinia fuckeliana* (strain B05.10) OX=332648 GN=BCIN_04g01450 PE=4 SV=1	44.269	1	2.3	6256700	22.577	215750	0.04
A0A384JA37	*BCIN_02g06400*	Dihydrolipoyl dehydrogenase OS=*Botryotinia fuckeliana* (strain B05.10) OX=332648 GN=BCIN_02g06400 PE=3 SV=1	54.421	3	5.5	6644100	22.6636	214330	0.04
A0A384JV63	*BCIN_10g04650*	DUF89 domain-containing protein OS=*Botryotinia fuckeliana* (strain B05.10) OX=332648 GN=BCIN_10g04650 PE=4 SV=1	53.056	2	3	6944600	22.7275	210440	0.039
A0A384K5B9	*Bcnsr1*	Bcnsr1 OS=*Botryotinia fuckeliana* (strain B05.10) OX=332648 GN=Bcnsr1 PE=4 SV=1	47.441	2	4.8	4621200	22.1398	192550	0.036
A0A384JVI2	*Bcrpg1*	Eukaryotic translation initiation factor 3 subunit A OS=*Botryotinia fuckeliana* (strain B05.10) OX=332648 GN=Bcrpg1 PE=3 SV=1	119.75	3	3.5	11852000	23.4986	179580	0.033
A0A384K036	*Bcarx1*	Bcarx1 OS=*Botryotinia fuckeliana* (strain B05.10) OX=332648 GN=Bcarx1 PE=4 SV=1	43.869	2	5.7	4350900	22.0529	174040	0.032
A0A384JPH0	*Bcgb1*	Bcgb1 OS=*Botryotinia fuckeliana* (strain B05.10) OX=332648 GN=Bcgb1 PE=4 SV=1	39.539	1	2.2	2721400	21.3759	170090	0.032
A0A384JLT4	*BCIN_06g07350*	Aldolase_II domain-containing protein OS=*Botryotinia fuckeliana* (strain B05.10) OX=332648 GN=BCIN_06g07350 PE=4 SV=1	33.132	3	9.8	3022900	21.5275	167940	0.031
A0A384JAP1	*BCIN_02g07930*	Uncharacterized protein OS=*Botryotinia fuckeliana* (strain B05.10) OX=332648 GN=BCIN_02g07930 PE=3 SV=1	30.864	1	3.4	2662100	21.3441	166380	0.031
A0A384JH41	*Bcsti1*	Bcsti1 OS=*Botryotinia fuckeliana* (strain B05.10) OX=332648 GN=Bcsti1 PE=4 SV=1	64.998	1	1.2	7174400	22.7744	163050	0.03
A0A384K629	*Bcecm31*	3-methyl-2-oxobutanoate hydroxymethyltransferase OS=*Botryotinia fuckeliana* (strain B05.10) OX=332648 GN=Bcecm31 PE=3 SV=1	35.376	1	3.1	2254300	21.1042	161020	0.03
A0A384JKP7	*BCIN_06g05920*	Uncharacterized protein OS=*Botryotinia fuckeliana* (strain B05.10) OX=332648	50.795	2	4.7	3320600	21.663	158120	0.029

		GN=BCIN_06g05920 PE=4 SV=1							
A0A384JX78	*BCIN_11g04630*	Uncharacterized protein OS=*Botryotinia fuckeliana* (strain B05.10) OX=332648 GN=BCIN_11g04630 PE=3 SV=1	42.944	2	4.9	3775000	21.848	157290	0.029
A0A384JYV3	*Bchis2*	Histidinol-phosphatase OS=*Botryotinia fuckeliana* (strain B05.10) OX=332648 GN=Bchis2 PE=3 SV=1	34.818	1	3.2	3452800	21.7193	156950	0.029
A0A384J809	*BCIN_01g11220*	Uncharacterized protein OS=*Botryotinia fuckeliana* (strain B05.10) OX=332648 GN=BCIN_01g11220 PE=3 SV=1	31.929	1	3.1	1839000	20.8105	153250	0.028
A0A384JAJ7	*BCIN_03g00010*	Uncharacterized protein OS=*Botryotinia fuckeliana* (strain B05.10) OX=332648 GN=BCIN_03g00010 PE=4 SV=1	55.225	4	8	4895500	22.223	152980	0.028
A0A384JXC1	*Bccbk1*	Bccbk1 OS=*Botryotinia fuckeliana* (strain B05.10) OX=332648 GN=Bccbk1 PE=4 SV=1	75.493	1	1.5	5321500	22.3434	152040	0.028
A0A384JUY8	*BCIN_10g04290*	Uncharacterized protein OS=*Botryotinia fuckeliana* (strain B05.10) OX=332648 GN=BCIN_10g04290 PE=3 SV=1	20.854	1	5.3	2123100	21.0177	151650	0.028
A0A384JMH8	*BCIN_07g03460*	Uncharacterized protein OS=*Botryotinia fuckeliana* (strain B05.10) OX=332648 GN=BCIN_07g03460 PE=4 SV=1	42.149	2	5.8	2674500	21.3508	148590	0.028
A0A384JVE0	*BCIN_10g05060*	Uncharacterized protein OS=*Botryotinia fuckeliana* (strain B05.10) OX=332648 GN=BCIN_10g05060 PE=4 SV=1	199.45	1	0.5	14993000	23.8378	145570	0.027
A0A384JGF9	*BCIN_05g00090*	Uncharacterized protein OS=*Botryotinia fuckeliana* (strain B05.10) OX=332648 GN=BCIN_05g00090 PE=3 SV=1	71.554	2	2.6	6254200	22.5764	145450	0.027
A0A384J9Y3	*Bcaco1*	Aconitate hydratase, mitochondrial OS=*Botryotinia fuckeliana* (strain B05.10) OX=332648 GN=Bcaco1 PE=3 SV=1	84.975	5	4.5	5839600	22.4774	142430	0.026
A0A384JEM7	*BCIN_04g01310*	Uncharacterized protein OS=*Botryotinia fuckeliana* (strain B05.10) OX=332648 GN=BCIN_04g01310 PE=4 SV=1	41.133	1	3.7	986060	19.9113	140870	0.026
A0A384JZJ1	*BCIN_12g05550*	Aminotran_1_2 domain-containing protein OS=*Botryotinia fuckeliana* (strain B05.10) OX=332648 GN=BCIN_12g05550 PE=4 SV=1	46.982	2	4.7	3733800	21.8322	138290	0.026
A0A384JGP9	*BCIN_05g01750*	Uncharacterized protein OS=*Botryotinia fuckeliana* (strain B05.10) OX=332648 GN=BCIN_05g01750 PE=4 SV=1	102.26	3	2.8	6520700	22.6366	135850	0.025
A0A384K7Z3	*BCIN_17g00010*	Aminotran_1_2 domain-containing protein OS=*Botryotinia fuckeliana* (strain B05.10) OX=332648 GN=BCIN_17g00010 PE=4 SV=1	47.067	1	1.6	2860800	21.448	124380	0.023
A0A384J5H7	*Bcarc40*	Actin-related protein 2/3 complex subunit OS=*Botryotinia fuckeliana*	39.351	1	2.2	2475800	21.2395	123790	0.023

		(strain B05.10) OX=332648 GN=Bcarc40 PE=3 SV=1							
A0A384JFP6	*BCIN_04g04500*	Uncharacterized protein OS=*Botryotinia fuckeliana* (strain B05.10) OX=332648 GN=BCIN_04g04500 PE=4 SV=1	29.981	2	7.4	2449600	21.2241	122480	0.023
A0A384JZ76	*Bcdao1*	Bcdao1 OS=*Botryotinia fuckeliana* (strain B05.10) OX=332648 GN=Bcdao1 PE=4 SV=1	38.644	2	3.9	2074000	20.984	122000	0.023
A0A384J885	*Bcrpt1*	Bcrpt1 OS=*Botryotinia fuckeliana* (strain B05.10) OX=332648 GN=Bcrpt1 PE=3 SV=1	49.026	3	7.5	4027900	21.9416	118470	0.022
A0A384K4H3	*Bcpre6*	Proteasome subunit alpha type OS=*Botryotinia fuckeliana* (strain B05.10) OX=332648 GN=Bcpre6 PE=3 SV=1	29.154	1	4.9	2244900	21.0982	118150	0.022
A0A384K1R6	*Bcarp2*	Actin-related protein 2 OS=*Botryotinia fuckeliana* (strain B05.10) OX=332648 GN=Bcarp2 PE=3 SV=1	44.036	1	2.1	2590400	21.3047	117750	0.022
A0A384JEX2	*Bcpio13*	Aspartate aminotransferase OS=*Botryotinia fuckeliana* (strain B05.10) OX=332648 GN=Bcpio13 PE=4 SV=1	46.088	2	4	3410800	21.7017	117610	0.022
A0A384JY21	*Bcgua1*	Bcgua1 OS=*Botryotinia fuckeliana* (strain B05.10) OX=332648 GN=Bcgua1 PE=3 SV=1	59.863	2	2.8	3813000	21.8625	115540	0.021
A0A384JCP8	*BCIN_03g05490*	Lactamase_B domain-containing protein OS=*Botryotinia fuckeliana* (strain B05.10) OX=332648 GN=BCIN_03g05490 PE=3 SV=1	35.946	2	4.6	2325300	21.149	110730	0.021
A0A384JG51	*BCIN_05g00180*	Uncharacterized protein OS=*Botryotinia fuckeliana* (strain B05.10) OX=332648 GN=BCIN_05g00180 PE=3 SV=1	66.856	1	1.3	3632000	21.7923	110060	0.02
A0A384JPI1	*Bczuo1*	Bczuo1 OS=*Botryotinia fuckeliana* (strain B05.10) OX=332648 GN=Bczuo1 PE=4 SV=1	50.521	1	2.5	2567200	21.2918	106970	0.02
A0A384J9B9	*BCIN_02g05260*	Coatomer subunit alpha OS=*Botryotinia fuckeliana* (strain B05.10) OX=332648 GN=BCIN_02g05260 PE=4 SV=1	136.5	3	2.4	8019200	22.935	106920	0.02
A0A384J824	*BCIN_02g00230*	Aldo_ket_red domain-containing protein OS=*Botryotinia fuckeliana* (strain B05.10) OX=332648 GN=BCIN_02g00230 PE=4 SV=1	31.989	3	9.2	1868000	20.8331	103780	0.019
A0A384K3G6	*PSD2*	Phosphatidylserine decarboxylase proenzyme 2 OS=*Botryotinia fuckeliana* (strain B05.10) OX=332648 GN=PSD2 PE=3 SV=1	124.67	3	2.5	6294900	22.5858	103200	0.019
A0A384JEP9	*Bcrho1*	Bcrho1 OS=*Botryotinia fuckeliana* (strain B05.10) OX=332648 GN=Bcrho1 PE=4 SV=1	21.555	1	6.8	1228800	20.2288	102400	0.019
A0A384JZI0	*BCIN_12g04690*	Uncharacterized protein OS=*Botryotinia fuckeliana* (strain B05.10) OX=332648 GN=BCIN_12g04690 PE=3 SV=1	48.101	1	1.8	2551800	21.2831	102070	0.019
A0A384JVY1	*Bcerg13*	3-hydroxy-3-methylglutaryl coenzyme A synthase OS=*Botryotinia fuckeliana*	50.347	2	4.1	2465200	21.2333	98609	0.018

		(strain B05.10) OX=332648 GN=Bcerg13 PE=3 SV=1							
A0A384JLP4	*BCIN_07g00520*	60S ribosomal protein L27 OS=*Botryotinia fuckeliana* (strain B05.10) OX=332648 GN=BCIN_07g00520 PE=3 SV=1	15.611	1	8.9	767800	19.5504	95975	0.018
A0A384J4U8	*BCIN_01g03020*	Aldedh domain-containing protein OS=*Botryotinia fuckeliana* (strain B05.10) OX=332648 GN=BCIN_01g03020 PE=4 SV=1	61.483	3	5.6	3627500	21.7905	95460	0.018
A0A384JDG5	*Bctuf1*	Elongation factor Tu OS=*Botryotinia fuckeliana* (strain B05.10) OX=332648 GN=Bctuf1 PE=3 SV=1	49.027	2	4	3091700	21.56	93688	0.017
A0A384JQG8	*BCIN_08g02860*	AB hydrolase-1 domain-containing protein OS=*Botryotinia fuckeliana* (strain B05.10) OX=332648 GN=BCIN_08g02860 PE=3 SV=1	57.095	1	1.5	2698400	21.3637	93050	0.017
A0A384JK75	*BCIN_06g03350*	Uncharacterized protein OS=*Botryotinia fuckeliana* (strain B05.10) OX=332648 GN=BCIN_06g03350 PE=3 SV=1	24.234	1	4.7	1209600	20.2061	93047	0.017
A0A384J855	*BCIN_02g00480*	Uncharacterized protein OS=*Botryotinia fuckeliana* (strain B05.10) OX=332648 GN=BCIN_02g00480 PE=4 SV=1	33.071	1	3.4	1674100	20.675	93005	0.017
A0A384K318	*Bcpre8*	Proteasome subunit alpha type OS=*Botryotinia fuckeliana* (strain B05.10) OX=332648 GN=Bcpre8 PE=3 SV=1	30.075	2	6.8	1378500	20.3947	91901	0.017
A0A384JEL4	*Bcmet17*	Bcmet17 OS=*Botryotinia fuckeliana* (strain B05.10) OX=332648 GN=Bcmet17 PE=3 SV=1	48.869	1	2.2	2387000	21.1868	91807	0.017
A0A384JUZ5	*Bccp4*	Carboxypeptidase OS=*Botryotinia fuckeliana* (strain B05.10) OX=332648 GN=Bccp4 PE=3 SV=1	60.851	1	1.6	2371400	21.1773	91209	0.017
A0A384JE54	*BCIN_04g00980*	Uncharacterized protein OS=*Botryotinia fuckeliana* (strain B05.10) OX=332648 GN=BCIN_04g00980 PE=4 SV=1	113.45	1	0.9	5159800	22.2989	88961	0.017
A0A384K542	*Bcglc3*	Bcglc3 OS=*Botryotinia fuckeliana* (strain B05.10) OX=332648 GN=Bcglc3 PE=4 SV=1	80.146	3	4.1	3715500	21.8251	88464	0.016
A0A384K4K0	*Bccdc12*	Bccdc12 OS=*Botryotinia fuckeliana* (strain B05.10) OX=332648 GN=Bccdc12 PE=3 SV=1	44.354	1	2.8	2007300	20.9368	87276	0.016
A0A384J9F2	*Bcrpt6*	Bcrpt6 OS=*Botryotinia fuckeliana* (strain B05.10) OX=332648 GN=Bcrpt6 PE=3 SV=1	43.328	1	3.3	2396700	21.1926	85595	0.016
A0A384K0P5	*CND6*	CND6 OS=*Botryotinia fuckeliana* (strain B05.10) OX=332648 GN=CND6 PE=4 SV=1	71.444	2	2.3	3461100	21.7228	84417	0.016
A0A384JZV7	*BCIN_12g06020*	Plasma membrane ATPase OS=*Botryotinia fuckeliana* (strain B05.10) OX=332648 GN=BCIN_12g06020 PE=3 SV=1	107.6	2	1.8	4481100	22.0954	82984	0.015
A0A384JIV2	*Bcerg10*	Bcerg10 OS=*Botryotinia fuckeliana* (strain B05.10) OX=332648 GN=Bcerg10	41.631	3	7.8	1625500	20.6325	81277	0.015

		PE=3 SV=1							
A0A384K402	*Bcrts1*	Serine/threonine-protein phosphatase 2A 56 kDa regulatory subunit OS=*Botryotinia fuckeliana* (strain B05.10) OX=332648 GN=Bcrts1 PE=3 SV=1	75.339	1	1	2183400	21.0581	77979	0.014
A0A384JRS7	*Bcfmp10*	Bcfmp10 OS=*Botryotinia fuckeliana* (strain B05.10) OX=332648 GN=Bcfmp10 PE=4 SV=1	35.246	1	3.1	1165500	20.1525	77700	0.014
A0A384JGH8	*BCIN_05g01120*	DLH domain-containing protein OS=*Botryotinia fuckeliana* (strain B05.10) OX=332648 GN=BCIN_05g01120 PE=4 SV=1	30.067	1	3.3	1085400	20.0498	77528	0.014
A0A384K102	*BCIN_13g01450*	DUF2433 domain-containing protein OS=*Botryotinia fuckeliana* (strain B05.10) OX=332648 GN=BCIN_13g01450 PE=4 SV=1	74.992	3	3.7	3098000	21.5629	77449	0.014
A0A384J4E4	*Bchom2*	Bchom2 OS=*Botryotinia fuckeliana* (strain B05.10) OX=332648 GN=Bchom2 PE=4 SV=1	39.476	1	2.2	1387400	20.404	77079	0.014
A0A384JRL1	*BCIN_09g01030*	Uncharacterized protein OS=*Botryotinia fuckeliana* (strain B05.10) OX=332648 GN=BCIN_09g01030 PE=4 SV=1	35.197	1	3.6	1602000	20.6114	76286	0.014
A0A384J6V0	*BCIN_01g09530*	SHSP domain-containing protein OS=*Botryotinia fuckeliana* (strain B05.10) OX=332648 GN=BCIN_01g09530 PE=3 SV=1	27.844	1	4.5	1243000	20.2454	73119	0.014
A0A384J5P0	*BCIN_01g05590*	Uncharacterized protein OS=*Botryotinia fuckeliana* (strain B05.10) OX=332648 GN=BCIN_01g05590 PE=3 SV=1	44.874	2	5.5	1658500	20.6614	69104	0.013
A0A384JET9	*Bcilv5*	Ketol-acid reductoisomerase, mitochondrial OS=*Botryotinia fuckeliana* (strain B05.10) OX=332648 GN=Bcilv5 PE=3 SV=1	44.169	2	4.5	1520400	20.536	66106	0.012
A0A384J9W3	*Bckgd2*	Bckgd2 OS=*Botryotinia fuckeliana* (strain B05.10) OX=332648 GN=Bckgd2 PE=4 SV=1	47.429	1	1.6	1712400	20.7076	65863	0.012
A0A384JI44	*Bcmtc1*	Bcmtc1 OS=*Botryotinia fuckeliana* (strain B05.10) OX=332648 GN=Bcmtc1 PE=4 SV=1	56.76	1	2.3	1763100	20.7497	65300	0.012
A0A384JG48	*Bccdc10*	Bccdc10 OS=*Botryotinia fuckeliana* (strain B05.10) OX=332648 GN=Bccdc10 PE=3 SV=1	39.032	1	2.6	1746900	20.7364	64699	0.012
A0A384JTU3	*Bcbna6*	Nicotinate-nucleotide pyrophosphorylase [carboxylating] OS=*Botryotinia fuckeliana* (strain B05.10) OX=332648 GN=Bcbna6 PE=3 SV=1	31.646	1	2.7	1098600	20.0672	64626	0.012
A0A384JWF9	*BCIN_11g02610*	Obg-like ATPase 1 OS=*Botryotinia fuckeliana* (strain B05.10) OX=332648 GN=BCIN_11g02610 PE=3 SV=1	43.96	1	2.5	1835000	20.8073	63277	0.012
A0A384K5G0	*Bcpgi1*	Glucose-6-phosphate isomerase OS=*Botryotinia fuckeliana* (strain B05.10)	60.949	3	5.8	1892100	20.8516	63069	0.012

		OX=332648 GN=Bcpgi1 PE=3 SV=1							
A0A384J3V4	*BCIN_01g00170*	AA_permease domain-containing protein OS=*Botryotinia fuckeliana* (strain B05.10) OX=332648 GN=BCIN_01g00170 PE=4 SV=1	67.1	1	2.1	1311300	20.3226	62442	0.012
A0A384J9Q1	*BCIN_02g06270*	Uncharacterized protein OS=*Botryotinia fuckeliana* (strain B05.10) OX=332648 GN=BCIN_02g06270 PE=3 SV=1	58.469	1	1.9	2158500	21.0416	61673	0.011
A0A384JGI5	*Bckgd1*	Bckgd1 OS=*Botryotinia fuckeliana* (strain B05.10) OX=332648 GN=Bckgd1 PE=4 SV=1	118.45	2	1.9	3541600	21.756	60027	0.011
A0A384JZ93	*BCIN_12g04670*	AA_TRNA_LIGASE_II domain-containing protein OS=*Botryotinia fuckeliana* (strain B05.10) OX=332648 GN=BCIN_12g04670 PE=3 SV=1	66.123	1	1.9	2096000	20.9992	58223	0.011
A0A384JBE2	*Bccse1*	Bccse1 OS=*Botryotinia fuckeliana* (strain B05.10) OX=332648 GN=Bccse1 PE=4 SV=1	108.76	2	2.1	3201000	21.6101	58199	0.011
A0A384JRG5	*BCIN_09g00600*	Uncharacterized protein OS=*Botryotinia fuckeliana* (strain B05.10) OX=332648 GN=BCIN_09g00600 PE=4 SV=1	32.264	1	2.6	860840	19.7154	53802	0.01
A0A384J7C4	*BCIN_01g09310*	14_3_3 domain-containing protein OS=*Botryotinia fuckeliana* (strain B05.10) OX=332648 GN=BCIN_01g09310 PE=3 SV=1	30.776	2	6.9	1072300	20.0323	53614	0.01
A0A384JQ39	*Bccdc48*	Bccdc48 OS=*Botryotinia fuckeliana* (strain B05.10) OX=332648 GN=Bccdc48 PE=3 SV=1	90.032	2	2.7	2561300	21.2884	53361	0.01
A0A384JGQ2	*Bcglr2*	Glutathione reductase OS=*Botryotinia fuckeliana* (strain B05.10) OX=332648 GN=Bcglr2 PE=3 SV=1	59.639	1	1.6	1402200	20.4193	51933	0.01
A0A384JSI1	*BCIN_09g03810*	DLH domain-containing protein OS=*Botryotinia fuckeliana* (strain B05.10) OX=332648 GN=BCIN_09g03810 PE=4 SV=1	28.15	3	10.6	926030	19.8207	51446	0.01
A0A384JKI4	*BCIN_06g05400*	Uncharacterized protein OS=*Botryotinia fuckeliana* (strain B05.10) OX=332648 GN=BCIN_06g05400 PE=3 SV=1	45.758	1	2.5	1334300	20.3477	51320	0.01
A0A384JBL3	*BCIN_03g01130*	Aldedh domain-containing protein OS=*Botryotinia fuckeliana* (strain B05.10) OX=332648 GN=BCIN_03g01130 PE=3 SV=1	60.749	1	1.6	1958900	20.9016	50227	0.009
A0A384K1L2	*BCIN_13g05580*	PKS_ER domain-containing protein OS=*Botryotinia fuckeliana* (strain B05.10) OX=332648 GN=BCIN_13g05580 PE=3 SV=1	37.411	2	4.8	1090300	20.0563	49561	0.009
A0A384K0M2	*Bcded81*	Bcded81 OS=*Botryotinia fuckeliana* (strain B05.10) OX=332648 GN=Bcded81 PE=4 SV=1	66.013	2	4.1	1475500	20.4928	49184	0.009
A0A384J899	*Bcadk1*	Adenylate kinase OS=*Botryotinia fuckeliana*	29.562	2	6.2	914460	19.8026	48130	0.009

		(strain B05.10) OX=332648 GN=Bcadk1 PE=3 SV=1							
A0A384J3Y3	*BCIN_01g0 0450*	PKS_AT domain-containing protein OS=*Botryotinia fuckeliana* (strain B05.10) OX=332648 GN=BCIN_01g00450 PE=3 SV=1	233.47	3	1.3	5513400	22.3945	44824	0.008
A0A384JH70	*BCIN_05g0 3230*	Uncharacterized protein OS=*Botryotinia fuckeliana* (strain B05.10) OX=332648 GN=BCIN_05g03230 PE=3 SV=1	92.849	1	1.1	2145900	21.0332	44707	0.008
A0A384JM54	*Bcphb2*	Prohibitin OS=*Botryotinia fuckeliana* (strain B05.10) OX=332648 GN=Bcphb2 PE=3 SV=1	33.621	1	3.6	928240	19.8241	44202	0.008
A0A384J5I1	*BCIN_01g0 6470*	Uncharacterized protein OS=*Botryotinia fuckeliana* (strain B05.10) OX=332648 GN=BCIN_01g06470 PE=4 SV=1	77.616	1	1.6	1832100	20.8051	43621	0.008
A0A384JMV1	*Bccmk1*	Bccmk1 OS=*Botryotinia fuckeliana* (strain B05.10) OX=332648 GN=Bccmk1 PE=3 SV=1	45.801	1	2.4	1044300	19.9941	43511	0.008
A0A384K2U8	*Bcdbp5*	Bcdbp5 OS=*Botryotinia fuckeliana* (strain B05.10) OX=332648 GN=Bcdbp5 PE=4 SV=1	51.788	1	1.9	1167800	20.1554	43251	0.008
A0A384K1L5	*BCIN_13g0 5370*	NTP_transferase domain-containing protein OS=*Botryotinia fuckeliana* (strain B05.10) OX=332648 GN=BCIN_13g05370 PE=4 SV=1	48.751	1	2.3	1246400	20.2493	42979	0.008
A0A384J6Q7	*BCIN_01g0 7880*	RRM domain-containing protein OS=*Botryotinia fuckeliana* (strain B05.10) OX=332648 GN=BCIN_01g07880 PE=4 SV=1	27.791	1	4.3	887670	19.7597	42270	0.008
A0A384K1W5	*BCIN_14g0 0480*	Beta-glucosidase OS=*Botryotinia fuckeliana* (strain B05.10) OX=332648 GN=BCIN_14g00480 PE=3 SV=1	91.483	1	1.2	1980400	20.9174	42137	0.008
A0A384K3D6	*Bcphb1*	Prohibitin OS=*Botryotinia fuckeliana* (strain B05.10) OX=332648 GN=Bcphb1 PE=3 SV=1	30.737	1	4.7	788750	19.5892	41513	0.008
A0A384J7Y1	*Bcgdb1*	Bcgdb1 OS=*Botryotinia fuckeliana* (strain B05.10) OX=332648 GN=Bcgdb1 PE=4 SV=1	174.37	1	0.8	3781900	21.8507	40666	0.008
A0A384JMJ6	*BCIN_07g0 2940*	Uncharacterized protein OS=*Botryotinia fuckeliana* (strain B05.10) OX=332648 GN=BCIN_07g02940 PE=4 SV=1	37.576	1	3.8	483810	18.8841	40318	0.007
A0A384K330	*BCIN_14g0 3970*	1,3-beta-glucanosyltransferase OS=*Botryotinia fuckeliana* (strain B05.10) OX=332648 GN=BCIN_14g03970 PE=3 SV=1	47.687	1	2.4	673310	19.3609	39606	0.007
A0A384J9S5	*Bcths1*	Bcths1 OS=*Botryotinia fuckeliana* (strain B05.10) OX=332648 GN=Bcths1 PE=3 SV=1	89.282	2	2.4	2130900	21.023	39461	0.007
A0A384K617	*Bccrn1*	Coronin OS=*Botryotinia fuckeliana* (strain B05.10) OX=332648 GN=Bccrn1 PE=3 SV=1	66.405	1	1.3	1537300	20.552	39417	0.007

A0A384JG93	*BCIN_05g0 0580*	Uncharacterized protein OS=*Botryotinia fuckeliana* (strain B05.10) OX=332648 GN=BCIN_05g00580 PE=4 SV=1	57.317	1	2.9	1046000	19.9965	38740	0.007
A0A384JSJ6	*Bcscl1*	Proteasome subunit alpha type OS=*Botryotinia fuckeliana* (strain B05.10) OX=332648 GN=Bcscl1 PE=3 SV=1	27.746	2	6.7	687430	19.3909	38191	0.007
A0A384JD73	*Bccat2*	Bccat2 OS=*Botryotinia fuckeliana* (strain B05.10) OX=332648 GN=Bccat2 PE=3 SV=1	72.539	1	1.3	1565500	20.5782	38184	0.007
A0A384JST8	*BCIN_09g0 4160*	Uncharacterized protein OS=*Botryotinia fuckeliana* (strain B05.10) OX=332648 GN=BCIN_09g04160 PE=4 SV=1	80.87	1	1.5	1314800	20.3264	37565	0.007
A0A384JD53	*BCIN_03g0 4810*	Polyadenylate-binding protein OS=*Botryotinia fuckeliana* (strain B05.10) OX=332648 GN=BCIN_03g04810 PE=3 SV=1	85.897	1	1.6	1426600	20.4441	37543	0.007
A0A384JAD8	*Bctps1*	Trehalose-6-phosphate synthase OS=*Botryotinia fuckeliana* (strain B05.10) OX=332648 GN=Bctps1 PE=3 SV=1	59.349	1	1.7	917010	19.8066	36681	0.007
A0A384JR47	*Bcagc1*	Bcagc1 OS=*Botryotinia fuckeliana* (strain B05.10) OX=332648 GN=Bcagc1 PE=3 SV=1	78.389	2	2.4	1662600	20.665	36144	0.007
A0A384JX00	*BCIN_11g0 3520*	S-adenosylmethionine synthase OS=*Botryotinia fuckeliana* (strain B05.10) OX=332648 GN=BCIN_11g03520 PE=3 SV=1	43.121	1	2.5	635390	19.2773	35300	0.007
A0A384K0L0	*BCIN_13g0 1350*	Complex1_30kDa domain-containing protein OS=*Botryotinia fuckeliana* (strain B05.10) OX=332648 GN=BCIN_13g01350 PE=3 SV=1	33.253	1	3.8	629040	19.2628	34947	0.006
A0A384J8V3	*Bcggc1*	Bcggc1 OS=*Botryotinia fuckeliana* (strain B05.10) OX=332648 GN=Bcggc1 PE=3 SV=1	32.945	1	2.6	689810	19.3958	32848	0.006
A0A384JW57	*BCIN_11g0 1050*	Uncharacterized protein OS=*Botryotinia fuckeliana* (strain B05.10) OX=332648 GN=BCIN_11g01050 PE=4 SV=1	53.555	1	2.1	829170	19.6613	30710	0.006
A0A384JA99	*BCIN_02g0 8330*	Uncharacterized protein OS=*Botryotinia fuckeliana* (strain B05.10) OX=332648 GN=BCIN_02g08330 PE=4 SV=1	103.78	1	1	1144000	20.1257	30105	0.006
A0A384JZB5	*BCIN_12g0 4860*	Uncharacterized protein OS=*Botryotinia fuckeliana* (strain B05.10) OX=332648 GN=BCIN_12g04860 PE=3 SV=1	246.57	1	0.4	3693800	21.8167	29316	0.005
A0A384JMM8	*BCIN_07g0 3810*	Uncharacterized protein OS=*Botryotinia fuckeliana* (strain B05.10) OX=332648 GN=BCIN_07g03810 PE=3 SV=1	77.792	1	1	1307700	20.3186	26688	0.005
A0A384JB13	*BCIN_02g0 7690*	Uncharacterized protein OS=*Botryotinia fuckeliana* (strain B05.10) OX=332648 GN=BCIN_02g07690 PE=4 SV=1	35.17	1	3.1	503200	18.9408	25160	0.005

A0A384K1A1	*CND16*	CND16 OS=*Botryotinia fuckeliana* (strain B05.10) OX=332648 GN=CND16 PE=4 SV=1	52.76	1	2.3	703430	19.424	25123	0.005
A0A384JXJ0	*BCIN_11g05910*	Dipeptidyl peptidase 3 OS=*Botryotinia fuckeliana* (strain B05.10) OX=332648 GN=BCIN_11g05910 PE=3 SV=1	85.57	1	1.3	1182100	20.1729	24628	0.005
A0A384JV52	*Bcnop1*	Bcnop1 OS=*Botryotinia fuckeliana* (strain B05.10) OX=332648 GN=Bcnop1 PE=3 SV=1	33.635	1	4	465210	18.8275	24484	0.005
A0A384JQ98	*BCIN_08g02390*	Uncharacterized protein OS=*Botryotinia fuckeliana* (strain B05.10) OX=332648 GN=BCIN_08g02390 PE=4 SV=1	60.221	2	3.7	489270	18.9003	24463	0.005
A0A384K577	*Bcglt1*	Bcglt1 OS=*Botryotinia fuckeliana* (strain B05.10) OX=332648 GN=Bcglt1 PE=4 SV=1	236.28	1	0.5	2914300	21.4747	24286	0.005
A0A384JRB3	*BCIN_08g05220*	Triosephosphate isomerase OS=*Botryotinia fuckeliana* (strain B05.10) OX=332648 GN=BCIN_08g05220 PE=3 SV=1	27.012	1	4	412190	18.6529	24247	0.004
A0A384JRS9	*BCIN_09g00980*	Isocitrate dehydrogenase [NADP] OS=*Botryotinia fuckeliana* (strain B05.10) OX=332648 GN=BCIN_09g00980 PE=3 SV=1	50.36	1	2.2	737330	19.492	23785	0.004
A0A384JLW6	*BCIN_07g01890*	Pyruvate dehydrogenase E1 component subunit alpha OS=*Botryotinia fuckeliana* (strain B05.10) OX=332648 GN=BCIN_07g01890 PE=4 SV=1	45.478	1	2.4	633570	19.2731	23465	0.004
A0A384JCR3	*BCIN_03g04790*	Aldo_ket_red domain-containing protein OS=*Botryotinia fuckeliana* (strain B05.10) OX=332648 GN=BCIN_03g04790 PE=4 SV=1	35.529	1	2.8	523650	18.9982	22767	0.004
A0A384K5K4	*Bcsak1*	Mitogen-activated protein kinase OS=*Botryotinia fuckeliana* (strain B05.10) OX=332648 GN=Bcsak1 PE=3 SV=1	39.391	1	3.2	434500	18.729	21725	0.004
A0A384JDD5	*BCIN_03g06500*	Aldo_ket_red domain-containing protein OS=*Botryotinia fuckeliana* (strain B05.10) OX=332648 GN=BCIN_03g06500 PE=4 SV=1	39.572	1	3.1	489360	18.9005	21277	0.004
A0A384JMY5	*Bcidh2*	Isocitrate dehydrogenase [NAD] subunit, mitochondrial OS=*Botryotinia fuckeliana* (strain B05.10) OX=332648 GN=Bcidh2 PE=3 SV=1	41.61	1	2.3	505400	18.9471	21058	0.004
A0A384JGD5	*Bcpan1*	Bcpan1 OS=*Botryotinia fuckeliana* (strain B05.10) OX=332648 GN=Bcpan1 PE=4 SV=1	155.9	1	0.6	1369300	20.385	20747	0.004
A0A384JA92	*BCIN_02g06950*	Uncharacterized protein OS=*Botryotinia fuckeliana* (strain B05.10) OX=332648 GN=BCIN_02g06950 PE=3 SV=1	33.146	1	3	331380	18.3381	20712	0.004
A0A384JD07	*Bcnip1*	Eukaryotic translation initiation factor 3 subunit C OS=*Botryotinia fuckeliana* (strain B05.10) OX=332648	97.367	2	2.3	1083400	20.0471	19698	0.004

		GN=Bcnip1 PE=3 SV=1							
A0A384JEA2	*Bclys2*	Bclys2 OS=*Botryotinia fuckeliana* (strain B05.10) OX=332648 GN=Bclys2 PE=4 SV=1	128.3	1	0.9	1303600	20.3141	19457	0.004
A0A384JL58	*BCIN_06g05380*	Kinesin-like protein OS=*Botryotinia fuckeliana* (strain B05.10) OX=332648 GN=BCIN_06g05380 PE=3 SV=1	102.78	2	2.5	1106400	20.0774	17561	0.003
A0A384J5E9	*BCIN_01g05060*	Aminopeptidase OS=*Botryotinia fuckeliana* (strain B05.10) OX=332648 GN=BCIN_01g05060 PE=3 SV=1	99.229	1	1.5	1004500	19.938	17319	0.003
A0A384J8K9	*BCIN_02g01720*	Phospholipase OS=*Botryotinia fuckeliana* (strain B05.10) OX=332648 GN=BCIN_02g01720 PE=3 SV=1	97.916	1	1.3	826870	19.6573	16537	0.003
A0A384JBN5	*Bclhs1*	Bclhs1 OS=*Botryotinia fuckeliana* (strain B05.10) OX=332648 GN=Bclhs1 PE=4 SV=1	112.75	1	1	1040000	19.9882	16000	0.003
A0A384JFN5	*BCIN_04g05860*	Uncharacterized protein OS=*Botryotinia fuckeliana* (strain B05.10) OX=332648 GN=BCIN_04g05860 PE=4 SV=1	26.13	1	3	246540	17.9115	13697	0.003
A0A384JR08	*BCIN_08g06300*	ATP-dependent 6-phosphofructokinase OS=*Botryotinia fuckeliana* (strain B05.10) OX=332648 GN=BCIN_08g06300 PE=3 SV=1	86.569	2	2.4	483310	18.8826	11788	0.002
A0A384J4X7	*BCIN_01g03440*	Uncharacterized protein OS=*Botryotinia fuckeliana* (strain B05.10) OX=332648 GN=BCIN_01g03440 PE=3 SV=1	124.3	1	1.1	772440	19.5591	11704	0.002
A0A384JFH9	*BCIN_04g05060*	Pribosyltran_N domain-containing protein OS=*Botryotinia fuckeliana* (strain B05.10) OX=332648 GN=BCIN_04g05060 PE=4 SV=1	34.771	1	3.1	209390	17.6758	10469	0.002
A0A384J9T2	*Bcrpn9*	Bcrpn9 OS=*Botryotinia fuckeliana* (strain B05.10) OX=332648 GN=Bcrpn9 PE=4 SV=1	43.163	1	2.4	242210	17.8859	9688.3	0.002
A0A384JNT7	*BCIN_07g06960*	Uncharacterized protein OS=*Botryotinia fuckeliana* (strain B05.10) OX=332648 GN=BCIN_07g06960 PE=4 SV=1	253.58	3	1.3	1404100	20.4212	9486.9	0.002
A0A384K3U2	*BCIN_15g00140*	Fumarate reductase OS=*Botryotinia fuckeliana* (strain B05.10) OX=332648 GN=BCIN_15g00140 PE=3 SV=1	53.333	1	1.6	282560	18.1082	9114.9	0.002
A0A384JA51	*BCIN_02g05890*	CRAL-TRIO domain-containing protein OS=*Botryotinia fuckeliana* (strain B05.10) OX=332648 GN=BCIN_02g05890 PE=4 SV=1	72.58	1	1.1	327840	18.3226	8196	0.002
A0A384J6S5	*Bcchc1*	Clathrin heavy chain OS=*Botryotinia fuckeliana* (strain B05.10) OX=332648 GN=Bcchc1 PE=3 SV=1	189.4	1	0.7	776180	19.566	7840.2	0.001
A0A384J5D0	*Bcmet3*	Sulfate adenylyltransferase OS=*Botryotinia fuckeliana* (strain B05.10) OX=332648 GN=Bcmet3 PE=3 SV=1	64.035	1	2.3	192280	17.5528	5493.8	0.001

A0A384J7Z9	*Bcrot2*	Bcrot2 OS=*Botryotinia fuckeliana* (strain B05.10) OX=332648 GN=Bcrot2 PE=3 SV=1	109.02	1	0.8	260390	17.9903	4912.9	0.001
A0A384JTZ6	*BCIN_10g00110*	6-phosphogluconolactonase OS=*Botryotinia fuckeliana* (strain B05.10) OX=332648 GN=BCIN_10g00110 PE=3 SV=1	28.244	1	4.2	63602	15.9568	4240.1	0.001
A0A384J5L7	*BCIN_01g05450*	Uncharacterized protein OS=*Botryotinia fuckeliana* (strain B05.10) OX=332648 GN=BCIN_01g05450 PE=4 SV=1	101.4	1	0.9	0	NA	0	0
A0A384JA39	*BCIN_02g07780*	Uncharacterized protein OS=*Botryotinia fuckeliana* (strain B05.10) OX=332648 GN=BCIN_02g07780 PE=4 SV=1	49.858	1	1.3	0	NA	0	0
A0A384JBR1	*Bclga1*	Bclga1 OS=*Botryotinia fuckeliana* (strain B05.10) OX=332648 GN=Bclga1 PE=3 SV=1	34.904	2	6.2	0	NA	0	0
A0A384JFX6	*Bcvma2*	Vacuolar proton pump subunit B OS=*Botryotinia fuckeliana* (strain B05.10) OX=332648 GN=Bcvma2 PE=3 SV=1	57.599	1	1.9	0	NA	0	0
A0A384JKX7	*Bcpdi1*	Protein disulfide-isomerase OS=*Botryotinia fuckeliana* (strain B05.10) OX=332648 GN=Bcpdi1 PE=3 SV=1	57.485	2	2.6	0	NA	0	0
A0A384JNF8	*BCIN_07g06220*	Uncharacterized protein OS=*Botryotinia fuckeliana* (strain B05.10) OX=332648 GN=BCIN_07g06220 PE=3 SV=1	73.476	1	1	0	NA	0	0
A0A384JSR0	*BCIN_09g02790*	Acetyl-coenzyme A synthetase OS=*Botryotinia fuckeliana* (strain B05.10) OX=332648 GN=BCIN_09g02790 PE=3 SV=1	73.978	1	1.5	0	NA	0	0
A0A384JUR8	*BCIN_10g02770*	Uncharacterized protein OS=*Botryotinia fuckeliana* (strain B05.10) OX=332648 GN=BCIN_10g02770 PE=4 SV=1	61.262	1	1.7	0	NA	0	0
A0A384JYB8	*BCIN_12g01170*	UDPG_MGDP_dh_C domain-containing protein OS=*Botryotinia fuckeliana* (strain B05.10) OX=332648 GN=BCIN_12g01170 PE=4 SV=1	68.696	1	1.6	0	NA	0	0
A0A384K2B8	*Bcaif1*	Bcaif1 OS=*Botryotinia fuckeliana* (strain B05.10) OX=332648 GN=Bcaif1 PE=4 SV=1	63.148	2	2.9	0	NA	0	0
A0A384K2K4	*BCIN_14g02290*	Uncharacterized protein OS=*Botryotinia fuckeliana* (strain B05.10) OX=332648 GN=BCIN_14g02290 PE=4 SV=1	144.44	1	0.8	0	NA	0	0
A0A384K5R0	*Bcifa38*	Very-long-chain 3-oxoacyl-CoA reductase OS=*Botryotinia fuckeliana* (strain B05.10) OX=332648 GN=Bcifa38 PE=3 SV=1	35.874	1	2.7	0	NA	0	0
A0A384K801	*BCIN_16g04810*	Ribosomal_L28e domain-containing protein OS=*Botryotinia fuckeliana* (strain B05.10) OX=332648 GN=BCIN_16g04810 PE=4 SV=1	16.008	1	7.3	0	NA	0	0

Table S2. The identified potential targets of Z24 in the B2 band.

Accession	Gene	Description	Mw (kDa)	Peptides B2	Sequence coverage B2 [%]	Intensity B2	log2 (B2)	iBAQ B2	iBAQ B2 [%]
A0A384K7U0	*BCIN_16g03380*	AUncharacterized protein OS=*Botryotinia fuckeliana* (strain B05.10) OX=332648 GN=BCIN_16g03380 PE=4 SV=1	28.355	8	21.8	1.66E+09	30.6326	87614000	15.297
A0A384JBI6	*BCIN_03g01010*	Uncharacterized protein OS=*Botryotinia fuckeliana* (strain B05.10) OX=332648 GN=BCIN_03g01010 PE=3 SV=1	33.957	13	29.7	1.66E+09	30.6282	87348000	15.25
A0A384JTH0	*Bcef1a*	Elongation factor 1-alpha OS=*Botryotinia fuckeliana* (strain B05.10) OX=332648 GN=Bcef1a PE=3 SV=1	49.983	12	19.3	5.64E+08	29.0721	21707000	3.79
A0A384JRB3	*BCIN_08g05220*	Triosephosphate isomerase OS=*Botryotinia fuckeliana* (strain B05.10) OX=332648 GN=BCIN_08g05220 PE=3 SV=1	27.012	8	22.7	3.62E+08	28.4302	21276000	3.715
A0A384J440	*Bcfpr2*	Peptidylprolyl isomerase OS=*Botryotinia fuckeliana* (strain B05.10) OX=332648 GN=Bcfpr2 PE=4 SV=1	21.147	3	13.5	2.43E+08	27.857	15193000	2.653
A0A384JM89	*BCIN_07g01150*	Ubiquitin-like domain-containing protein OS=*Botryotinia fuckeliana* (strain B05.10) OX=332648 GN=BCIN_07g01150 PE=4 SV=1	14.566	4	21.9	1.23E+08	26.876	13684000	2.389
A0A384K1A0	*BCIN_13g04420*	Histone H4 OS=*Botryotinia fuckeliana* (strain B05.10) OX=332648 GN=BCIN_13g04420 PE=3 SV=1	11.356	1	9.7	67373000	26.0057	11229000	1.961
A0A384J453	*BCIN_01g00980*	S5 DRBM domain-containing protein OS=*Botryotinia fuckeliana* (strain B05.10) OX=332648 GN=BCIN_01g00980 PE=3 SV=1	28.37	6	14.1	1.78E+08	27.4101	10491000	1.832
A0A384JXF3	*BCIN_11g02490*	Uncharacterized protein OS=*Botryotinia fuckeliana* (strain B05.10) OX=332648 GN=BCIN_11g02490 PE=4 SV=1	60.028	10	19.5	3.34E+08	28.3162	9549100	1.667
A0A384JCX3	*RPS1*	40S ribosomal protein S1 OS=*Botryotinia fuckeliana* (strain B05.10) OX=332648 GN=RPS1 PE=3 SV=1	29.246	7	17.2	1.58E+08	27.2317	9270700	1.619
A0A384JLG3	*Bcpup1*	Bcpup1 OS=*Botryotinia fuckeliana* (strain B05.10) OX=332648 GN=Bcpup1 PE=4 SV=1	29.331	4	15	92028000	26.4556	9202800	1.607
A0A384K4H3	*Bcpre6*	Proteasome subunit alpha type	29.154	5	11.6	1.73E+08	27.3691	9123500	1.593

		OS=*Botryotinia fuckeliana* (strain B05.10) OX=332648 GN=Bcpre6 PE=3 SV=1							
A0A384J4J0	*BCIN_01g02200*	S4 RNA-binding domain-containing protein OS=*Botryotinia fuckeliana* (strain B05.10) OX=332648 GN=BCIN_01g02200 PE=3 SV=1	22.279	8	28.3	1.22E+08	26.8597	8698700	1.519
A0A384J809	*BCIN_01g11220*	Uncharacterized protein OS=*Botryotinia fuckeliana* (strain B05.10) OX=332648 GN=BCIN_01g11220 PE=3 SV=1	31.929	1	3.1	1.02E+08	26.6098	8534200	1.49
A0A384J4F1	*BCIN_01g01800*	40S ribosomal protein S4 OS=*Botryotinia fuckeliana* (strain B05.10) OX=332648 GN=BCIN_01g01800 PE=3 SV=1	29.332	10	29.8	1.95E+08	27.5375	8469900	1.479
A0A384JSJ6	*Bcscl1*	Proteasome subunit alpha type OS=*Botryotinia fuckeliana* (strain B05.10) OX=332648 GN=Bcscl1 PE=3 SV=1	27.746	6	22	1.32E+08	26.9763	7335200	1.281
A0A384JBE3	*BCIN_03g01820*	Ribosomal protein OS=*Botryotinia fuckeliana* (strain B05.10) OX=332648 GN=BCIN_03g01820 PE=3 SV=1	24.178	2	9.7	88924000	26.4061	6840300	1.194
A0A384JFX3	*BCIN_04g05190*	40S ribosomal protein S8 OS=*Botryotinia fuckeliana* (strain B05.10) OX=332648 GN=BCIN_04g05190 PE=3 SV=1	23.17	4	18.6	89358000	26.4131	5957200	1.04
A0A384JD42	*BCIN_03g07630*	Uncharacterized protein OS=*Botryotinia fuckeliana* (strain B05.10) OX=332648 GN=BCIN_03g07630 PE=4 SV=1	28.62	5	15.7	82115000	26.2911	5865300	1.024
A0A384JFG3	*BCIN_04g05070*	40S ribosomal protein S6 OS=*Botryotinia fuckeliana* (strain B05.10) OX=332648 GN=BCIN_04g05070 PE=3 SV=1	27.201	4	11.3	68701000	26.0338	5725100	1
A0A384K7X4	*Bcfdh1*	Formate dehydrogenase OS=*Botryotinia fuckeliana* (strain B05.10) OX=332648 GN=Bcfdh1 PE=3 SV=1	40.851	5	9.4	1.17E+08	26.8023	5572800	0.973
A0A384K3Z3	*Bcatp1*	ATP synthase subunit alpha OS=*Botryotinia fuckeliana* (strain B05.10) OX=332648 GN=Bcatp1 PE=3 SV=1	59.85	9	15.3	1.9E+08	27.5005	5425000	0.947
A0A384JCX7	*BCIN_03g06830*	Uricase OS=*Botryotinia fuckeliana* (strain B05.10) OX=332648 GN=BCIN_03g06830 PE=3 SV=1	33.584	6	14.6	90417000	26.4301	4758800	0.831
A0A384J9H1	*BCIN_02g04220*	Ribos_L4_asso_C domain-containing protein OS=*Botryotinia fuckeliana* (strain B05.10) OX=332648	40.04	10	24.1	1.18E+08	26.8097	4705300	0.822

		GN=BCIN_02g04220 PE=4 SV=1							
A0A384JB76	*Bcmir1*	Bcmir1 OS=*Botryotinia fuckeliana* (strain B05.10) OX=332648 GN=Bcmir1 PE=3 SV=1	33.612	7	16.8	89038000	26.4079	4686200	0.818
A0A384JD98	*BCIN_03g06530*	Ribosomal_L7Ae domain-containing protein OS=*Botryotinia fuckeliana* (strain B05.10) OX=332648 GN=BCIN_03g06530 PE=4 SV=1	28.897	8	24.6	73984000	26.1407	4624000	0.807
A0A384JU32	*BCIN_09g06480*	Uncharacterized protein OS=*Botryotinia fuckeliana* (strain B05.10) OX=332648 GN=BCIN_09g06480 PE=4 SV=1	17.478	4	17.4	36675000	25.1283	4584400	0.8
A0A384JVB5	*Bcrps3*	Bcrps3 OS=*Botryotinia fuckeliana* (strain B05.10) OX=332648 GN=Bcrps3 PE=3 SV=1	28.512	8	27.4	90183000	26.4264	4509200	0.787
A0A384JLY6	*BCIN_07g00300*	Hemerythrin domain-containing protein OS=*Botryotinia fuckeliana* (strain B05.10) OX=332648 GN=BCIN_07g00300 PE=4 SV=1	28.964	4	17.4	84030000	26.3244	4422600	0.772
A0A384JNS6	*Bcpup2*	Proteasome subunit alpha type OS=*Botryotinia fuckeliana* (strain B05.10) OX=332648 GN=Bcpup2 PE=3 SV=1	26.844	3	11.8	63543000	25.9212	3971400	0.693
A0A384JTD7	*BCIN_09g04320*	Uncharacterized protein OS=*Botryotinia fuckeliana* (strain B05.10) OX=332648 GN=BCIN_09g04320 PE=3 SV=1	42.839	1	2	47212000	25.4927	3934300	0.687
A0A384JFE4	*Bcnmt1*	Bcnmt1 OS=*Botryotinia fuckeliana* (strain B05.10) OX=332648 GN=Bcnmt1 PE=4 SV=1	38.422	8	19.3	77633000	26.2102	3696800	0.645
A0A384J6N9	*Bcprx4*	Bcprx4 OS=*Botryotinia fuckeliana* (strain B05.10) OX=332648 GN=Bcprx4 PE=4 SV=1	25.578	4	15.7	53888000	25.6835	3368000	0.588
A0A384JEF1	*BCIN_04g00540*	Ribosomal protein L19 OS=*Botryotinia fuckeliana* (strain B05.10) OX=332648 GN=BCIN_04g00540 PE=3 SV=1	22.464	5	17.5	39799000	25.2462	3316500	0.579
A0A384J7C4	*BCIN_01g09310*	14_3_3 domain-containing protein OS=*Botryotinia fuckeliana* (strain B05.10) OX=332648 GN=BCIN_01g09310 PE=3 SV=1	30.776	5	11.6	65362000	25.9619	3268100	0.571
A0A384JTZ6	*BCIN_10g00110*	6-phosphogluconolactonase OS=*Botryotinia fuckeliana* (strain B05.10) OX=332648 GN=BCIN_10g00110 PE=3 SV=1	28.244	3	8	47646000	25.5059	3176400	0.555

A0A384JFP6	*BCIN_04g04500*	Uncharacterized protein OS=*Botryotinia fuckeliana* (strain B05.10) OX=332648 GN=BCIN_04g04500 PE=4 SV=1	29.981	2	7.4	63036000	25.9097	3151800	0.55
A0A384J5V2	*Bcenol1*	Bcenol1 OS=*Botryotinia fuckeliana* (strain B05.10) OX=332648 GN=Bcenol1 PE=3 SV=1	47.154	4	9.6	79923000	26.2521	3074000	0.537
A0A384JBU2	*BCIN_03g03360*	Transaldolase OS=*Botryotinia fuckeliana* (strain B05.10) OX=332648 GN=BCIN_03g03360 PE=3 SV=1	35.402	4	13.3	65120000	25.9566	2960000	0.517
A0A384K2H3	*BCIN_14g01980*	Transketolase OS=*Botryotinia fuckeliana* (strain B05.10) OX=332648 GN=BCIN_14g01980 PE=3 SV=1	74.965	12	14.5	96472000	26.5236	2756300	0.481
A0A384JRW5	*Bcpre9*	Proteasome subunit alpha type OS=*Botryotinia fuckeliana* (strain B05.10) OX=332648 GN=Bcpre9 PE=3 SV=1	28.367	5	19.5	43726000	25.382	2572100	0.449
A0A384JBQ4	*BCIN_03g02530*	Ribosomal_L2_C domain-containing protein OS=*Botryotinia fuckeliana* (strain B05.10) OX=332648 GN=BCIN_03g02530 PE=4 SV=1	27.405	5	16.1	35697000	25.0893	2549800	0.445
A0A384JNK6	*Bcthi4*	Thiamine thiazole synthase OS=*Botryotinia fuckeliana* (strain B05.10) OX=332648 GN=Bcthi4 PE=3 SV=1	34.969	7	18.3	43225000	25.3654	2542700	0.444
A0A384JE84	*BCIN_04g01180*	Ribosomal_L18e/L15P domain-containing protein OS=*Botryotinia fuckeliana* (strain B05.10) OX=332648 GN=BCIN_04g01180 PE=4 SV=1	20.735	3	15.3	32266000	24.9435	2482000	0.433
A0A384K318	*Bcpre8*	Proteasome subunit alpha type OS=*Botryotinia fuckeliana* (strain B05.10) OX=332648 GN=Bcpre8 PE=3 SV=1	30.075	4	10.4	34681000	25.0476	2312100	0.404
A0A384J7B7	*Bcpre7*	Bcpre7 OS=*Botryotinia fuckeliana* (strain B05.10) OX=332648 GN=Bcpre7 PE=4 SV=1	29.034	3	12.9	30039000	24.8403	2310700	0.403
A0A384JJ19	*BCIN_06g00380*	Ribosomal_S17_N domain-containing protein OS=*Botryotinia fuckeliana* (strain B05.10) OX=332648 GN=BCIN_06g00380 PE=3 SV=1	18.395	5	33.5	20543000	24.2921	2282600	0.399
A0A384JU36	*BCIN_10g01260*	Uncharacterized protein OS=*Botryotinia fuckeliana* (strain B05.10) OX=332648 GN=BCIN_10g01260 PE=3 SV=1	23.031	3	12.4	38374000	25.1936	2257300	0.394
A0A384JY85	*BCIN_12g00910*	Uncharacterized protein OS=*Botryotinia fuckeliana* (strain B05.10)	17.091	2	12.7	11184000	23.4149	2236800	0.391

		OX=332648 GN=BCIN_12g00910 PE=4 SV=1							
A0A384K2W2	*Bcrpl3*	Bcrpl3 OS=*Botryotinia fuckeliana* (strain B05.10) OX=332648 GN=Bcrpl3 PE=3 SV=1	44.251	6	9.7	53366000	25.6694	2223600	0.388
A0A384J824	*BCIN_02g00230*	Aldo_ket_red domain-containing protein OS=*Botryotinia fuckeliana* (strain B05.10) OX=332648 GN=BCIN_02g00230 PE=4 SV=1	31.989	5	17.1	39670000	25.2415	2203900	0.385
A0A384JSI1	*BCIN_09g03810*	DLH domain-containing protein OS=*Botryotinia fuckeliana* (strain B05.10) OX=332648 GN=BCIN_09g03810 PE=4 SV=1	28.15	5	14.6	39568000	25.2378	2198200	0.384
A0A384JXH5	*Bcsah1*	Adenosylhomocysteinase OS=*Botryotinia fuckeliana* (strain B05.10) OX=332648 GN=Bcsah1 PE=3 SV=1	48.849	7	13.4	50766000	25.5974	2115200	0.369
A0A384J4J9	*BCIN_01g02210*	Uncharacterized protein OS=*Botryotinia fuckeliana* (strain B05.10) OX=332648 GN=BCIN_01g02210 PE=4 SV=1	18.296	2	9.4	22322000	24.412	2029300	0.354
A0A384K4Y8	*Bcgph1*	Alpha-1,4 glucan phosphorylase OS=*Botryotinia fuckeliana* (strain B05.10) OX=332648 GN=Bcgph1 PE=3 SV=1	99.74	7	7.4	1.17E+08	26.8039	2020100	0.353
A0A384JCX1	*BCIN_03g06600*	Uncharacterized protein OS=*Botryotinia fuckeliana* (strain B05.10) OX=332648 GN=BCIN_03g06600 PE=3 SV=1	68.669	8	10.8	76721000	26.1931	2019000	0.353
A0A384K4A0	*Bcatp2*	ATP synthase subunit beta OS=*Botryotinia fuckeliana* (strain B05.10) OX=332648 GN=Bcatp2 PE=3 SV=1	54.546	5	9.2	59922000	25.8366	1997400	0.349
A0A384JBY4	*BCIN_03g03440*	Uncharacterized protein OS=*Botryotinia fuckeliana* (strain B05.10) OX=332648 GN=BCIN_03g03440 PE=4 SV=1	26.998	2	7.7	23602000	24.4924	1966800	0.343
A0A384JLV2	*BCIN_07g01540*	Tr-type G domain-containing protein OS=*Botryotinia fuckeliana* (strain B05.10) OX=332648 GN=BCIN_07g01540 PE=4 SV=1	93.459	7	8.8	1.1E+08	26.7065	1955500	0.341
A0A384J7A7	*Bcpsd*	Bcpsd OS=*Botryotinia fuckeliana* (strain B05.10) OX=332648 GN=Bcpsd PE=4 SV=1	46.447	6	8.8	50503000	25.5899	1942400	0.339
A0A384JCN3	*BCIN_03g05840*	Uncharacterized protein OS=*Botryotinia fuckeliana* (strain B05.10) OX=332648 GN=BCIN_03g05840 PE=3 SV=1	30.931	3	10.8	31915000	24.9277	1773100	0.31

A0A384J564	*Bcugp1*	UTP--glucose-1-phosphate uridylyltransferase OS=*Botryotinia fuckeliana* (strain B05.10) OX=332648 GN=Bcugp1 PE=3 SV=1	58.609	7	11.8	52219000	25.6381	1535900	0.268
A0A384J8S2	*BCIN_02g02750*	Citrate synthase OS=*Botryotinia fuckeliana* (strain B05.10) OX=332648 GN=BCIN_02g02750 PE=3 SV=1	52.059	3	5.5	40753000	25.2804	1509400	0.264
A0A384JTU5	*Bcrpl10*	Bcrpl10 OS=*Botryotinia fuckeliana* (strain B05.10) OX=332648 GN=Bcrpl10 PE=4 SV=1	25.372	1	4.1	22636000	24.4321	1509100	0.263
A0A384K3D6	*Bcphb1*	Prohibitin OS=*Botryotinia fuckeliana* (strain B05.10) OX=332648 GN=Bcphb1 PE=3 SV=1	30.737	2	7.9	28411000	24.7599	1495300	0.261
A0A384J759	*BCIN_01g09350*	60S ribosomal protein L36 OS=*Botryotinia fuckeliana* (strain B05.10) OX=332648 GN=BCIN_01g09350 PE=3 SV=1	11.771	2	17.9	7361400	22.8115	1472300	0.257
A0A384JMC8	*BCIN_07g02610*	6-phosphogluconate dehydrogenase, decarboxylating OS=*Botryotinia fuckeliana* (strain B05.10) OX=332648 GN=BCIN_07g02610 PE=3 SV=1	54.71	5	10.7	42449000	25.3392	1463700	0.256
A0A384JAG9	*Bcshm2*	Serine hydroxymethyltransferase OS=*Botryotinia fuckeliana* (strain B05.10) OX=332648 GN=Bcshm2 PE=3 SV=1	52.696	5	9.4	38827000	25.2106	1338900	0.234
A0A384J7J8	*BCIN_01g10140*	Uncharacterized protein OS=*Botryotinia fuckeliana* (strain B05.10) OX=332648 GN=BCIN_01g10140 PE=4 SV=1	29.434	2	8.4	20049000	24.257	1336600	0.233
A0A384J9Y2	*BCIN_02g06580*	Uncharacterized protein OS=*Botryotinia fuckeliana* (strain B05.10) OX=332648 GN=BCIN_02g06580 PE=3 SV=1	62.692	4	7.5	41183000	25.2955	1328500	0.232
A0A384K2T7	*Bcgbl1*	Bcgbl1 OS=*Botryotinia fuckeliana* (strain B05.10) OX=332648 GN=Bcgbl1 PE=4 SV=1	34.931	2	3.2	25450000	24.6012	1272500	0.222
A0A384J4W3	*Bcipp1*	Bcipp1 OS=*Botryotinia fuckeliana* (strain B05.10) OX=332648 GN=Bcipp1 PE=4 SV=1	32.741	3	9.3	21437000	24.3536	1261000	0.22
A0A384JGC8	*Bcmet6*	Bcmet6 OS=*Botryotinia fuckeliana* (strain B05.10) OX=332648 GN=Bcmet6 PE=3 SV=1	86.237	7	8.3	54049000	25.6878	1228400	0.214
A0A384JZC3	*BCIN_12g04380*	Uncharacterized protein OS=*Botryotinia fuckeliana* (strain B05.10) OX=332648 GN=BCIN_12g04380	118.5	11	9.4	74367000	26.1482	1219100	0.213

		PE=4 SV=1							
A0A384JIV2	*Bcerg10*	Bcerg10 OS=*Botryotinia fuckeliana* (strain B05.10) OX=332648 GN=Bcerg10 PE=3 SV=1	41.631	3	7.8	23789000	24.5038	1189500	0.208
A0A384J4N4	*BCIN_01g02580*	14_3_3 domain-containing protein OS=*Botryotinia fuckeliana* (strain B05.10) OX=332648 GN=BCIN_01g02580 PE=3 SV=1	29.623	3	10.6	22284000	24.4095	1172900	0.205
A0A384K179	*BCIN_13g03600*	KOW domain-containing protein OS=*Botryotinia fuckeliana* (strain B05.10) OX=332648 GN=BCIN_13g03600 PE=3 SV=1	15.533	3	13.2	9204300	23.1339	1150500	0.201
A0A384JBP8	*BCIN_03g03150*	Uncharacterized protein OS=*Botryotinia fuckeliana* (strain B05.10) OX=332648 GN=BCIN_03g03150 PE=4 SV=1	34.108	1	3.1	24744000	24.5606	1124700	0.196
A0A384JPW4	*BCIN_08g02290*	Uncharacterized protein OS=*Botryotinia fuckeliana* (strain B05.10) OX=332648 GN=BCIN_08g02290 PE=3 SV=1	30.935	2	6.8	16703000	23.9936	1113600	0.194
A0A384JKI4	*BCIN_06g05400*	Uncharacterized protein OS=*Botryotinia fuckeliana* (strain B05.10) OX=332648 GN=BCIN_06g05400 PE=3 SV=1	45.758	3	6.9	28802000	24.7797	1107800	0.193
A0A384K1G6	*BCIN_13g04730*	Uncharacterized protein OS=*Botryotinia fuckeliana* (strain B05.10) OX=332648 GN=BCIN_13g04730 PE=4 SV=1	11.769	1	10.2	5355700	22.3526	1071100	0.187
A0A384J8V3	*Bcggc1*	Bcggc1 OS=*Botryotinia fuckeliana* (strain B05.10) OX=332648 GN=Bcggc1 PE=3 SV=1	32.945	3	9.2	22026000	24.3927	1048800	0.183
A0A384JAP1	*BCIN_02g07930*	Uncharacterized protein OS=*Botryotinia fuckeliana* (strain B05.10) OX=332648 GN=BCIN_02g07930 PE=3 SV=1	30.864	1	3.4	16767000	23.9991	1047900	0.183
A0A384JQ57	*Bcpme1*	Pectinesterase OS=*Botryotinia fuckeliana* (strain B05.10) OX=332648 GN=Bcpme1 PE=4 SV=1	37.236	1	3.5	9281900	23.146	1031300	0.18
A0A384K7A2	*BCIN_16g04050*	Glutamate dehydrogenase OS=*Botryotinia fuckeliana* (strain B05.10) OX=332648 GN=BCIN_16g04050 PE=3 SV=1	49.045	2	4.4	29865000	24.832	1029800	0.18
A0A384JA13	*BCIN_02g06790*	Histone H2B OS=*Botryotinia fuckeliana* (strain B05.10) OX=332648 GN=BCIN_02g06790 PE=3 SV=1	14.941	2	12.9	13023000	23.6346	1001800	0.175
A0A384J5	*BCIN_0*	Uncharacterized	44.874	4	13.3	23468000	24.4842	977820	0.171

P0	*1g05590*	protein OS=*Botryotinia fuckeliana* (strain B05.10) OX=332648 GN=BCIN_01g05590 PE=3 SV=1							
A0A384J689	*Bcpre4*	Proteasome subunit beta OS=*Botryotinia fuckeliana* (strain B05.10) OX=332648 GN=Bcpre4 PE=3 SV=1	28.717	1	4.2	11004000	23.3915	916960	0.16
A0A384JNQ0	*Bcgdi1*	Rab GDP dissociation inhibitor OS=*Botryotinia fuckeliana* (strain B05.10) OX=332648 GN=Bcgdi1 PE=3 SV=1	51.913	3	6.6	25426000	24.5998	908060	0.159
R9U1R1	*BC1G_20005*	Cytochrome c oxidase subunit 2 OS=*Botryotinia fuckeliana* (strain B05.10) OX=332648 GN=BC1G_20005 PE=3 SV=1	28.885	1	2.8	7738500	22.8836	859840	0.15
A0A384K6F2	*BcactA*	BcactA OS=*Botryotinia fuckeliana* (strain B05.10) OX=332648 GN=BcactA PE=3 SV=1	41.639	4	7.7	19386000	24.2085	842860	0.147
A0A384JAJ7	*BCIN_03g00010*	Uncharacterized protein OS=*Botryotinia fuckeliana* (strain B05.10) OX=332648 GN=BCIN_03g00010 PE=4 SV=1	55.225	4	8	26774000	24.6743	836690	0.146
A0A384JTW8	*BCIN_10g00300*	HATPase_c domain-containing protein OS=*Botryotinia fuckeliana* (strain B05.10) OX=332648 GN=BCIN_10g00300 PE=3 SV=1	79.536	6	8.3	34962000	25.0593	832420	0.145
A0A384K2W1	*BCIN_14g01740*	PABS domain-containing protein OS=*Botryotinia fuckeliana* (strain B05.10) OX=332648 GN=BCIN_14g01740 PE=3 SV=1	33.126	4	13.1	13250000	23.6595	828100	0.145
A0A384JRG5	*BCIN_09g00600*	Uncharacterized protein OS=*Botryotinia fuckeliana* (strain B05.10) OX=332648 GN=BCIN_09g00600 PE=4 SV=1	32.264	2	5.2	12946000	23.626	809100	0.141
A0A384K1P6	*BCIN_13g05810*	Aldedh domain-containing protein OS=*Botryotinia fuckeliana* (strain B05.10) OX=332648 GN=BCIN_13g05810 PE=3 SV=1	53.6	4	7.5	26288000	24.6479	773180	0.135
A0A384J7A0	*Bcccp1*	Peroxidase OS=*Botryotinia fuckeliana* (strain B05.10) OX=332648 GN=Bcccp1 PE=3 SV=1	40.749	4	10.8	16836000	24.005	765270	0.134
A0A384JRQ2	*BCIN_09g01400*	Uncharacterized protein OS=*Botryotinia fuckeliana* (strain B05.10) OX=332648 GN=BCIN_09g01400 PE=4 SV=1	40.176	1	2.2	18278000	24.1236	761600	0.133
A0A384J6S6	*Bclgd1*	Bclgd1 OS=*Botryotinia fuckeliana* (strain B05.10) OX=332648 GN=Bclgd1 PE=4 SV=1	46.906	2	4.7	18180000	24.1158	727190	0.127

A0A384JLP4	*BCIN_07g00520*	60S ribosomal protein L27 OS=*Botryotinia fuckeliana* (strain B05.10) OX=332648 GN=BCIN_07g00520 PE=3 SV=1	15.611	2	14.1	5812300	22.4707	726540	0.127
A0A384JC69	*BCIN_03g02900*	CN hydrolase domain-containing protein OS=*Botryotinia fuckeliana* (strain B05.10) OX=332648 GN=BCIN_03g02900 PE=4 SV=1	37.26	1	3.8	16594000	23.9842	721470	0.126
A0A384JDD5	*BCIN_03g06500*	Aldo_ket_red domain-containing protein OS=*Botryotinia fuckeliana* (strain B05.10) OX=332648 GN=BCIN_03g06500 PE=4 SV=1	39.572	2	6	16388000	23.9661	712520	0.124
A0A384JGS5	*BCIN_05g01600*	60S ribosomal protein L20 OS=*Botryotinia fuckeliana* (strain B05.10) OX=332648 GN=BCIN_05g01600 PE=3 SV=1	20.741	2	12.1	8033100	22.9375	669430	0.117
A0A384K1U9	*BCIN_13g04410*	Histone H3 OS=*Botryotinia fuckeliana* (strain B05.10) OX=332648 GN=BCIN_13g04410 PE=3 SV=1	15.319	1	5.1	3889100	21.891	648190	0.113
A0A384JZN2	*Bclat1*	Acetyltransferase component of pyruvate dehydrogenase complex OS=*Botryotinia fuckeliana* (strain B05.10) OX=332648 GN=Bclat1 PE=3 SV=1	48.358	5	10.6	15912000	23.9236	636470	0.111
A0A384JGR8	*BCIN_05g00570*	TPT domain-containing protein OS=*Botryotinia fuckeliana* (strain B05.10) OX=332648 GN=BCIN_05g00570 PE=4 SV=1	43.767	1	1.5	5989500	22.514	598950	0.105
A0A384JWV9	*BCIN_11g03280*	Uncharacterized protein OS=*Botryotinia fuckeliana* (strain B05.10) OX=332648 GN=BCIN_11g03280 PE=3 SV=1	14.522	2	13.4	4659800	22.1518	582480	0.102
A0A384JX00	*BCIN_11g03520*	S-adenosylmethionine synthase OS=*Botryotinia fuckeliana* (strain B05.10) OX=332648 GN=BCIN_11g03520 PE=3 SV=1	43.121	3	7.3	9948800	23.2461	552710	0.097
A0A384K3A6	*Bcrpl5*	Bcrpl5 OS=*Botryotinia fuckeliana* (strain B05.10) OX=332648 GN=Bcrpl5 PE=3 SV=1	34.022	2	5.1	8224700	22.9715	548310	0.096
A0A384J776	*BCIN_01g09950*	Pyruvate carboxylase OS=*Botryotinia fuckeliana* (strain B05.10) OX=332648 GN=BCIN_01g09950 PE=4 SV=1	131.08	9	7.4	37622000	25.1651	537450	0.094
A0A384JKP9	*BCIN_06g05910*	Uncharacterized protein OS=*Botryotinia fuckeliana* (strain B05.10) OX=332648 GN=BCIN_06g05910 PE=4 SV=1	26.398	1	4.1	6979400	22.7347	536870	0.094

A0A384JC13	*Bcado1*	Bcado1 OS=*Botryotinia fuckeliana* (strain B05.10) OX=332648 GN=Bcado1 PE=4 SV=1	37.68	3	8.3	8889200	23.0836	522890	0.091
A0A384K1L2	*BCIN_13g05580*	PKS_ER domain-containing protein OS=*Botryotinia fuckeliana* (strain B05.10) OX=332648 GN=BCIN_13g05580 PE=3 SV=1	37.411	2	4.8	11275000	23.4266	512490	0.089
A0A384JSA7	*Bcfum1*	Bcfum1 OS=*Botryotinia fuckeliana* (strain B05.10) OX=332648 GN=Bcfum1 PE=3 SV=1	58.429	2	2.8	17729000	24.0796	506540	0.088
A0A384J6Z0	*Bcpic7*	Pyruvate kinase OS=*Botryotinia fuckeliana* (strain B05.10) OX=332648 GN=Bcpic7 PE=3 SV=1	57.905	5	8.9	18209000	24.1181	505810	0.088
A0A384JZJ1	*BCIN_12g05550*	Aminotran_1_2 domain-containing protein OS=*Botryotinia fuckeliana* (strain B05.10) OX=332648 GN=BCIN_12g05550 PE=4 SV=1	46.982	2	4.7	13565000	23.6934	502410	0.088
A0A384JI44	*Bcmtc1*	Bcmtc1 OS=*Botryotinia fuckeliana* (strain B05.10) OX=332648 GN=Bcmtc1 PE=4 SV=1	56.76	2	3.8	12768000	23.606	472910	0.083
A0A384J888	*Bcqcr2*	Bcqcr2 OS=*Botryotinia fuckeliana* (strain B05.10) OX=332648 GN=Bcqcr2 PE=4 SV=1	48.148	2	4.3	12560000	23.5823	465190	0.081
A0A384JD09	*BCIN_03g05330*	SOR_SNZ domain-containing protein OS=*Botryotinia fuckeliana* (strain B05.10) OX=332648 GN=BCIN_03g05330 PE=3 SV=1	33.189	3	8.7	7317400	22.8029	457340	0.08
A0A384J909	*Bcyhm2*	Bcyhm2 OS=*Botryotinia fuckeliana* (strain B05.10) OX=332648 GN=Bcyhm2 PE=3 SV=1	33.74	2	5.7	8657100	23.0455	455640	0.08
A0A384JLT4	*BCIN_06g07350*	Aldolase_II domain-containing protein OS=*Botryotinia fuckeliana* (strain B05.10) OX=332648 GN=BCIN_06g07350 PE=4 SV=1	33.132	3	9.8	8176700	22.9631	454260	0.079
A0A384K4A5	*BCIN_15g02120*	Glyceraldehyde-3-phosphate dehydrogenase OS=*Botryotinia fuckeliana* (strain B05.10) OX=332648 GN=BCIN_15g02120 PE=3 SV=1	36.599	5	14.8	7712200	22.8787	453660	0.079
A0A384K5G0	*Bcpgi1*	Glucose-6-phosphate isomerase OS=*Botryotinia fuckeliana* (strain B05.10) OX=332648 GN=Bcpgi1 PE=3 SV=1	60.949	4	8	13601000	23.6972	453370	0.079
A0A384JEU9	*Bcprx1*	Bcprx1 OS=*Botryotinia fuckeliana* (strain B05.10) OX=332648 GN=Bcprx1	36.216	2	7.2	10220000	23.2849	444330	0.078

		PE=4 SV=1							
A0A384JEM7	*BCIN_04g01310*	Uncharacterized protein OS=*Botryotinia fuckeliana* (strain B05.10) OX=332648 GN=BCIN_04g01310 PE=4 SV=1	41.133	1	3.7	3057300	21.5438	436750	0.076
A0A384JBF3	*Bccat5*	Catalase OS=*Botryotinia fuckeliana* (strain B05.10) OX=332648 GN=Bccat5 PE=3 SV=1	57.482	3	4.7	14653000	23.8047	430970	0.075
A0A384K3C6	*BCIN_14g04790*	Ribosomal protein L15 OS=*Botryotinia fuckeliana* (strain B05.10) OX=332648 GN=BCIN_14g04790 PE=3 SV=1	23.944	2	11.8	5159100	22.2987	429920	0.075
A0A384JI26	*BCIN_05g06130*	Uncharacterized protein OS=*Botryotinia fuckeliana* (strain B05.10) OX=332648 GN=BCIN_05g06130 PE=4 SV=1	14.04	2	12.8	4273500	22.027	427350	0.075
A0A384JV63	*BCIN_10g04650*	DUF89 domain-containing protein OS=*Botryotinia fuckeliana* (strain B05.10) OX=332648 GN=BCIN_10g04650 PE=4 SV=1	53.056	1	1.5	13933000	23.732	422210	0.074
A0A384K0P5	*CND6*	CND6 OS=*Botryotinia fuckeliana* (strain B05.10) OX=332648 GN=CND6 PE=4 SV=1	71.444	3	3.7	17127000	24.0298	417740	0.073
A0A384JVY6	*BCIN_10g04300*	Uncharacterized protein OS=*Botryotinia fuckeliana* (strain B05.10) OX=332648 GN=BCIN_10g04300 PE=3 SV=1	17.89	3	17.3	5418800	22.3695	416830	0.073
A0A384JHT7	*BCIN_05g04910*	PH_6 domain-containing protein OS=*Botryotinia fuckeliana* (strain B05.10) OX=332648 GN=BCIN_05g04910 PE=4 SV=1	61.366	2	3.7	9559100	23.1884	415620	0.073
A0A384JZI0	*BCIN_12g04690*	Uncharacterized protein OS=*Botryotinia fuckeliana* (strain B05.10) OX=332648 GN=BCIN_12g04690 PE=3 SV=1	48.101	3	6.3	9881200	23.2363	395250	0.069
A0A384JJ96	*BCIN_06g00400*	Aldo_ket_red domain-containing protein OS=*Botryotinia fuckeliana* (strain B05.10) OX=332648 GN=BCIN_06g00400 PE=4 SV=1	39.964	2	4.7	6673800	22.6701	392570	0.069
A0A384JWQ9	*Bcfba1*	Fructose-bisphosphate aldolase OS=*Botryotinia fuckeliana* (strain B05.10) OX=332648 GN=Bcfba1 PE=3 SV=1	39.241	1	2.2	7018600	22.7428	389920	0.068
A0A384JFN5	*BCIN_04g05860*	Uncharacterized protein OS=*Botryotinia fuckeliana* (strain B05.10) OX=332648 GN=BCIN_04g05860 PE=4 SV=1	26.13	1	3	6940200	22.7265	385570	0.067
A0A384JC	*Bcfur1*	Bcfur1	27.279	2	8.2	5188300	22.3068	370600	0.065

L4		OS=*Botryotinia fuckeliana* (strain B05.10) OX=332648 GN=Bcfur1 PE=4 SV=1							
A0A384JQ22	*BCIN_08g03530*	Uncharacterized protein OS=*Botryotinia fuckeliana* (strain B05.10) OX=332648 GN=BCIN_08g03530 PE=4 SV=1	13.097	1	7.9	3241000	21.628	360110	0.063
A0A384JV25	*BCIN_10g04610*	40S ribosomal protein S7 OS=*Botryotinia fuckeliana* (strain B05.10) OX=332648 GN=BCIN_10g04610 PE=3 SV=1	22.526	1	4	3906300	21.8974	355120	0.062
A0A384JG48	*Bccdc10*	Bccdc10 OS=*Botryotinia fuckeliana* (strain B05.10) OX=332648 GN=Bccdc10 PE=3 SV=1	39.032	1	2.6	9426400	23.1683	349130	0.061
A0A384JJU9	*BCIN_06g02380*	RmlD_sub_bind domain-containing protein OS=*Botryotinia fuckeliana* (strain B05.10) OX=332648 GN=BCIN_06g02380 PE=4 SV=1	32.992	3	8.9	5844000	22.4785	343760	0.06
A0A384JNL7	*Bchsp60*	Bchsp60 OS=*Botryotinia fuckeliana* (strain B05.10) OX=332648 GN=Bchsp60 PE=3 SV=1	61.162	5	8.8	12831000	23.6131	329000	0.057
A0A384J9F2	*Bcrpt6*	Bcrpt6 OS=*Botryotinia fuckeliana* (strain B05.10) OX=332648 GN=Bcrpt6 PE=3 SV=1	43.328	1	3.3	8944900	23.0926	319460	0.056
A0A384JD22	*BCIN_03g06990*	Uncharacterized protein OS=*Botryotinia fuckeliana* (strain B05.10) OX=332648 GN=BCIN_03g06990 PE=3 SV=1	15.9	2	13.3	3488200	21.7341	317110	0.055
A0A384J852	*BCIN_02g00470*	Uncharacterized protein OS=*Botryotinia fuckeliana* (strain B05.10) OX=332648 GN=BCIN_02g00470 PE=4 SV=1	31.545	3	8.5	4912500	22.228	307030	0.054
A0A384JEX2	*Bcpio13*	Aspartate aminotransferase OS=*Botryotinia fuckeliana* (strain B05.10) OX=332648 GN=Bcpio13 PE=4 SV=1	46.088	2	4	8849800	23.0772	305170	0.053
A0A384JK83	*BCIN_06g04150*	Uncharacterized protein OS=*Botryotinia fuckeliana* (strain B05.10) OX=332648 GN=BCIN_06g04150 PE=4 SV=1	27.598	1	5	4268100	22.0252	304860	0.053
A0A384JX78	*BCIN_11g04630*	Uncharacterized protein OS=*Botryotinia fuckeliana* (strain B05.10) OX=332648 GN=BCIN_11g04630 PE=3 SV=1	42.944	2	4.9	7175000	22.7745	298960	0.052
A0A384JUY8	*BCIN_10g04290*	Uncharacterized protein OS=*Botryotinia fuckeliana* (strain B05.10) OX=332648 GN=BCIN_10g04290 PE=3 SV=1	20.854	1	5.3	4091100	21.9641	292220	0.051

A0A384JCP8	*BCIN_03g05490*	Lactamase_B domain-containing protein OS=*Botryotinia fuckeliana* (strain B05.10) OX=332648 GN=BCIN_03g05490 PE=3 SV=1	35.946	3	7	6092900	22.5387	290140	0.051
A0A384K529	*Bcpom33*	Bcpom33 OS=*Botryotinia fuckeliana* (strain B05.10) OX=332648 GN=Bcpom33 PE=4 SV=1	31.078	2	8.6	4636900	22.1447	289800	0.051
A0A384JA92	*BCIN_02g06950*	Uncharacterized protein OS=*Botryotinia fuckeliana* (strain B05.10) OX=332648 GN=BCIN_02g06950 PE=3 SV=1	33.146	1	3	4595300	22.1317	287210	0.05
A0A384JM47	*BCIN_07g02370*	DJ-1_PfpI domain-containing protein OS=*Botryotinia fuckeliana* (strain B05.10) OX=332648 GN=BCIN_07g02370 PE=4 SV=1	31.615	1	3.9	4290000	22.0325	286000	0.05
A0A384JU56	*Bcgst1*	Bcgst1 OS=*Botryotinia fuckeliana* (strain B05.10) OX=332648 GN=Bcgst1 PE=3 SV=1	29.061	1	3.1	3988300	21.9273	284880	0.05
A0A384JVI2	*Bcrpg1*	Eukaryotic translation initiation factor 3 subunit A OS=*Botryotinia fuckeliana* (strain B05.10) OX=332648 GN=Bcrpg1 PE=3 SV=1	119.75	3	3.5	18485000	24.1399	280080	0.049
A0A384K2N5	*Bcsfa1*	S-(hydroxymethyl)glutathione dehydrogenase OS=*Botryotinia fuckeliana* (strain B05.10) OX=332648 GN=Bcsfa1 PE=3 SV=1	40.595	1	2.4	5708200	22.4446	271820	0.047
A0A384JGQ2	*Bcglr2*	Glutathione reductase OS=*Botryotinia fuckeliana* (strain B05.10) OX=332648 GN=Bcglr2 PE=3 SV=1	59.639	3	4.7	7269200	22.7934	269230	0.047
A0A384JZ76	*Bcdao1*	Bcdao1 OS=*Botryotinia fuckeliana* (strain B05.10) OX=332648 GN=Bcdao1 PE=4 SV=1	38.644	2	3.9	4474300	22.0932	263190	0.046
A0A384JBJ9	*BCIN_03g02740*	Arginine biosynthesis bifunctional protein ArgJ, mitochondrial OS=*Botryotinia fuckeliana* (strain B05.10) OX=332648 GN=BCIN_03g02740 PE=3 SV=1	50.324	3	7.2	7626300	22.8626	262980	0.046
A0A384JBH8	*BCIN_03g02320*	Eukaryotic translation initiation factor 3 subunit L OS=*Botryotinia fuckeliana* (strain B05.10) OX=332648 GN=BCIN_03g02320 PE=3 SV=1	54.853	2	3.4	6270700	22.5802	261280	0.046
A0A384K7Y8	*Bctom40*	Bctom40 OS=*Botryotinia fuckeliana* (strain B05.10) OX=332648 GN=Bctom40 PE=4 SV=1	38.441	1	2.5	3832700	21.8699	255510	0.045

A0A384J885	*Bcrpt1*	Bcrpt1 OS=*Botryotinia fuckeliana* (strain B05.10) OX=332648 GN=Bcrpt1 PE=3 SV=1	49.026	2	4.6	8519000	23.0223	250560	0.044
A0A384K0L0	*BCIN_13g01350*	Complex1_30kDa domain-containing protein OS=*Botryotinia fuckeliana* (strain B05.10) OX=332648 GN=BCIN_13g01350 PE=3 SV=1	33.253	1	3.8	4420100	22.0756	245560	0.043
A0A384JUZ5	*Bccp4*	Carboxypeptidase OS=*Botryotinia fuckeliana* (strain B05.10) OX=332648 GN=Bccp4 PE=3 SV=1	60.851	1	1.6	6331900	22.5942	243530	0.043
A0A384J5I1	*BCIN_01g06470*	Uncharacterized protein OS=*Botryotinia fuckeliana* (strain B05.10) OX=332648 GN=BCIN_01g06470 PE=4 SV=1	77.616	4	3.6	10200000	23.2821	242860	0.042
A0A384J3X8	*Bcfas2*	Bcfas2 OS=*Botryotinia fuckeliana* (strain B05.10) OX=332648 GN=Bcfas2 PE=3 SV=1	204.22	10	4.5	27393000	24.7073	242410	0.042
A0A384J5H7	*Bcarc40*	Actin-related protein 2/3 complex subunit OS=*Botryotinia fuckeliana* (strain B05.10) OX=332648 GN=Bcarc40 PE=3 SV=1	39.351	2	5.8	4830800	22.2038	241540	0.042
A0A384K1R6	*Bcarp2*	Actin-related protein 2 OS=*Botryotinia fuckeliana* (strain B05.10) OX=332648 GN=Bcarp2 PE=3 SV=1	44.036	1	2.1	5268300	22.3289	239470	0.042
A0A384JEN9	*BCIN_04g01400*	Uncharacterized protein OS=*Botryotinia fuckeliana* (strain B05.10) OX=332648 GN=BCIN_04g01400 PE=4 SV=1	20.215	1	4.8	2630400	21.3269	239130	0.042
A0A384JKW4	*Bcrps13*	Bcrps13 OS=*Botryotinia fuckeliana* (strain B05.10) OX=332648 GN=Bcrps13 PE=3 SV=1	16.814	2	15.2	2622900	21.3227	238450	0.042
A0A384J7N8	*BCIN_01g10430*	AB hydrolase-1 domain-containing protein OS=*Botryotinia fuckeliana* (strain B05.10) OX=332648 GN=BCIN_01g10430 PE=4 SV=1	40.546	2	4.8	4542300	22.115	227110	0.04
A0A384JSF0	*BCIN_09g03550*	KOW domain-containing protein OS=*Botryotinia fuckeliana* (strain B05.10) OX=332648 GN=BCIN_09g03550 PE=4 SV=1	15.936	1	5.5	2385800	21.186	216890	0.038
A0A384JH24	*BCIN_05g00940*	Uncharacterized protein OS=*Botryotinia fuckeliana* (strain B05.10) OX=332648 GN=BCIN_05g00940 PE=4 SV=1	59.3	1	1.5	7792400	22.8936	216460	0.038
A0A384K0D3	*Bckar2*	Bckar2 OS=*Botryotinia fuckeliana* (strain B05.10) OX=332648 GN=Bckar2 PE=3 SV=1	73.12	2	3.6	8361000	22.9952	214390	0.037

A0A384JKP7	*BCIN_06g05920*	Uncharacterized protein OS=*Botryotinia fuckeliana* (strain B05.10) OX=332648 GN=BCIN_06g05920 PE=4 SV=1	50.795	2	4.7	4444400	22.0836	211640	0.037
A0A384JWP0	*BCIN_11g03320*	HMG box domain-containing protein OS=*Botryotinia fuckeliana* (strain B05.10) OX=332648 GN=BCIN_11g03320 PE=4 SV=1	37.408	2	5.7	4646600	22.1477	211210	0.037
A0A384K036	*Bcarx1*	Bcarx1 OS=*Botryotinia fuckeliana* (strain B05.10) OX=332648 GN=Bcarx1 PE=4 SV=1	43.869	2	5.7	5218400	22.3152	208740	0.036
A0A384JYI5	*Bcidi1*	Bcidi1 OS=*Botryotinia fuckeliana* (strain B05.10) OX=332648 GN=Bcidi1 PE=4 SV=1	29.258	1	3.1	3510600	21.7433	206510	0.036
A0A384K724	*Bccys4*	Cystathionine beta-synthase OS=*Botryotinia fuckeliana* (strain B05.10) OX=332648 GN=Bccys4 PE=3 SV=1	57.237	2	2.8	5916200	22.4962	204010	0.036
A0A384JDA3	*BCIN_03g06810*	MIF4G domain-containing protein OS=*Botryotinia fuckeliana* (strain B05.10) OX=332648 GN=BCIN_03g06810 PE=4 SV=1	150.82	2	1.3	16115000	23.9419	201440	0.035
A0A384JKX7	*Bcpdi1*	Protein disulfide-isomerase OS=*Botryotinia fuckeliana* (strain B05.10) OX=332648 GN=Bcpdi1 PE=3 SV=1	57.485	2	4	6814000	22.7001	200410	0.035
A0A384JA37	*BCIN_02g06400*	Dihydrolipoyl dehydrogenase OS=*Botryotinia fuckeliana* (strain B05.10) OX=332648 GN=BCIN_02g06400 PE=3 SV=1	54.421	4	7.5	6086100	22.5371	196320	0.034
A0A384JJL3	*Bcrpn5*	Bcrpn5 OS=*Botryotinia fuckeliana* (strain B05.10) OX=332648 GN=Bcrpn5 PE=4 SV=1	56.608	2	3	6660500	22.6672	195900	0.034
A0A384J4B3	*BCIN_01g01400*	AB hydrolase-1 domain-containing protein OS=*Botryotinia fuckeliana* (strain B05.10) OX=332648 GN=BCIN_01g01400 PE=4 SV=1	31.044	1	3.6	2483100	21.2437	191010	0.033
A0A384K5K4	*Bcsak1*	Mitogen-activated protein kinase OS=*Botryotinia fuckeliana* (strain B05.10) OX=332648 GN=Bcsak1 PE=3 SV=1	39.391	1	3.2	3818800	21.8647	190940	0.033
A0A384JEP9	*Bcrho1*	Bcrho1 OS=*Botryotinia fuckeliana* (strain B05.10) OX=332648 GN=Bcrho1 PE=4 SV=1	21.555	1	6.8	2220300	21.0823	185030	0.032
A0A384K4K0	*Bccdc12*	Bccdc12 OS=*Botryotinia fuckeliana* (strain B05.10) OX=332648	44.354	1	2.8	4214600	22.007	183240	0.032

		GN=Bccdc12 PE=3 SV=1							
A0A384J9W3	*Bckgd2*	Bckgd2 OS=*Botryotinia fuckeliana* (strain B05.10) OX=332648 GN=Bckgd2 PE=4 SV=1	47.429	1	1.6	4639400	22.1455	178440	0.031
A0A384J4Q0	*Bccct2*	Bccct2 OS=*Botryotinia fuckeliana* (strain B05.10) OX=332648 GN=Bccct2 PE=3 SV=1	56.693	2	3.2	6236500	22.5723	178180	0.031
A0A384J6Q3	*BCIN_01g09550*	Uridylate kinase OS=*Botryotinia fuckeliana* (strain B05.10) OX=332648 GN=BCIN_01g09550 PE=3 SV=1	33.149	1	3	3546100	21.7578	177300	0.031
A0A384K2Y2	*Bcgln1*	Glutamine synthetase OS=*Botryotinia fuckeliana* (strain B05.10) OX=332648 GN=Bcgln1 PE=3 SV=1	40.613	1	2.2	3338600	21.6708	175710	0.031
A0A384JY21	*Bcgua1*	Bcgua1 OS=*Botryotinia fuckeliana* (strain B05.10) OX=332648 GN=Bcgua1 PE=3 SV=1	59.863	3	4.6	5765100	22.4589	174700	0.031
A0A384JZV7	*BCIN_12g06020*	Plasma membrane ATPase OS=*Botryotinia fuckeliana* (strain B05.10) OX=332648 GN=BCIN_12g06020 PE=3 SV=1	107.6	2	1.8	9025300	23.1055	167130	0.029
A0A384J4U8	*BCIN_01g03020*	Aldedh domain-containing protein OS=*Botryotinia fuckeliana* (strain B05.10) OX=332648 GN=BCIN_01g03020 PE=4 SV=1	61.483	2	3.3	6322600	22.5921	166380	0.029
A0A384J4E4	*Bchom2*	Bchom2 OS=*Botryotinia fuckeliana* (strain B05.10) OX=332648 GN=Bchom2 PE=4 SV=1	39.476	1	2.2	2958200	21.4963	164340	0.029
A0A384JSI7	*Bcpdh1*	Bcpdh1 OS=*Botryotinia fuckeliana* (strain B05.10) OX=332648 GN=Bcpdh1 PE=4 SV=1	61.172	1	1.6	6045100	22.5273	163380	0.029
A0A384JBE1	*BCIN_03g01540*	Uncharacterized protein OS=*Botryotinia fuckeliana* (strain B05.10) OX=332648 GN=BCIN_03g01540 PE=4 SV=1	66.881	2	2.5	3429400	21.7095	163310	0.029
A0A384JB13	*BCIN_02g07690*	Uncharacterized protein OS=*Botryotinia fuckeliana* (strain B05.10) OX=332648 GN=BCIN_02g07690 PE=4 SV=1	35.17	1	3.1	3204100	21.6115	160210	0.028
A0A384K330	*BCIN_14g03970*	1,3-beta-glucanosyltransferase OS=*Botryotinia fuckeliana* (strain B05.10) OX=332648 GN=BCIN_14g03970 PE=3 SV=1	47.687	1	2.4	2686900	21.3575	158050	0.028
A0A384JXL0	*BCIN_11g05900*	60S ribosomal protein L6 OS=*Botryotinia fuckeliana* (strain B05.10) OX=332648 GN=BCIN_11g05900	22.137	1	4	2346700	21.1622	156440	0.027

		PE=3 SV=1							
A0A384JGF9	*BCIN_05g00090*	Uncharacterized protein OS=*Botryotinia fuckeliana* (strain B05.10) OX=332648 GN=BCIN_05g00090 PE=3 SV=1	71.554	3	4.5	6536000	22.64	152000	0.027
A0A384JQG8	*BCIN_08g02860*	AB hydrolase-1 domain-containing protein OS=*Botryotinia fuckeliana* (strain B05.10) OX=332648 GN=BCIN_08g02860 PE=3 SV=1	57.095	1	1.5	4350000	22.0526	150000	0.026
A0A384J9T2	*Bcrpn9*	Bcrpn9 OS=*Botryotinia fuckeliana* (strain B05.10) OX=332648 GN=Bcrpn9 PE=4 SV=1	43.163	1	2.4	3633600	21.793	145340	0.025
A0A384J4R3	*Bcngr1*	Bcngr1 OS=*Botryotinia fuckeliana* (strain B05.10) OX=332648 GN=Bcngr1 PE=4 SV=1	44.221	1	2.2	2205800	21.0729	137860	0.024
A0A384JPH0	*Bcgb1*	Bcgb1 OS=*Botryotinia fuckeliana* (strain B05.10) OX=332648 GN=Bcgb1 PE=4 SV=1	39.539	1	2.2	2065400	20.978	129090	0.023
A0A384J9B9	*BCIN_02g05260*	Coatomer subunit alpha OS=*Botryotinia fuckeliana* (strain B05.10) OX=332648 GN=BCIN_02g05260 PE=4 SV=1	136.5	2	1.6	9643200	23.2011	128580	0.022
A0A384JGP9	*BCIN_05g01750*	Uncharacterized protein OS=*Botryotinia fuckeliana* (strain B05.10) OX=332648 GN=BCIN_05g01750 PE=4 SV=1	102.26	3	2.8	6102000	22.5409	127130	0.022
A0A384J855	*BCIN_02g00480*	Uncharacterized protein OS=*Botryotinia fuckeliana* (strain B05.10) OX=332648 GN=BCIN_02g00480 PE=4 SV=1	33.071	1	3.4	2285000	21.1238	126940	0.022
A0A384JET9	*Bcilv5*	Ketol-acid reductoisomerase, mitochondrial OS=*Botryotinia fuckeliana* (strain B05.10) OX=332648 GN=Bcilv5 PE=3 SV=1	44.169	2	4.5	2906200	21.4707	126360	0.022
A0A384JFR0	*BCIN_04g04190*	Glucoamylase OS=*Botryotinia fuckeliana* (strain B05.10) OX=332648 GN=BCIN_04g04190 PE=3 SV=1	70.617	1	1.3	2502900	21.2552	125150	0.022
A0A384J8K9	*BCIN_02g01720*	Phospholipase OS=*Botryotinia fuckeliana* (strain B05.10) OX=332648 GN=BCIN_02g01720 PE=3 SV=1	97.916	2	2.2	6192500	22.5621	123850	0.022
A0A384JV88	*Bcuga1*	Bcuga1 OS=*Botryotinia fuckeliana* (strain B05.10) OX=332648 GN=Bcuga1 PE=3 SV=1	54.692	3	6.7	3129900	21.5777	120380	0.021
A0A384JZF5	*Bctcp1*	Bctcp1 OS=*Botryotinia fuckeliana* (strain B05.10)	61.631	1	1.8	5019100	22.259	116720	0.02

		OX=332648 GN=Bctcp1 PE=3 SV=1							
A0A384JEU3	*Bcarg4*	Bcarg4 OS=*Botryotinia fuckeliana* (strain B05.10) OX=332648 GN=Bcarg4 PE=3 SV=1	52.654	1	1.5	3344400	21.6733	115320	0.02
A0A384J5X2	*BCIN_01g05720*	Uncharacterized protein OS=*Botryotinia fuckeliana* (strain B05.10) OX=332648 GN=BCIN_01g05720 PE=4 SV=1	82.549	1	1	2267700	21.1128	113380	0.02
A0A384JWC1	*Bcuga2*	Bcuga2 OS=*Botryotinia fuckeliana* (strain B05.10) OX=332648 GN=Bcuga2 PE=4 SV=1	53.742	1	1.8	2935300	21.4851	112890	0.02
A0A384K381	*BCIN_14g01970*	Uncharacterized protein OS=*Botryotinia fuckeliana* (strain B05.10) OX=332648 GN=BCIN_14g01970 PE=4 SV=1	68.977	1	1.1	3939500	21.9096	112560	0.02
A0A384K542	*Bcglc3*	Bcglc3 OS=*Botryotinia fuckeliana* (strain B05.10) OX=332648 GN=Bcglc3 PE=4 SV=1	80.146	3	4.1	4702600	22.165	111970	0.02
A0A384J6Q7	*BCIN_01g07880*	RRM domain-containing protein OS=*Botryotinia fuckeliana* (strain B05.10) OX=332648 GN=BCIN_01g07880 PE=4 SV=1	27.791	1	4.3	2330700	21.1523	110990	0.019
A0A384JZT0	*BCIN_12g06180*	Cyanide hydratase OS=*Botryotinia fuckeliana* (strain B05.10) OX=332648 GN=BCIN_12g06180 PE=2 SV=1	41.445	2	6.2	2307700	21.138	109890	0.019
A0A384JPI1	*Bczuo1*	Bczuo1 OS=*Botryotinia fuckeliana* (strain B05.10) OX=332648 GN=Bczuo1 PE=4 SV=1	50.521	1	2.2	2618800	21.3205	109120	0.019
A0A384JA99	*BCIN_02g08330*	Uncharacterized protein OS=*Botryotinia fuckeliana* (strain B05.10) OX=332648 GN=BCIN_02g08330 PE=4 SV=1	103.78	1	1	4104300	21.9687	108010	0.019
A0A384K2E1	*BCIN_14g01480*	DSBA domain-containing protein OS=*Botryotinia fuckeliana* (strain B05.10) OX=332648 GN=BCIN_14g01480 PE=4 SV=1	26.049	2	7.8	2105500	21.0057	105280	0.018
A0A384JLR9	*Bcsec62*	Bcsec62 OS=*Botryotinia fuckeliana* (strain B05.10) OX=332648 GN=Bcsec62 PE=4 SV=1	56.46	1	2.5	2753000	21.3926	101960	0.018
A0A384J4X4	*BCIN_01g03270*	NmrA domain-containing protein OS=*Botryotinia fuckeliana* (strain B05.10) OX=332648 GN=BCIN_01g03270 PE=4 SV=1	32.015	1	4.9	1917600	20.8709	100930	0.018
A0A384JD07	*Bcnip1*	Eukaryotic translation initiation factor 3 subunit C OS=*Botryotinia*	97.367	2	2.3	5507600	22.393	100140	0.017

		fuckeliana (strain B05.10) OX=332648 GN=Bcnip1 PE=3 SV=1						
A0A384J8J7	*BCIN_02g03480*	Abhydrolase_3 domain-containing protein OS=*Botryotinia fuckeliana* (strain B05.10) OX=332648 GN=BCIN_02g03480 PE=4 SV=1	35.051	1	3.1	1388900	20.4055	99209	0.017
A0A384K102	*BCIN_13g01450*	DUF2433 domain-containing protein OS=*Botryotinia fuckeliana* (strain B05.10) OX=332648 GN=BCIN_13g01450 PE=4 SV=1	74.992	3	3.7	3963000	21.9182	99075	0.017
A0A384JU22	*BCIN_10g01070*	Uncharacterized protein OS=*Botryotinia fuckeliana* (strain B05.10) OX=332648 GN=BCIN_10g01070 PE=4 SV=1	25.759	1	3.6	1376600	20.3927	91771	0.016
A0A384K617	*Bccrn1*	Coronin OS=*Botryotinia fuckeliana* (strain B05.10) OX=332648 GN=Bccrn1 PE=3 SV=1	66.405	1	1.3	3506400	21.7416	89907	0.016
A0A384JEL4	*Bcmet17*	Bcmet17 OS=*Botryotinia fuckeliana* (strain B05.10) OX=332648 GN=Bcmet17 PE=3 SV=1	48.869	1	2.2	2332900	21.1537	89725	0.016
A0A384JV52	*Bcnop1*	Bcnop1 OS=*Botryotinia fuckeliana* (strain B05.10) OX=332648 GN=Bcnop1 PE=3 SV=1	33.635	1	4	1696200	20.6939	89274	0.016
A0A384JXJ0	*BCIN_11g05910*	Dipeptidyl peptidase 3 OS=*Botryotinia fuckeliana* (strain B05.10) OX=332648 GN=BCIN_11g05910 PE=3 SV=1	85.57	1	1.3	4205800	22.0039	87620	0.015
A0A384JUK4	*BCIN_10g02340*	S-formylglutathione hydrolase OS=*Botryotinia fuckeliana* (strain B05.10) OX=332648 GN=BCIN_10g02340 PE=3 SV=1	31.222	1	3.8	1400600	20.4176	87535	0.015
A0A384JBN5	*Bclhs1*	Bclhs1 OS=*Botryotinia fuckeliana* (strain B05.10) OX=332648 GN=Bclhs1 PE=4 SV=1	112.75	1	1	5658300	22.4319	87051	0.015
A0A384K1A1	*CND16*	CND16 OS=*Botryotinia fuckeliana* (strain B05.10) OX=332648 GN=CND16 PE=4 SV=1	52.76	2	4.3	2436800	21.2166	87028	0.015
A0A384JQM6	*BCIN_08g04610*	Uncharacterized protein OS=*Botryotinia fuckeliana* (strain B05.10) OX=332648 GN=BCIN_08g04610 PE=3 SV=1	27.699	1	3.1	1383500	20.3999	86472	0.015
A0A384J9Y3	*Bcaco1*	Aconitate hydratase, mitochondrial OS=*Botryotinia fuckeliana* (strain B05.10) OX=332648 GN=Bcaco1 PE=3 SV=1	84.975	1	0.9	3446400	21.7167	84059	0.015
A0A384K5B9	*Bcnsr1*	Bcnsr1 OS=*Botryotinia*	47.441	2	4.8	1982800	20.9191	82618	0.014

		fuckeliana (strain B05.10) OX=332648 GN=Bcnsr1 PE=4 SV=1							
A0A384JSK1	*Bcsub2*	Bcsub2 OS=*Botryotinia fuckeliana* (strain B05.10) OX=332648 GN=Bcsub2 PE=4 SV=1	49.626	1	1.6	1971600	20.9109	82152	0.014
A0A384JEA0	*BCIN_04g01450*	Uncharacterized protein OS=*Botryotinia fuckeliana* (strain B05.10) OX=332648 GN=BCIN_04g01450 PE=4 SV=1	44.269	1	2.3	2350800	21.1647	81062	0.014
A0A384JWL4	*Bcsec26*	Coatomer subunit beta OS=*Botryotinia fuckeliana* (strain B05.10) OX=332648 GN=Bcsec26 PE=4 SV=1	106.39	2	1.8	4030900	21.9427	80617	0.014
A0A384J7Q3	*Bczwf1*	Glucose-6-phosphate 1-dehydrogenase OS=*Botryotinia fuckeliana* (strain B05.10) OX=332648 GN=Bczwf1 PE=3 SV=1	58.669	2	4.3	3304300	21.6559	80594	0.014
A0A384J9E8	*BCIN_02g04270*	Chromo domain-containing protein OS=*Botryotinia fuckeliana* (strain B05.10) OX=332648 GN=BCIN_02g04270 PE=4 SV=1	148.24	1	0.8	5246200	22.3228	79487	0.014
A0A384JBE2	*Bccse1*	Bccse1 OS=*Botryotinia fuckeliana* (strain B05.10) OX=332648 GN=Bccse1 PE=4 SV=1	108.76	3	2.5	4261300	22.0229	77479	0.014
A0A384K728	*BCIN_16g03540*	Uncharacterized protein OS=*Botryotinia fuckeliana* (strain B05.10) OX=332648 GN=BCIN_16g03540 PE=3 SV=1	61.712	1	1.9	2197800	21.0676	75787	0.013
A0A384JK75	*BCIN_06g03350*	Uncharacterized protein OS=*Botryotinia fuckeliana* (strain B05.10) OX=332648 GN=BCIN_06g03350 PE=3 SV=1	24.234	1	4.7	957760	19.8693	73674	0.013
A0A384K6I9	*Bcpre5*	Proteasome subunit alpha type OS=*Botryotinia fuckeliana* (strain B05.10) OX=332648 GN=Bcpre5 PE=3 SV=1	28.809	3	10.9	1227400	20.2272	72203	0.013
A0A384K2U8	*Bcdbp5*	Bcdbp5 OS=*Botryotinia fuckeliana* (strain B05.10) OX=332648 GN=Bcdbp5 PE=4 SV=1	51.788	1	1.9	1924500	20.8761	71278	0.012
A0A384JWF9	*BCIN_11g02610*	Obg-like ATPase 1 OS=*Botryotinia fuckeliana* (strain B05.10) OX=332648 GN=BCIN_11g02610 PE=3 SV=1	43.96	1	2.5	1995300	20.9282	68805	0.012
A0A384JVY1	*Bcerg13*	3-hydroxy-3-methylglutaryl coenzyme A synthase OS=*Botryotinia fuckeliana* (strain B05.10) OX=332648 GN=Bcerg13 PE=3 SV=1	50.347	1	2.2	1684000	20.6835	67358	0.012
A0A384JD73	*Bccat2*	Bccat2 OS=*Botryotinia*	72.539	1	1.3	2720400	21.3754	66350	0.012

		fuckeliana (strain B05.10) OX=332648 GN=Bccat2 PE=3 SV=1							
A0A384JH70	*BCIN_05g03230*	Uncharacterized protein OS=*Botryotinia fuckeliana* (strain B05.10) OX=332648 GN=BCIN_05g03230 PE=3 SV=1	92.849	1	1.1	3181000	21.601	66271	0.012
A0A384JMV1	*Bccmk1*	Bccmk1 OS=*Botryotinia fuckeliana* (strain B05.10) OX=332648 GN=Bccmk1 PE=3 SV=1	45.801	1	2.4	1584000	20.5951	65999	0.012
A0A384JNT7	*BCIN_07g06960*	Uncharacterized protein OS=*Botryotinia fuckeliana* (strain B05.10) OX=332648 GN=BCIN_07g06960 PE=4 SV=1	253.58	4	1.7	9730400	23.2141	65746	0.011
A0A384JSR0	*BCIN_09g02790*	Acetyl-coenzyme A synthetase OS=*Botryotinia fuckeliana* (strain B05.10) OX=332648 GN=BCIN_09g02790 PE=3 SV=1	73.978	1	1.5	2365100	21.1735	65696	0.011
A0A384JMH8	*BCIN_07g03460*	Uncharacterized protein OS=*Botryotinia fuckeliana* (strain B05.10) OX=332648 GN=BCIN_07g03460 PE=4 SV=1	42.149	1	3.7	1177900	20.1678	65438	0.011
A0A384JR47	*Bcagc1*	Bcagc1 OS=*Botryotinia fuckeliana* (strain B05.10) OX=332648 GN=Bcagc1 PE=3 SV=1	78.389	2	2.4	3001400	21.5172	65248	0.011
A0A384JGI5	*Bckgd1*	Bckgd1 OS=*Botryotinia fuckeliana* (strain B05.10) OX=332648 GN=Bckgd1 PE=4 SV=1	118.45	2	2.1	3707500	21.822	62838	0.011
A0A384JG51	*BCIN_05g00180*	Uncharacterized protein OS=*Botryotinia fuckeliana* (strain B05.10) OX=332648 GN=BCIN_05g00180 PE=3 SV=1	66.856	1	1.3	1964500	20.9057	59530	0.01
A0A384J9Q1	*BCIN_02g06270*	Uncharacterized protein OS=*Botryotinia fuckeliana* (strain B05.10) OX=332648 GN=BCIN_02g06270 PE=3 SV=1	58.469	1	1.9	2060600	20.9746	58876	0.01
A0A384JG93	*BCIN_05g00580*	Uncharacterized protein OS=*Botryotinia fuckeliana* (strain B05.10) OX=332648 GN=BCIN_05g00580 PE=4 SV=1	57.317	1	2.9	1566700	20.5793	58028	0.01
A0A384JBL3	*BCIN_03g01130*	Aldedh domain-containing protein OS=*Botryotinia fuckeliana* (strain B05.10) OX=332648 GN=BCIN_03g01130 PE=3 SV=1	60.749	2	3.6	2253400	21.1037	57779	0.01
A0A384JA65	*INT6*	Eukaryotic translation initiation factor 3 subunit E OS=*Botryotinia fuckeliana* (strain B05.10) OX=332648 GN=INT6 PE=3 SV=1	51.796	1	2.2	1669900	20.6713	57582	0.01

A0A384JMY5	*Bcidh2*	Isocitrate dehydrogenase [NAD] subunit, mitochondrial OS=*Botryotinia fuckeliana* (strain B05.10) OX=332648 GN=Bcidh2 PE=3 SV=1	41.61	1	2.3	1362500	20.3778	56770	0.01
A0A384J3V4	*BCIN_01g00170*	AA_permease domain-containing protein OS=*Botryotinia fuckeliana* (strain B05.10) OX=332648 GN=BCIN_01g00170 PE=4 SV=1	67.1	1	2.1	1151400	20.135	54828	0.01
A0A384JTU3	*Bcbna6*	Nicotinate-nucleotide pyrophosphorylase [carboxylating] OS=*Botryotinia fuckeliana* (strain B05.10) OX=332648 GN=Bcbna6 PE=3 SV=1	31.646	1	2.7	910770	19.7967	53575	0.009
A0A384K4P1	*Bcaim24*	Altered inheritance of mitochondria protein 24, mitochondrial OS=*Botryotinia fuckeliana* (strain B05.10) OX=332648 GN=Bcaim24 PE=3 SV=1	41.249	2	4.8	1549300	20.5632	53423	0.009
A0A384JQ98	*BCIN_08g02390*	Uncharacterized protein OS=*Botryotinia fuckeliana* (strain B05.10) OX=332648 GN=BCIN_08g02390 PE=4 SV=1	60.221	2	3.7	1043400	19.9929	52169	0.009
A0A384JST8	*BCIN_09g04160*	Uncharacterized protein OS=*Botryotinia fuckeliana* (strain B05.10) OX=332648 GN=BCIN_09g04160 PE=4 SV=1	80.87	1	1.5	1802900	20.7819	51512	0.009
A0A384JJP0	*BCIN_06g01640*	Succinate-semialdehyde dehydrogenase OS=*Botryotinia fuckeliana* (strain B05.10) OX=332648 GN=BCIN_06g01640 PE=3 SV=1	56.593	1	1.5	1583100	20.5943	51069	0.009
A0A384JWU5	*BCIN_11g03020*	Uncharacterized protein OS=*Botryotinia fuckeliana* (strain B05.10) OX=332648 GN=BCIN_11g03020 PE=4 SV=1	56.77	1	2.3	1525100	20.5405	50837	0.009
A0A384JEJ7	*Bcoxp1*	Bcoxp1 OS=*Botryotinia fuckeliana* (strain B05.10) OX=332648 GN=Bcoxp1 PE=4 SV=1	149.48	3	2	4023500	21.94	49673	0.009
A0A384K3U2	*BCIN_15g00140*	Fumarate reductase OS=*Botryotinia fuckeliana* (strain B05.10) OX=332648 GN=BCIN_15g00140 PE=3 SV=1	53.333	2	3.8	1424500	20.442	45953	0.008
A0A384JAD8	*Bctps1*	Trehalose-6-phosphate synthase OS=*Botryotinia fuckeliana* (strain B05.10) OX=332648 GN=Bctps1 PE=3 SV=1	59.349	1	1.7	1131100	20.1093	45242	0.008
A0A384JQ39	*Bccdc48*	Bccdc48 OS=*Botryotinia fuckeliana* (strain B05.10)	90.032	1	1.2	2164300	21.0455	45090	0.008

		OX=332648 GN=Bccdc48 PE=3 SV=1							
A0A384K577	*Bcglt1*	Bcglt1 OS=*Botryotinia fuckeliana* (strain B05.10) OX=332648 GN=Bcglt1 PE=4 SV=1	236.28	1	0.5	5348500	22.3507	44571	0.008
A0A384K0F1	*Bcncr1*	Bcncr1 OS=*Botryotinia fuckeliana* (strain B05.10) OX=332648 GN=Bcncr1 PE=4 SV=1	140.65	1	0.8	2590400	21.3047	43173	0.008
A0A384JH41	*Bcsti1*	Bcsti1 OS=*Botryotinia fuckeliana* (strain B05.10) OX=332648 GN=Bcsti1 PE=4 SV=1	64.998	1	1.2	1855100	20.8231	42160	0.007
A0A384JR08	*BCIN_08g06300*	ATP-dependent 6-phosphofructokinase OS=*Botryotinia fuckeliana* (strain B05.10) OX=332648 GN=BCIN_08g06300 PE=3 SV=1	86.569	1	1.3	1724700	20.7179	42066	0.007
A0A384J5E9	*BCIN_01g05060*	Aminopeptidase OS=*Botryotinia fuckeliana* (strain B05.10) OX=332648 GN=BCIN_01g05060 PE=3 SV=1	99.229	1	1.5	2407300	21.199	41505	0.007
A0A384JZ93	*BCIN_12g04670*	AA_TRNA_LIGASE_II domain-containing protein OS=*Botryotinia fuckeliana* (strain B05.10) OX=332648 GN=BCIN_12g04670 PE=3 SV=1	66.123	1	1.9	1440700	20.4583	40020	0.007
A0A384JRS9	*BCIN_09g00980*	Isocitrate dehydrogenase [NADP] OS=*Botryotinia fuckeliana* (strain B05.10) OX=332648 GN=BCIN_09g00980 PE=3 SV=1	50.36	1	2.2	1219900	20.2183	39351	0.007
A0A384K6A0	*Bccct8*	Bccct8 OS=*Botryotinia fuckeliana* (strain B05.10) OX=332648 GN=Bccct8 PE=3 SV=1	59.744	1	1.8	1630700	20.6371	37924	0.007
A0A384K807	*BCIN_17g00040*	AB hydrolase-1 domain-containing protein OS=*Botryotinia fuckeliana* (strain B05.10) OX=332648 GN=BCIN_17g00040 PE=4 SV=1	34.828	2	5.8	699360	19.4157	34968	0.006
A0A384K2U7	*Bccrm1*	Bccrm1 OS=*Botryotinia fuckeliana* (strain B05.10) OX=332648 GN=Bccrm1 PE=4 SV=1	122.85	1	1	1913600	20.8679	34792	0.006
A0A384J6S5	*Bcchc1*	Clathrin heavy chain OS=*Botryotinia fuckeliana* (strain B05.10) OX=332648 GN=Bcchc1 PE=3 SV=1	189.4	1	0.7	3426900	21.7085	34615	0.006
A0A384JSX9	*Bcglk*	Phosphotransferase OS=*Botryotinia fuckeliana* (strain B05.10) OX=332648 GN=Bcglk PE=3 SV=1	60.598	2	4.1	1309900	20.321	34470	0.006
A0A384K4T4	*BCIN_15g03630*	Uncharacterized protein OS=*Botryotinia fuckeliana* (strain B05.10) OX=332648	27.667	1	5	618520	19.2385	34362	0.006

		GN=BCIN_15g03630 PE=4 SV=1							
A0A384J5D0	*Bcmet3*	Sulfate adenylyltransferase OS=*Botryotinia fuckeliana* (strain B05.10) OX=332648 GN=Bcmet3 PE=3 SV=1	64.035	2	3.7	1177300	20.1671	33638	0.006
A0A384JCR3	*BCIN_03g04790*	Aldo_ket_red domain-containing protein OS=*Botryotinia fuckeliana* (strain B05.10) OX=332648 GN=BCIN_03g04790 PE=4 SV=1	35.529	1	2.8	771010	19.5564	33522	0.006
A0A384K3G6	*PSD2*	Phosphatidylserine decarboxylase proenzyme 2 OS=*Botryotinia fuckeliana* (strain B05.10) OX=332648 GN=PSD2 PE=3 SV=1	124.67	2	1.5	1975900	20.9141	32392	0.006
A0A384JGD5	*Bcpan1*	Bcpan1 OS=*Botryotinia fuckeliana* (strain B05.10) OX=332648 GN=Bcpan1 PE=4 SV=1	155.9	1	0.6	2083200	20.9904	31564	0.006
A0A384K0M2	*Bcded81*	Bcded81 OS=*Botryotinia fuckeliana* (strain B05.10) OX=332648 GN=Bcded81 PE=4 SV=1	66.013	1	1.9	928590	19.8247	30953	0.005
A0A384JD53	*BCIN_03g04810*	Polyadenylate-binding protein OS=*Botryotinia fuckeliana* (strain B05.10) OX=332648 GN=BCIN_03g04810 PE=3 SV=1	85.897	1	1.6	1158600	20.144	30489	0.005
A0A384JL58	*BCIN_06g05380*	Kinesin-like protein OS=*Botryotinia fuckeliana* (strain B05.10) OX=332648 GN=BCIN_06g05380 PE=3 SV=1	102.78	1	1.2	1890300	20.8502	30005	0.005
A0A384JD96	*BCIN_03g07250*	TIP120 domain-containing protein OS=*Botryotinia fuckeliana* (strain B05.10) OX=332648 GN=BCIN_03g07250 PE=4 SV=1	146.13	1	0.8	2324100	21.1482	29052	0.005
A0A384J7Y1	*Bcgdb1*	Bcgdb1 OS=*Botryotinia fuckeliana* (strain B05.10) OX=332648 GN=Bcgdb1 PE=4 SV=1	174.37	1	0.8	2652000	21.3386	28516	0.005
A0A384JFH9	*BCIN_04g05060*	Pribosyltran_N domain-containing protein OS=*Botryotinia fuckeliana* (strain B05.10) OX=332648 GN=BCIN_04g05060 PE=4 SV=1	34.771	1	3.1	569320	19.1189	28466	0.005
A0A384JCH1	*Bcsec61*	Bcsec61 OS=*Botryotinia fuckeliana* (strain B05.10) OX=332648 GN=Bcsec61 PE=3 SV=1	52.121	1	2.1	484040	18.8848	26891	0.005
A0A384K1L5	*BCIN_13g05370*	NTP_transferase domain-containing protein OS=*Botryotinia fuckeliana* (strain B05.10) OX=332648 GN=BCIN_13g05370 PE=4 SV=1	48.751	1	2.3	775670	19.5651	26747	0.005

A0A384J9S5	*Bcths1*	Bcths1 OS=*Botryotinia fuckeliana* (strain B05.10) OX=332648 GN=Bcths1 PE=3 SV=1	89.282	1	1.4	1378500	20.3947	25528	0.004
A0A384JN27	*BCIN_07g04630*	Epimerase domain-containing protein OS=*Botryotinia fuckeliana* (strain B05.10) OX=332648 GN=BCIN_07g04630 PE=4 SV=1	42.43	1	2.9	688260	19.3926	25491	0.004
A0A384JMM8	*BCIN_07g03810*	Uncharacterized protein OS=*Botryotinia fuckeliana* (strain B05.10) OX=332648 GN=BCIN_07g03810 PE=3 SV=1	77.792	2	2.3	1189800	20.1823	24282	0.004
A0A384J4X7	*BCIN_01g03440*	Uncharacterized protein OS=*Botryotinia fuckeliana* (strain B05.10) OX=332648 GN=BCIN_01g03440 PE=3 SV=1	124.3	1	1.1	1569200	20.5816	23775	0.004
A0A384J667	*Bcpol30*	Proliferating cell nuclear antigen OS=*Botryotinia fuckeliana* (strain B05.10) OX=332648 GN=Bcpol30 PE=3 SV=1	28.38	1	3.1	372180	18.5056	23262	0.004
A0A384JA51	*BCIN_02g05890*	CRAL-TRIO domain-containing protein OS=*Botryotinia fuckeliana* (strain B05.10) OX=332648 GN=BCIN_02g05890 PE=4 SV=1	72.58	1	1.1	820810	19.6467	20520	0.004
A0A384JVE0	*BCIN_10g05060*	Uncharacterized protein OS=*Botryotinia fuckeliana* (strain B05.10) OX=332648 GN=BCIN_10g05060 PE=4 SV=1	199.45	1	0.5	1770700	20.7559	17192	0.003
A0A384JZP0	*Bckap114*	Bckap114 OS=*Botryotinia fuckeliana* (strain B05.10) OX=332648 GN=Bckap114 PE=4 SV=1	113.07	1	1.1	873050	19.7357	16789	0.003
A0A384JZB5	*BCIN_12g04860*	Uncharacterized protein OS=*Botryotinia fuckeliana* (strain B05.10) OX=332648 GN=BCIN_12g04860 PE=3 SV=1	246.57	1	0.4	2043700	20.9628	16220	0.003
A0A384K7Z3	*BCIN_17g00010*	Aminotran_1_2 domain-containing protein OS=*Botryotinia fuckeliana* (strain B05.10) OX=332648 GN=BCIN_17g00010 PE=4 SV=1	47.067	1	1.6	345440	18.3981	15019	0.003
A0A384JNF8	*BCIN_07g06220*	Uncharacterized protein OS=*Botryotinia fuckeliana* (strain B05.10) OX=332648 GN=BCIN_07g06220 PE=3 SV=1	73.476	2	2.5	609960	19.2184	14877	0.003
A0A384K561	*BCIN_15g04410*	Peptidase_M3 domain-containing protein OS=*Botryotinia fuckeliana* (strain B05.10) OX=332648 GN=BCIN_15g04410 PE=3 SV=1	80.015	1	1.3	617230	19.2354	14028	0.002

A0A384JKX2	*Bcrgd2*	Bcrgd2 OS=*Botryotinia fuckeliana* (strain B05.10) OX=332648 GN=Bcrgd2 PE=4 SV=1	99.655	1	1.7	685670	19.3872	11822	0.002
A0A384J7Z9	*Bcrot2*	Bcrot2 OS=*Botryotinia fuckeliana* (strain B05.10) OX=332648 GN=Bcrot2 PE=3 SV=1	109.02	1	0.8	604640	19.2057	11408	0.002
A0A384J3Y3	*BCIN_01g00450*	PKS_AT domain-containing protein OS=*Botryotinia fuckeliana* (strain B05.10) OX=332648 GN=BCIN_01g00450 PE=3 SV=1	233.47	2	1	1274800	20.2818	10364	0.002
A0A384JBU5	*BCIN_03g03130*	Pre-mRNA-splicing factor OS=*Botryotinia fuckeliana* (strain B05.10) OX=332648 GN=BCIN_03g03130 PE=3 SV=1	163.38	1	0.8	581670	19.1498	7181.1	0.001
A0A384JGL5	*BCIN_05g01550*	Aldo_ket_red domain-containing protein OS=*Botryotinia fuckeliana* (strain B05.10) OX=332648 GN=BCIN_05g01550 PE=4 SV=1	36.325	1	2.5	99222	16.5984	4510.1	0.001
A0A384J899	*Bcadk1*	Adenylate kinase OS=*Botryotinia fuckeliana* (strain B05.10) OX=332648 GN=Bcadk1 PE=3 SV=1	29.562	1	2.9	0	NA	0	0
A0A384JA39	*BCIN_02g07780*	Uncharacterized protein OS=*Botryotinia fuckeliana* (strain B05.10) OX=332648 GN=BCIN_02g07780 PE=4 SV=1	49.858	1	1.3	0	NA	0	0
A0A384JBR1	*Bclga1*	Bclga1 OS=*Botryotinia fuckeliana* (strain B05.10) OX=332648 GN=Bclga1 PE=3 SV=1	34.904	1	2.5	0	NA	0	0
A0A384JBW9	*Bchom3*	Aspartokinase OS=*Botryotinia fuckeliana* (strain B05.10) OX=332648 GN=Bchom3 PE=3 SV=1	57.818	1	2.1	0	NA	0	0
A0A384JD00	*BCIN_03g06460*	Uncharacterized protein OS=*Botryotinia fuckeliana* (strain B05.10) OX=332648 GN=BCIN_03g06460 PE=4 SV=1	38.915	1	2.8	0	NA	0	0
A0A384JFX6	*Bcvma2*	Vacuolar proton pump subunit B OS=*Botryotinia fuckeliana* (strain B05.10) OX=332648 GN=Bcvma2 PE=3 SV=1	57.599	1	1.9	0	NA	0	0
A0A384JFY2	*BCIN_04g06450*	Aldo_ket_red domain-containing protein OS=*Botryotinia fuckeliana* (strain B05.10) OX=332648 GN=BCIN_04g06450 PE=4 SV=1	38.127	1	2.1	0	NA	0	0
A0A384JG89	*BCIN_04g05780*	MICOS complex subunit MIC12 OS=*Botryotinia fuckeliana* (strain B05.10) OX=332648 GN=BCIN_04g05780	31.114	1	3.2	0	NA	0	0

		PE=3 SV=1							
A0A384JHX3	*Bcsec18*	Bcsec18 OS=*Botryotinia fuckeliana* (strain B05.10) OX=332648 GN=Bcsec18 PE=4 SV=1	90.157	1	1	0	NA	0	0
A0A384JKJ7	*BCIN_06g04640*	Uncharacterized protein OS=*Botryotinia fuckeliana* (strain B05.10) OX=332648 GN=BCIN_06g04640 PE=4 SV=1	217.81	1	0.4	0	NA	0	0
A0A384JLZ5	*BCIN_07g00400*	HpcH_HpaI domain-containing protein OS=*Botryotinia fuckeliana* (strain B05.10) OX=332648 GN=BCIN_07g00400 PE=4 SV=1	33.46	1	2.9	0	NA	0	0
A0A384JPY4	*Bcser2*	Bcser2 OS=*Botryotinia fuckeliana* (strain B05.10) OX=332648 GN=Bcser2 PE=3 SV=1	55.267	2	4.6	0	NA	0	0
A0A384K050	*BCIN_12g06710*	M20_dimer domain-containing protein OS=*Botryotinia fuckeliana* (strain B05.10) OX=332648 GN=BCIN_12g06710 PE=4 SV=1	44.184	1	2.7	0	NA	0	0
A0A384K1W5	*BCIN_14g00480*	Beta-glucosidase OS=*Botryotinia fuckeliana* (strain B05.10) OX=332648 GN=BCIN_14g00480 PE=3 SV=1	91.483	1	1.1	0	NA	0	0
A0A384K240	*Bcagx1*	Bcagx1 OS=*Botryotinia fuckeliana* (strain B05.10) OX=332648 GN=Bcagx1 PE=3 SV=1	41.672	1	2.1	0	NA	0	0
A0A384K2B8	*Bcaif1*	Bcaif1 OS=*Botryotinia fuckeliana* (strain B05.10) OX=332648 GN=Bcaif1 PE=4 SV=1	63.148	1	1.5	0	NA	0	0
A0A384K2K4	*BCIN_14g02290*	Uncharacterized protein OS=*Botryotinia fuckeliana* (strain B05.10) OX=332648 GN=BCIN_14g02290 PE=4 SV=1	144.44	1	0.8	0	NA	0	0
A0A384K4W2	*Bcmcd4*	Bcmcd4 OS=*Botryotinia fuckeliana* (strain B05.10) OX=332648 GN=Bcmcd4 PE=4 SV=1	111.41	1	0.9	0	NA	0	0
A0A384K7E5	*BCIN_16g04330*	Protein kinase domain-containing protein OS=*Botryotinia fuckeliana* (strain B05.10) OX=332648 GN=BCIN_16g04330 PE=3 SV=1	45.516	1	2.5	0	NA	0	0

Table S3. Primer sequence for Bcthi4 protein expression.

Protein	Forward primer	Reverse primer

Bcthi4 WT	ACAGAGAACAGATTGGTGGTGGATCCATGGCACCGCCGAGTGCCCTGTTTAC	CAGTGGTGGTGGTGGTGGTGCTCGAGATAGGCATTCTGTGCTTTACGTTC
Bcthi4 MT	ATTGAGGCTCACAGAGAACAGATTGGTGGTGGATCCatggcaccgccgagtgccctgtttaccgg	GATCTCAGTGGTGGTGGTGGTGGTGCTCGAGATAGGCATTCTGTGCTTTACGTTCATCAAACACACGCAGTGCTTCTTCT

Table S4. Primer sequence for RT-qPCR.

Gene name	Forward primer	Reverse primer
Bcthi4	gTCAAgCACgCTgCTTTATTC	gTCACCACTCCTgCAATTCT
GAPDH	TAAGGATTGGCGTGGAGGA	AGTTTGCCGTTAAGGACTGG

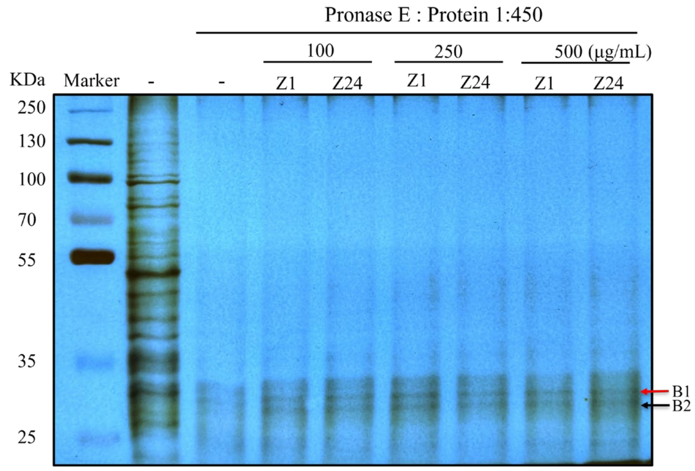

Fig. S1. ***B. cinerea*** **Pers. cell lysates were incubated with Z1 and Z24** ***in vitro*****, followed by protease digestion and silver staining.**

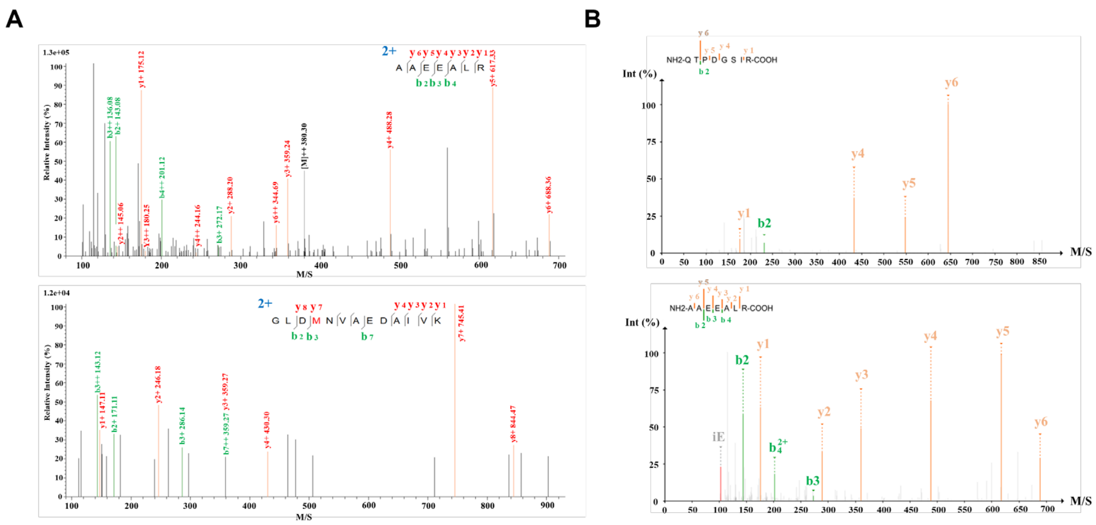

Fig. S2. Bcthi4 profile. (**A**) Bcthi4 peptide profile. (**B**) Secondary spectrum of Bcthi4 protein.

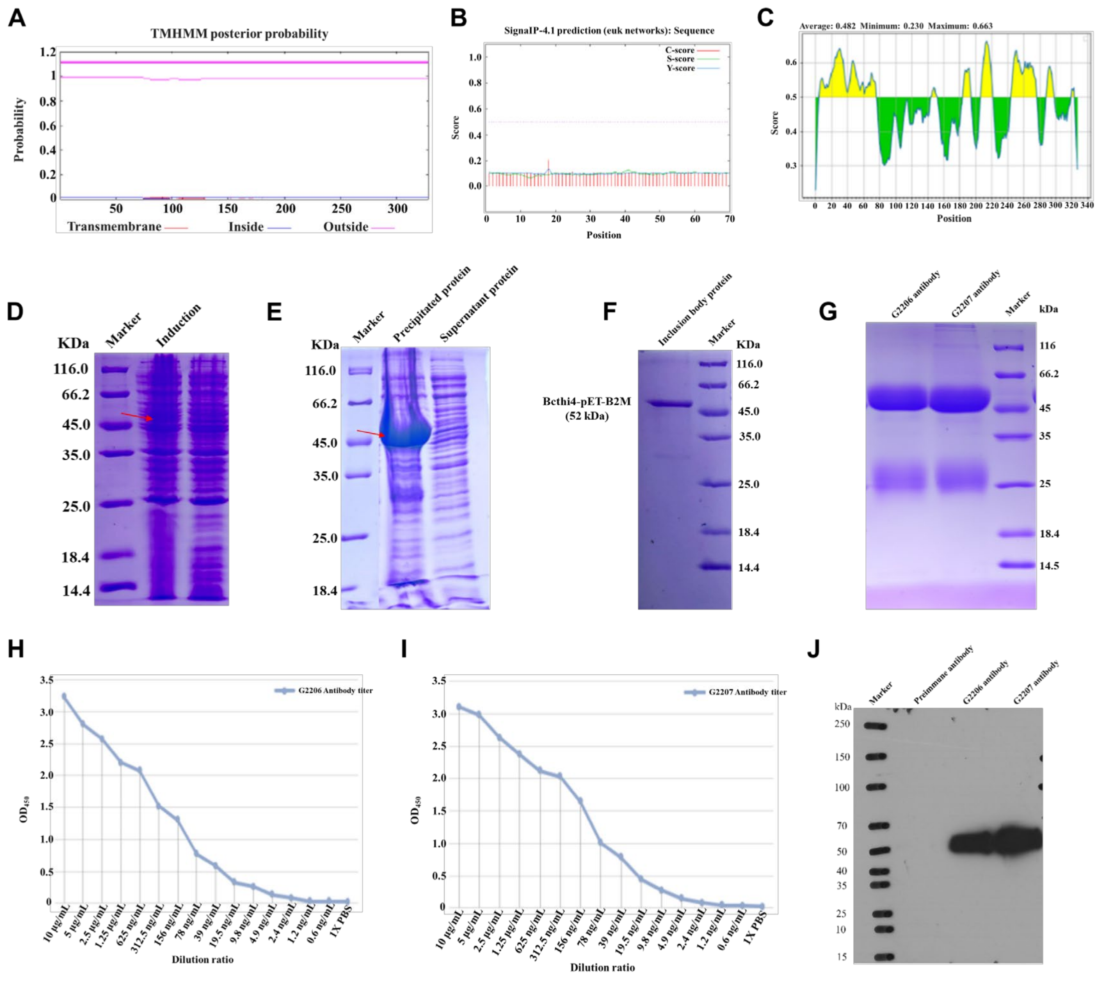

Fig. S3. Preparation of Bcthi4 antibody. (**A**) Bcthi4 transmembrane region prediction. (**B**) Bcthi4

signaling peptide prediction. (**C**) Antigenicity analysis of Bcthi4. (**D**) Small amount of antigen protein expression. (**E**) Antigen expression detection. (**F**) Antigen inclusion body purification detection. (**G**) SDS diagram of antibody purification. (**H**) Antibody titer detection for G2206. (**I**) Antibody titer detection for G2207. (**J**) Rabbit polyclonal antibody against *Bcthi4* gene specificity detection.

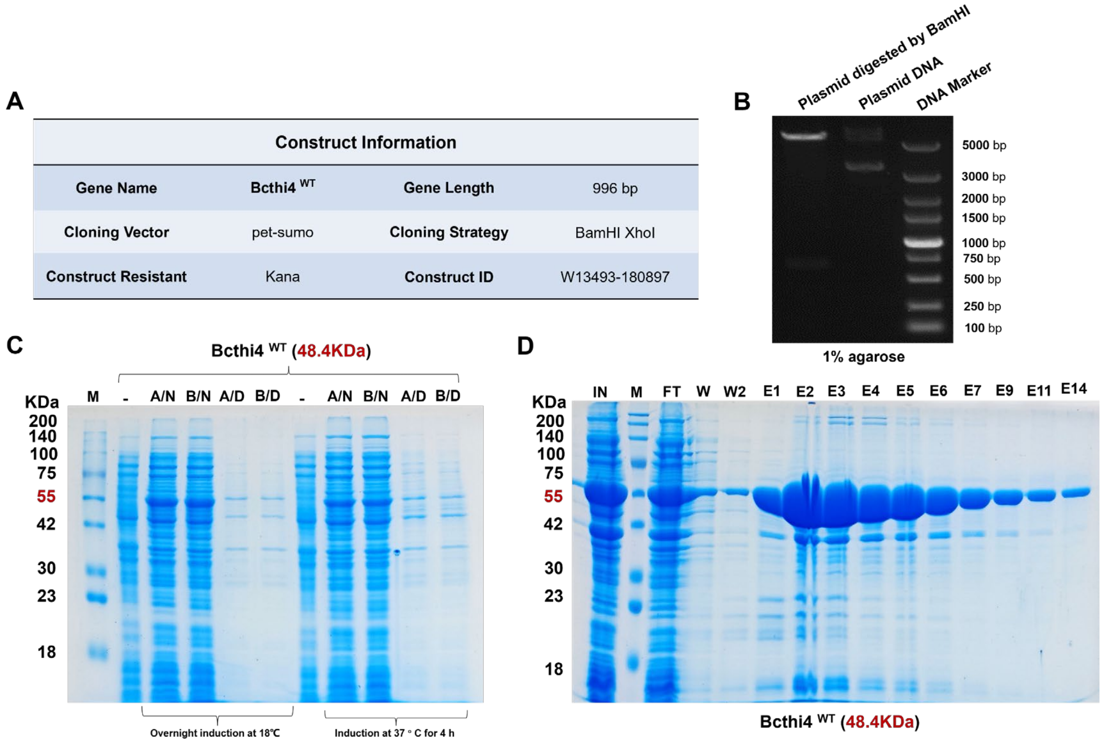

Construct Information			
Gene Name	Bcthi4 WT	Gene Length	996 bp
Cloning Vector	pet-sumo	Cloning Strategy	BamHI XhoI
Construct Resistant	Kana	Construct ID	W13493-180897

Fig. S4. Expression and purification of the Bcthi4 ^{WT} protein. (**A-B**) The construct information and enzyme digestion verification of Bcthi4 ^{WT} protein. (**C**) Bcthi4 ^{WT} protein expression test (Coomassie bright blue staining), M: protein marker, -: Not induced (negative control), A: BL21, B: T7E, N: Post-ultrasonic supernatant, D: post-ultrasonic precipitation. (**D**) Final concentration diagram of Bcthi4 ^{WT} protein purification (Coomassie bright blue staining). M: Protein marker, IN: Original sample, FT: Flow-through, W: Washing liquid, E: Eluate.

A

Construct Information			
**Gene Name**	**Bcthi4 ^{MT}**	**Gene Length**	996 bp
**Cloning Vector**	Pet-sumo	**Cloning Sites**	BamHI XhoI
**Construct Resistant**	Kana	**Construct ID**	W13493-180897
**Competence**	TOP10	**Vector Resistance**	Kan

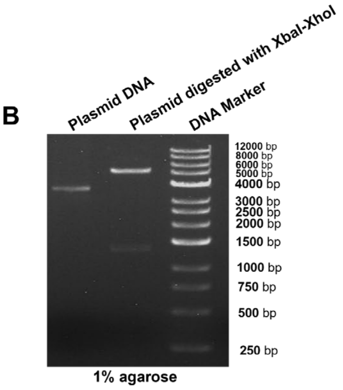

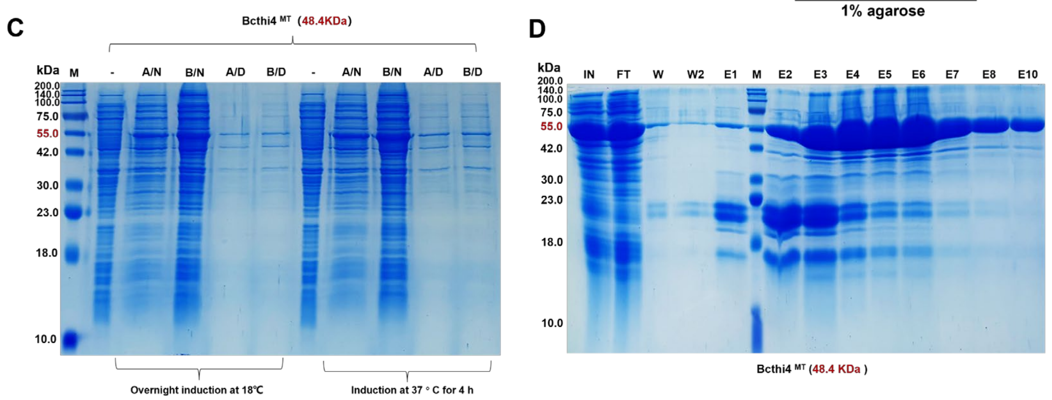

Fig. S5. Expression and purification of the Bcthi4 ^{MT} protein. (A-B) The construct information and enzyme digestion verification of Bcthi4 ^{MT} protein. (**C**) Bcthi4 ^{MT} protein expression test (Coomassie bright blue staining), M: protein marker, -: Not induced (negative control), A: BL21, B: T7E, N: Post-ultrasonic supernatant, D: post-ultrasonic precipitation. (**D**) Final concentration diagram of Bcthi4 ^{MT} protein purification (Coomassie bright blue staining). M: Protein marker, IN: Original sample, FT: Flow-through, W: Washing liquid, E: Eluate.

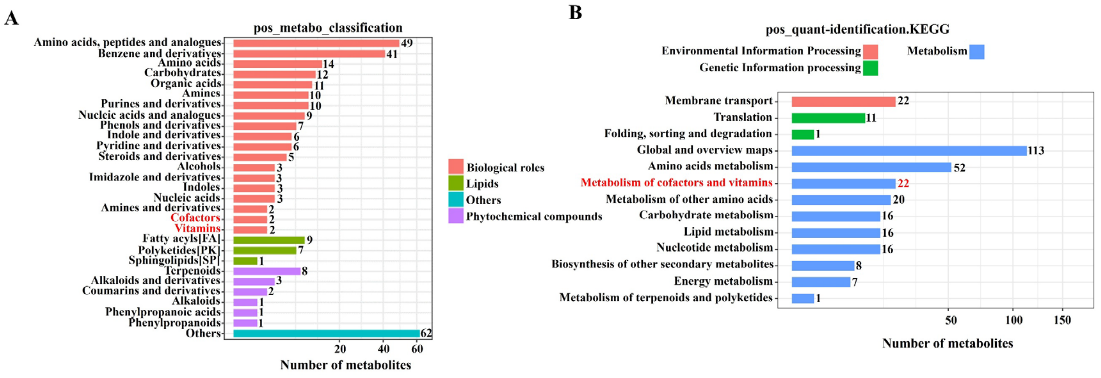

Fig. S6. Metabolomics analysis in positive ion mode. (**A**) pos-metabo classification (**B**) pos-quant identification KEGG.

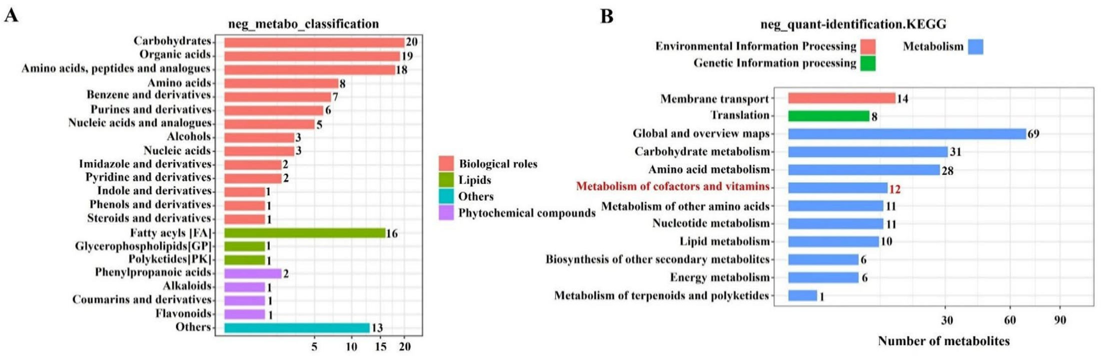

Fig. S7. Metabolomics analysis in negative ion mode. (**A**) neg-metabo classification (**B**) neg-quant identification KEGG.

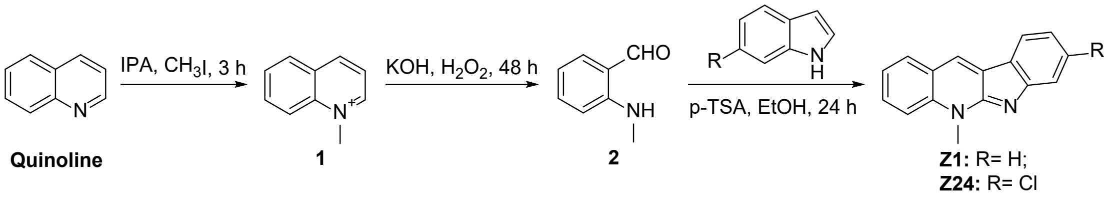

Fig. S8. Synthesis of the target compounds **Z1** and **Z24**.

Chemistry. The synthesis of intermediates and target compounds were performed as shown in Fig. S7. Briefly, quinoline was reacted with methyl iodide in isopropanol under inert gas to obtain the intermediate **1** with a high yield (>90 %). Subjecting intermediate **1** to hydrogen peroxide oxidation afforded the intermediate **2** without further purification. The intermediate **2** was reacted with substituted indoles to give the neocryptolepine (**Z1**) and its analogue (**Z24**) under the catalysis of *p*-toluenesulfonic acid. The target compounds were purified by column chromatography, and their structures were confirmed by ^{1}H and ^{13}C NMR and MS data.

Synthesis of the intermediate 1.

Under nitrogen, a mixture of quinoline (7.7 mmol) and MeI (11.6 mmol) in isopropanol (1 M) was heated at 90 °C for 3 h. The reaction was cooled to room temperature and the resulting precipitate was isolated by vacuum filtration, washed with a mixture of isopropanol/ethyl acetate (1:1), and dried in vacuo; and the obtained yellow solid was used for the next step.

Synthesis of the intermediate 2.

To a solution of potassium hydroxide (0.148 mol) in water (30 mL) and 1,2-dichloroethane (30 mL) was added the mixture of hydrogen peroxide (6.4 mL, 35 %) and intermediate **1** (15 mmol in 15 mL water) over 30 min at 0 °C. The resulting mixture was stirred at room temperature for 48 h, then the organic layer was separated, and the aqueous layer was extracted with dichloromethane (30 mL × 3). The combined organic layer was dried with anhydrous magnesium sulfate. The organic layer was concentrated under reduced pressure to obtain the title compound as a yellow oil for the next step.

Synthesis of the target compounds Z1 and Z24.

A mixture of intermediate **2** (5 mmol), indole (5 mmol), and p-TSA (5 mmol) in absolute ethanol (10 mL) was stirred open to air in a 50 mL round bottom flask. After reflux for 12 h, the reaction mixture was cooled to room temperature, washed with 1 M NaOH (50 mL) and the aqueous layer was extracted with CH_2Cl_2 (3 × 80 mL). The combined organic layer was then dried with anhydrous magnesium sulfate. The organic layer was concentrated under reduced pressure, and the residue was purified by column chromatography on silica gel, removing impurities with petroleum ether/ethyl acetate (2:1) and then eluting with dichloromethane/methanol (40:1) to yield the final compounds as a red solid.

Compound Z1

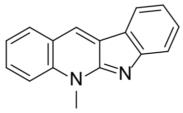

Yield, 62 %; red solid, m.p. 105.32-106.54 °C; ^{1}H NMR (400 MHz, DMSO-*d6*) δ 8.64 (s, 1H), 8.03 (d, *J* = 7.5 Hz, 1H), 7.94 (d, *J* = 8.0 Hz, 1H), 7.73 (d, *J* = 8.5 Hz, 1H), 7.68 (m, 1H), 7.61 (d, *J* = 7.9 Hz, 1H), 7.48 (t, *J* = 7.6 Hz, 1H), 7.36 (m, 1H), 7.15 (t, *J* = 7.4 Hz, 1H), 4.14 (s, 3H). ^{13}C NMR (101 MHz, DMSO-*d6*) δ 155.72, 155.70, 136.85, 130.81, 130.18, 129.13, 129.02, 127.24, 124.30, 122.01, 121.74, 120.52, 119.57, 117.61, 115.00, 32.96. MS-ESI m/z calcd for C17H14N2O[M+H]$^+$: 233.1530; found: 233.1548.

Compound Z24

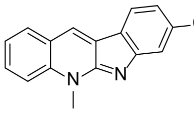

Yield, 51 %; yellow solid, m.p. 189.34-189.84 °C; ^{1}H NMR (400 MHz, DMSO-*d6*) *δ* 9.00 (s, 1H), 8.15 (m, 2H), 8.01 (d, *J* = 8.7 Hz, 1H), 7.88 (t, *J* = 8.0 Hz, 1H), 7.59 (s, 1H), 7.53 (t, *J* = 7.7 Hz, 1H), 7.19 (d, *J* = 8.1 Hz, 1H), 4.32 (s, 3H). ^{13}C NMR (101 MHz, DMSO-*d6*) *δ* 156.66, 137.03, 133.46, 131.48, 130.52, 130.37, 126.27, 123.07, 123.02, 122.71, 120.88, 119.48, 117.21, 115.64, 33.40. MS-ESI m/z: calcd for $C_{16}H_{11}ClN_2[M+H]^+$: 267.1418; found: 267.1422.
